# Predicting the northward expansion of tropical lineage *Rhipicephalus sanguineus* sensu lato ticks in the United States and its implications for medical and veterinary health

**DOI:** 10.1371/journal.pone.0271683

**Published:** 2022-08-24

**Authors:** Emily L. Pascoe, Santiago Nava, Marcelo B. Labruna, Christopher D. Paddock, Michael L. Levin, Matteo Marcantonio, Janet E. Foley

**Affiliations:** 1 Department of Medicine and Epidemiology, School of Veterinary Medicine, University of California, Davis, California, United States of America; 2 Laboratory of Entomology, Wageningen University & Research, Wageningen, The Netherlands; 3 Instituto Nacional de Tecnología Agropecuaria, Estación Experimental Agropecuaria Rafaela, Rafaela, Santa Fe, Argentina; 4 Consejo Nacional de Investigaciones Científcas y Técnicas (Conicet), Buenos Aires, Argentina; 5 Departamento de Medicina Veterinária Preventiva e Saúde Animal, Faculdade de Medicina Veterinária e Zootecnia, Universidade de São Paulo, São Paulo, Brasil; 6 Rickettsial Zoonoses Branch, Division of Vector-Borne Diseases, Centers for Disease Control and Prevention, United States Department of Health and Human Services, Atlanta, Georgia, United States of America; 7 Evolutionary Ecology and Genetics Group, Earth & Life Institute, UCLouvain, Louvain-la-Neuve, Belgium; Kansas State University College of Veterinary Medicine, UNITED STATES

## Abstract

The tropical lineage within the *Rhipicephalus sanguineus* species complex is cause for growing concern in the U.S. based on its prominent role in creating and perpetuating multiple recently identified outbreaks of Rocky Mountain spotted fever in the southwestern United States and northern Mexico. This lineage is undergoing a northward range expansion in the United States, necessitating the need for enhanced surveillance for *Rh*. *sanguineus*. To inform more focused surveillance efforts we use species distribution models (SDMs) to predict current (2015–2019) and future (2021–2040) habitat for the tropical lineage. Models using the MaxEnt algorithm were informed using geolocations of ticks genetically confirmed to be of the tropical lineage, for which data on 23 climatic and ecological variables were extracted. Models predicted that suitability was optimal where temperatures are relatively warm and stable, and there is minimal precipitation. This translated into habitat being predicted along much of the coast of southern states including California, Texas, Louisiana, and Florida. Although the endophilic nature of tropical *Rh*. *sanguineus* somewhat violates the assumptions of SDMs, our models correctly predicted known locations of this tick and provide a starting point for increased surveillance efforts. Furthermore, we highlight the importance of using molecular methods to distinguish between ticks in the *Rh*. *sanguineus* species complex.

## Introduction

*Rhipicephalus sanguineus* sensu lato (s.l.) is a globally widespread species complex comprising ticks of substantial human and veterinary medical importance [1]. These ticks are vectors of potentially life threatening agents of disease including *Rickettsia rickettsii*, *Rickettsia conorii*, and *Rickettsia massiliae*, as well as canine pathogens *Babesia vogeli* (agent of canine babesiosis) and *Ehrlichia canis* (agent of canine monocytic ehrlichiosis) [1–5]. *Rhipicephalus sanguineus* s.l. typically feed on hosts in the family Canidae earning it the common names “brown dog tick” and “kennel tick”; however, they will also occasionally bite humans [1].

**Fig 1:**
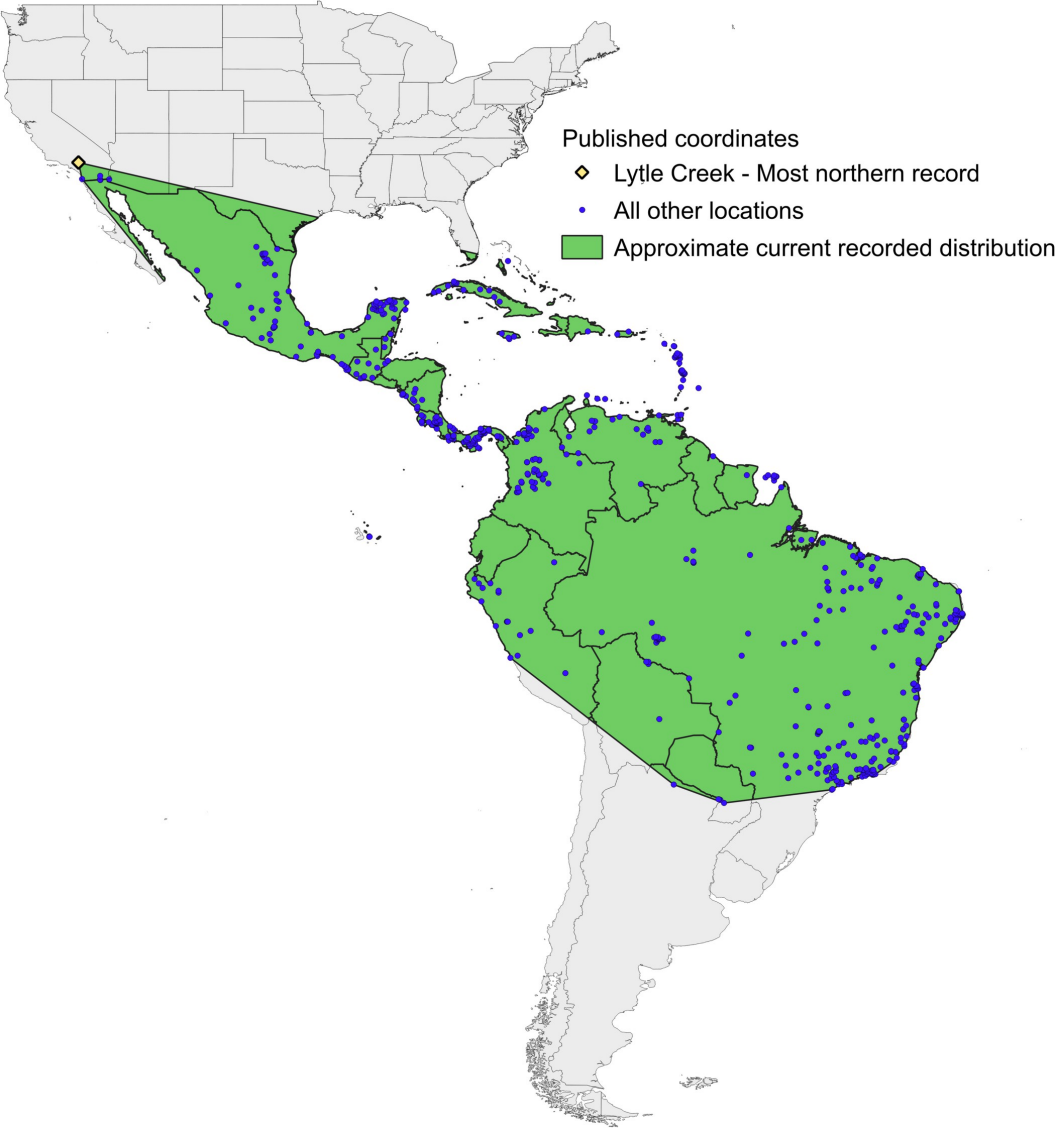
Approximation of current known distribution of tropical lineage *Rhipicephalus sanguineus*. The range of the tick was approximated using published coordinates of tropical lineage *Rhipicephalus sanguineus* confirmed by molecular methods, and the Minimum Bounding Geometry function in QGIS [17]. Lytle Creek, California, the location of the most northern record of the tick, is indicated.

The *Rh*. *sanguineus* s.l. species complex includes 12 taxa [6, 7], of which at least two are present in the Americas, namely *Rh*. *sanguineus* (Latreille, 1806) (or *Rh*. *sanguineus* sensu stricto, sometimes referred to as the “temperate lineage”; [6]) and the *Rh*. *sanguineus* sensu lato “tropical lineage” [6], henceforth referred to simply as “tropical *Rh*. *sanguineus*” or simply “tropical lineage”. In the Americas, the tropical lineage is reported to occur mostly between the Northern and Southern tropics where the mean annual temperature is >20°C, for example in the majority of Brazil, Central America, Mexico, and tropical northern Argentina, while *Rh*. *sanguineus* sensu stricto (s.s.) is typically distributed farther north and south where the mean annual temperature is <20°C, such as in the majority of the United States, southern Brazil, the majority of Argentina, Uruguay, and Chile (Fig 1) [6, 8–11]. Until now, tropical *Rh*. *sanguineus* have not been detected further north in the U.S. than Lytle Creek, situated near Los Angeles, California (Fig 1) [11]. Multiple studies indicate that *Rh*. *sanguineus* s.l. becomes more aggressive and has a higher affinity for humans as temperatures increase [12, 13]. However, in the laboratory the tropical lineage attaches more readily to a host than the temperate lineage [14]. The tropical lineage is a vector of *E*. *canis* [15] (primarily a pathogen of dogs, but also occasionally of humans and possibly cats) and has been implicated in a long-standing and highly lethal epidemic of Rocky Mountain spotted fever affecting both humans and dogs in northern Mexico [16].

Collections of *R*. *sanguineus* s.l. in California conducted during 2016–2017 identified the tropical lineage in Los Angeles County and farther south in San Diego County and Imperial County, whereas *Rh*. *sanguineus* s.s. were found to the north of these counties. Both lineages occurred sympatrically at Lytle Creek, San Bernardino County in southern California [11]. The presence of multiple tick lineages with differing human biting propensities, and the prospects for hybridization among lineages could influence risks and distribution of Rocky Mountain spotted fever across the western U.S., necessitating enhanced acaralogical surveillance to differentiate to lineage and to detect infected ticks [18]. Surveillance precision and efficiency can be informed using species distribution models (SDMs), which use ecological variables where a species is already established to predict the potential range of its expansion (e.g., [19, 20]). Predictions can also be made for the future by using projected climate data [20].

Here we use species distribution modeling to predict the current (2015–2019) and future (2021–2040) distribution range of the tropical *Rh*. *sanguineus* s.l. tick in the U.S. We predict that the potential range of tropical *Rh*. *sanguineus* in the U.S. is greater than the distribution characterized by observational studies, and that suitability will increase northward over the next 20 years due to favorable habitat resulting from climate change.

## Materials and methods

### Tropical lineage *Rhipicephalus sanguineus* geolocations

Data on tick presence were obtained from published literature and online databases. Briefly, the Web of Science core collection was searched for articles with “*Rhipicephalus sanguineus*” or “brown dog tick” in either the topic or title and all relevant publications documenting tick presence and location were selected from search results, as were references within. Latitude and longitude (or equivalent) coordinates were obtained or derived from descriptions of administrative boundaries (e.g., the name of a state park or municipality). Location data were also obtained from the Global Biodiversity Information Facility (GBIF; https://www.gbif.org/), Biodiversity Information Serving Our Nation (BISON; https://bison.usgs.gov/#home), and VectorMap (http://vectormap.si.edu/ [Accessed July 24 2019], see [21–23] for original data sources pertaining to this database). In addition, coordinate data on ticks either confirmed or inferred to be the tropical lineage based on molecular studies were provided by S. Nava and M. B. Labruna (e.g., see [8]). Geolocations are available in S1 Table of S1 File.

Data included in the model satisfied the following quality control measures: i) were submitted by a research institute, university, or other scientific organization (e.g., iNaturalist observations were excluded due to potential unreliability), ii) were collected between 1975–2019, iii) included two decimal places or more for at least one coordinate, and iv) had a coordinate inaccuracy of ≤1000 m if specified. Geolocations were verified to ensure that they represented the site of observation described. Preliminary results for SDMs that used geolocations for tropical *Rh*. *sanguineus* confirmed by either morphological or molecular methods as an input yielded unexpected results; e.g., areas in southern California, Arizona and Texas where the tick is known to be present were not predicted to be suitable, but areas that almost certainly are not suitable (e.g., the Sierra Nevada mountain range mainly situated in California, where high altitudes make for cold climatic conditions: average temperatures in July can be below 10°C above 1500 m elevation, and below 5°C above 2500 m elevation [24]) were predicted to be good habitat (see S1 File). We therefore posited that this dataset included geolocations of other cryptic tick species and elected to inform the model using only presence geolocations from ticks confirmed by molecular means (e.g., genetic sequencing) to be tropical *Rh*. *sanguineus*. Molecular confirmed geolocations were all from the Americas. These coordinates were used to create a map of the current known range of the tick which was approximated using the Minimum Bounding Geometry function in QGIS [17].

### Background geolocations

Background geolocations (a proxy for pseudo-absences) were generated in R V3.6.3 [25] and QGIS V3.4 [17] at a ratio of four presence to one background geolocation [26, 27]. Briefly, 75% of the total background geolocations were generated randomly within the country extents where presence geolocations were located, and 25% were randomly selected within kernel density estimates (KDE) with a 100 km bandwidth around presence geolocations. Using random background geolocations assumes that *Rh*. *sanguineus* is equally likely to be introduced at any location, as may be possible for ticks introduced by long distance means, such as livestock importation or host migration. On the other hand, including background geolocations based on KDE values can reduce oversampling bias in presence geolocations used to inform models, associated for example with sampling of sites that are easy to access [28]. Preliminary analyses indicated that the model performed best when 25% of background geolocations were randomly selected, likely in part due to the high density of presence geolocations in some areas. The gridSample function in the “dismo” package in R (V1.1–4, [29]) was used to retain only one presence or background geolocation within each predictor raster pixel (~1 km^2^) in order to minimize pseudo-replication and oversampling bias [29].

### Environmental predictor variables

A total of 23 environmental predictor variables were used in model training, and a subset of these variables was selected following model optimization (see below) to predict habitat for “current” and “future” climatic conditions. Future climatic conditions were derived from two Shared Socioeconomic Pathways (SSPs), which are reference pathways based on socio-economic scenarios for climate change, according to possible changes to society and ecosystems over a century timescale in the absence of climate change or climate policies [30, 31]. The environmental predictor variables comprised the 19 “bioclim” bioclimatic variables, plus elevation, slope, and the mean and standard deviation of normalized difference vegetation indices (NDVI). The time-span of the environmental predictor data was relevant to each model, i.e., data encompassing the time period for which tropical *Rh*. *sanguineus* observations were collected was used to train the model (1950–2000), and to make current and future predictions climate data for 2015–2019 and 2021–2040 were used respectively (Table 1).

**Table 1:** Sources of 23 environmental predictor variables used in species distribution modeling of the tropical lineage of *Rhipicephalus sanguineus*.

Environmental Predictor Variable	Model Training	Current (2015–2019) Climate Prediction	Future (2021–2040) Climate Prediction: SSP1–2.6	Future (2021–2040) Climate Prediction: SSP5–8.5
19 “bioclim” bioclimatic variables	WorldClim 2.0 dataset, 30 arc seconds: 1950–2000 [32]	Calculated from weather station data using parameter-elevation regressions on independent slopes model (PRISM) data, 2.5 arc minutes: 2015–2019 (PRISM Climate Group PRISM Gridded Climate Data)	Coupled Model Intercomparison Project Phase 6 downscaled future climate projections using seven general circulation models for SSP1–2.6: 2021–2040	Coupled Model Intercomparison Project Phase 6 downscaled future climate projections using seven general circulation models for SSP5–8.5: 2021–2040
Elevation	Consultative Group for International Agricultural Research-Shuttle Radar Topographic Mission dataset, 90 m	Same as model training	Same as model training	Same as model training
Slope	Consultative Group for International Agricultural Research-Shuttle Radar Topographic Mission dataset, 90 m	Same as model training	Same as model training	Same as model training
NDVI mean	National Oceanic and Atmospheric Administration STAR, 4 km: 1985–2000	National Oceanic and Atmospheric Administration STAR, 4 km: 2015–2019	Not used–future NDVI calculations not available	Not used–future NDVI calculations not available
NDVI standard deviation	National Oceanic and Atmospheric Administration STAR, 4 km: 1985–2000	National Oceanic and Atmospheric Administration STAR, 4 km: 2015–2019	Not used–future NDVI calculations not available	Not used–future NDVI calculations not available

Source of 19 bioclim indices, elevation, slope, and mean and standard deviation of normalized difference vegetation indices (NDVI) used in species distribution modeling of the tropical lineage of *Rhipicephalus sanguineus* in the United States. The time span of the environmental predictor data was relevant to the model, i.e., for model training data encompassing the time period of *Rh*. *sanguineus* observations used to train the model were used (1950–2000), and to make predictions for the “current” and “future” climate data for 2015–2019 and 2021–2040 were used respectively. As elevation and slope are not expected to change significantly over these time periods the same data for these variables were used for all models. As future projections for NDVI are not available these predictors were not used in models predicting habitat under future climatic conditions. All training and prediction environmental variables were re-sampled using nearest neighbor spatial interpolation to standardize resolution to 30 arc seconds (model training and present predictions) or 2.5 arc minutes (future predictions).

#### Bioclim variables

Bioclimatic variables have direct and indirect effects on habitat suitability for ticks. For model training, “bioclim” variables available from the WorldClim 2.0 data-set at 30 arc seconds resolution spanning 1950–2000 [32] were used. For current climate predictions, we used monthly minimum and maximum temperature and cumulative precipitation downloaded from the PRISM website for 2015–2019, from which bioclim variables were calculated at 30 arc seconds using the r.bioclim module in GRASS GIS V7.8 [33]. To predict habitat under future climatic conditions, data forecast for the Coupled Model Intercomparison Project Phase 6 (CMIP6) downscaled future climate projections for the years 2021–2040 were used [34]: Bioclim variables for seven general circulation models (GCM) were downloaded from WorldClim at 2.5 arc minutes. The seven GCMs were: the Beijing Climate Center Climate System Model (BCC-CSM2-MR) [35], the Centre National de Recherches Météorologiques (CNRM) and Centre Européen de Recherche et de Formation Avancée en Calcul Scientifique (CERFACS) for the sixth phase of the Coupled Model Intercomparison Project 6–1 model (CNRM-CM6-1) [36] and Earth System Model 2–1 (CNRM-ESM2-1) [37], the Canadian Earth System Model version 5 (CanESM5.0.3) [38], the Institut Pierre Simon Laplace Climate Model 6A –Low Resolution (IPSL-CM6A-LR) [39], the Model for Interdisciplinary Research on Climate, Earth System version 2 for Long-term simulations (MIROC-ES2L) [40], and the Model for Interdisciplinary Research on Climate—version 6 (MIROC6) [41]. These data were acquired for SSP 1–2.6 and SSP 5–8.5 [42]. SSP 1–2.6 predicts an optimistic scenario under which global warming is limited to below 2°C, with emissions at a higher starting point than some other SSPs (taking into account that emissions between 2007–2014 were higher than accounted for in some previous SSP scenarios), and a gradual decline in emissions [31]. This “best case scenario” sees greater levels of negative emissions in the later part of the century. SSP 5–8.5, considered the “worst case scenario”, considers high CO_2_ emissions continuing until 2040, after which emissions rapidly decline until negative emissions are observed in the late-century, resulting in a mean warming of 5.5°C [31]. To account for differences in the predictive abilities of the seven GCMs, mean values were calculated for each bioclim variable for each SSP. Given the high uncertainty already associated with using projected future climatic data, we did not interpolate these variables to the smaller spatial resolution (30 arc seconds) used for other environmental variables, and only performed models for future climatic conditions at a coarser spatial resolution (2.5 arc minutes).

#### Elevation & slope

Microclimate, host, and vegetation cover relevant to tick survival are all affected by elevation [43], and slope can serve as a proxy for soil moisture content due to its influence on the velocity of subsurface water flow and runoff rates [44, 45]. Elevation data were downloaded from the processed Consultative Group for International Agricultural Research-Shuttle Radar Topographic Mission data-set at 90 m resolution, and slope was calculated from elevation data using the r.slope.aspect module in GRASS GIS V7.8.0 [33, 46].

#### NDVI

NDVI is a measure of “greenness”, the long-term mean of which can be used as a proxy for vegetation cover over a given time period. The standard deviation of NDVI over time can be a proxy for land-use change [47], where higher values indicate a greater degree of change in greenness. For model training, NDVI data were downloaded from the National Oceanic and Atmospheric Administration STAR data-set for the years 1985–2000 at 4 km spatial resolution and 7-day composite temporal resolution. The same data but for the years 2015–2019 were used in models for current climate predictions. In both cases, the mean and standard deviation were calculated in GRASS GIS. To our knowledge, good predictions for NDVI in the U.S. for the future do not exist, thus NDVI variables were not used in future predictions.

Environmental variables were re-sampled using nearest neighbor spatial interpolation to standardize spatial resolution to 30 arc seconds (model training and present predictions) or 2.5 arc minutes (future predictions) and imported into R using the “raster” package (V3.0–7 [48]). Predictor variable values were extracted for each variable of the model training environmental data-set for each presence and background geolocation. Due to the complexity of coastlines and the comparatively coarse spatial resolution of the environmental data, environmental predictor raster files did not always cover presence and background geolocations near the coast. When a raster did not overlap a geolocation and environmental data could not be extracted, a distance matrix was applied to sample the nearest non-empty pixel within a 10 km radius [26].

### Modeling, evaluation and visualization

Models were run using the MaxEnt algorithm in the “dismo” package in R (V1.1–4 [29, 52]). Based on the knowledge and experience of the authors (to understand, optimize and implement the algorithm), and other similar studies [26, 49–51], MaxEnt was selected as the algorithm to be used in SDMs. The MaxEnt algorithm is deterministic and uses machine learning to make predictions based on presence and background geolocations (rather than true absence records) within the constraints derived from the environmental predictor data [52]. The algorithm uses maximum entropy to estimate the most uniform distribution of presence geolocations compared to background geolocations [52]. The output of model convergence describes how well the model fits the presence data compared to a uniform distribution of the species throughout the study extent [52, 53]. Model inputs were the environmental predictor variable data extracted from *Rh*. *sanguineus* presence and background geolocations. We opted against an ensemble model approach because preliminary analyses found that an ensemble model based on MaxEnt algorithm, along with random forest, boosted tree regression and generalized linear models made predictions that were deemed biologically much less feasible than those based on the MaxEnt algorithm itself. For example, the ensemble model was less successful at predicting suitable habitats at locations where the tick has already established in southern California. Conversely, the Death Valley National Park, which has an extremely hot and arid climate with considerable temperature fluctuations, and is therefore biologically unsuitable for this tick species, was predicted as one of the most suitable areas (see S1 File).

Bioclim variables that combined precipitation and temperature data (Mean Temperature of Wettest Quarter (BIO8), Mean Temperature of Driest Quarter (BIO9), Precipitation of Warmest Quarter (BIO18) and Precipitation of Coldest Quarter (BIO19)) were excluded following reports that these data have spatial artifacts that may affect modeling [54, 55]. Collinearity was assessed between each pair of environmental predictor variables using Pearson’s correlation coefficients. When the absolute value of the correlation coefficient was ≥0.80, just one of the two variables was retained in the model. This selection was informed by a combination of the ecological relevance of each variable to the tick (e.g., [1, 10]), and which of the two variables had the highest permutation importance in the model. Environmental predictor variables were refined until the model consisted of only non-correlated, biologically relevant variables (i.e., variables with a 0% permutation importance were removed). MaxEnt auto-feature classes were used and model regularization (betamultiplier) was set at 5 to minimize model over-fitting. Once the model had been optimized, a 10-fold cross-validation method was used to test performance and reduce variance of the final model [26]. Briefly, a seed was set and presence and background data were randomly partitioned into ten equal subsets. The model was iterated ten times, using a different combination of one test subset and nine training subsets for each iteration [26, 56, 57]. To reduce variance between the ten model iterations, the mean of the percent contribution and the permutation importance of each environmental predictor variable in the models was calculated, as was the mean of habitat suitability predictions [26, 57]. Model performance was evaluated using the mean area under the curve (AUC) of receiver operator characteristics from testing of the ten models. The higher the AUC value the greater the predictive capabilities of the model, whereby AUC values from 0–0.5 are considered to have predictive probabilities no better than random, and values from >0.5–1 indicate that the model predicts habitat suitability in locations of known presence better than by random [52]. Response curves (with GAM smoothing) were plotted indicating the correlation between environmental variables and habitat suitability. For each model, the mean suitability prediction for the ten iterations was visualized as i) a heatmap and, ii) converted into a binary representation of suitable habitat using a probability threshold that represented the mean maximum true positive rate at the maximum true negative rate across all ten models. The resulting binary raster classified each pixel as either suitable or unsuitable, with a score of 1 or 0, respectively.

## Results

### Tropical lineage *Rh*. *sanguineus* geolocations

Of 1306 quality-filtered *Rh*. *sanguineus* geolocations (S1 Table in S1 File), 593 had been confirmed as the tropical lineage using molecular methods. The optimal model using these coordinates comprised nine variables: three variables associated with temperature, two with precipitation, NDVI average, NDVI standard deviation, elevation, and slope (Table 2). Mean NDVI was the most important factor influencing habitat suitability (37.22%) followed by mean diurnal range (28.71%). Temperature seasonality, elevation, mean temperature of the coldest quarter, and slope each influenced the model by 5–10%, and the remaining three variables by >5%.

**Table 2:** Outputs from MaxEnt models predicting habitat suitability for the tropical lineage of *Rhipicephalus sanguineus*.

Environmental Predictor Variable	Percent Contribution	Permutation Importance
BIO2: Mean diurnal range (mean of monthly (max temp—min temp))	30.51	28.71
BIO4: Temperature seasonality (standard deviation *100)	7.88	8.78
BIO11: Mean temperature of coldest quarter	2.18	5.24
BIO12: Annual precipitation	1.56	2.80
BIO14: Precipitation of driest month	4.52	4.69
Elevation	12.35	6.71
Slope	1.62	5.21
NDVI average	38.82	37.22
NDVI standard deviation	0.55	0.65

Mean model outputs from 10 iterations of MaxEnt models to predict habitat suitability in the United States for the tropical lineage of *Rhipicephalus sanguineus*.

### Current habitat prediction

**Fig 2:**
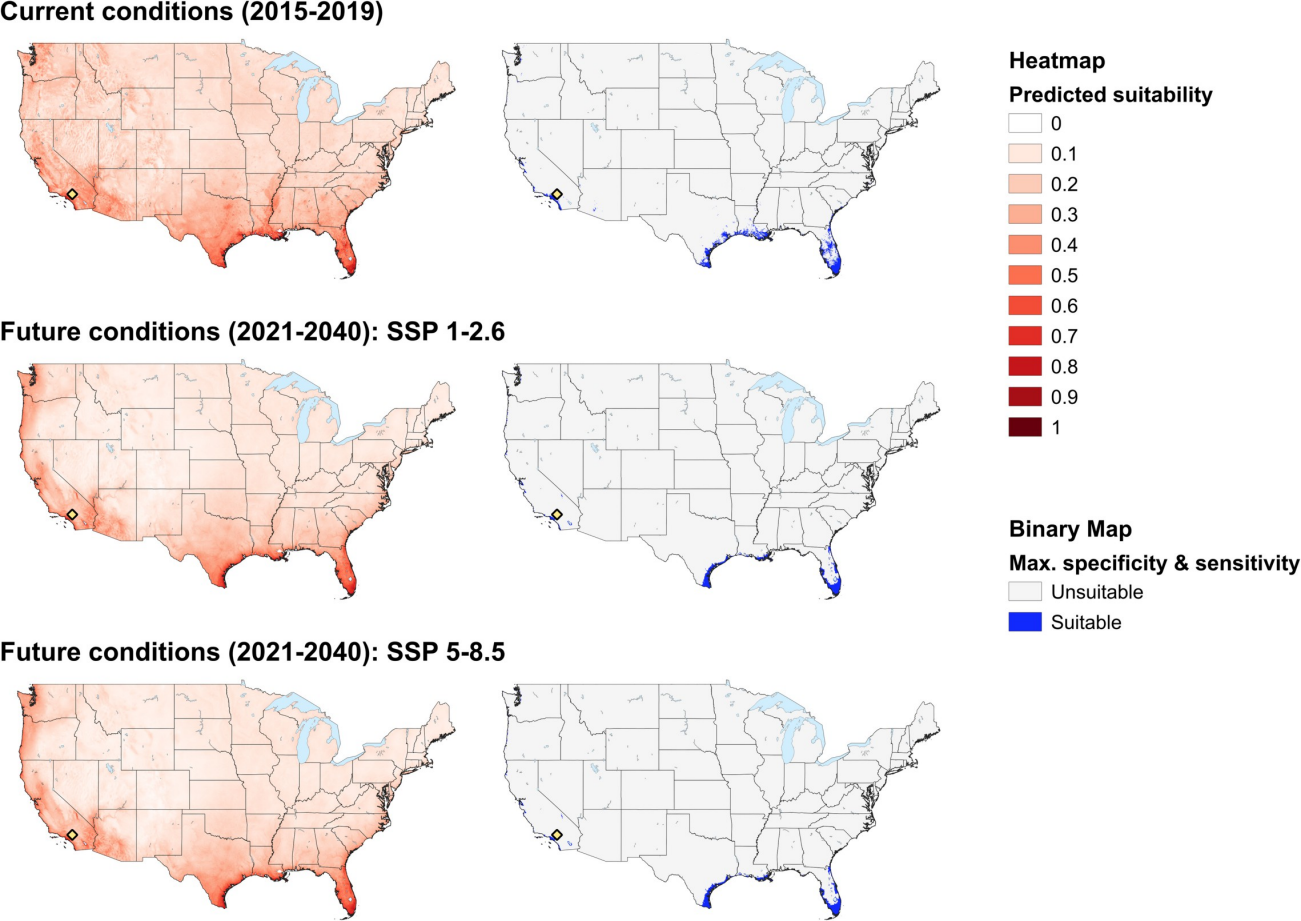
Tropical lineage *Rhipicephalus sanguineus* suitability maps. Habitat in the U.S. predicted (using MaxEnt species distribution modeling) to be environmentally suitable for the tropical lineage of *Rhipicephalus sanguineus* under current (2015–2019) climatic conditions, projected future (2021–2040) climatic conditions under the Shared Socioeconomic Pathway 1–2.6, and projected future (2021–2040) climatic conditions under the Shared Socioeconomic Pathway 5–8.5. Suitability is visualized as a heatmap and a binary probability threshold that represents the mean maximum true positive rate at the maximum true negative rate across all ten model iterations that were run for each scenario. Lytle Creek, California, the location of the most northern record of the tick, is indicated.

Following ten iterations, the optimal global model had a mean AUC of 0.72 (range = 0.67–0.77) and a mean maximum true positive rate + maximum true negative rate of 0.54 (0.46–0.65). The model predicted that habitat currently exists for tropical lineage *Rh*. *sanguineus* across approximately 173,600 km^2^ of the contiguous U.S., comprising a narrow but almost continuous strip of land along both the west and east coast (Fig 2). Much of the coastal south was suitable, mainly in Texas, Louisiana, Florida, and extending northward through Georgia and South Carolina. In addition, habitat was predicted throughout large portions of the southern coastal region of California, extending as far north as the San Francisco Bay area, with patchy habitat also present north thereof, thereby considerably extending northward the current known distribution of this tick (Fig 2).

**Fig 3:**
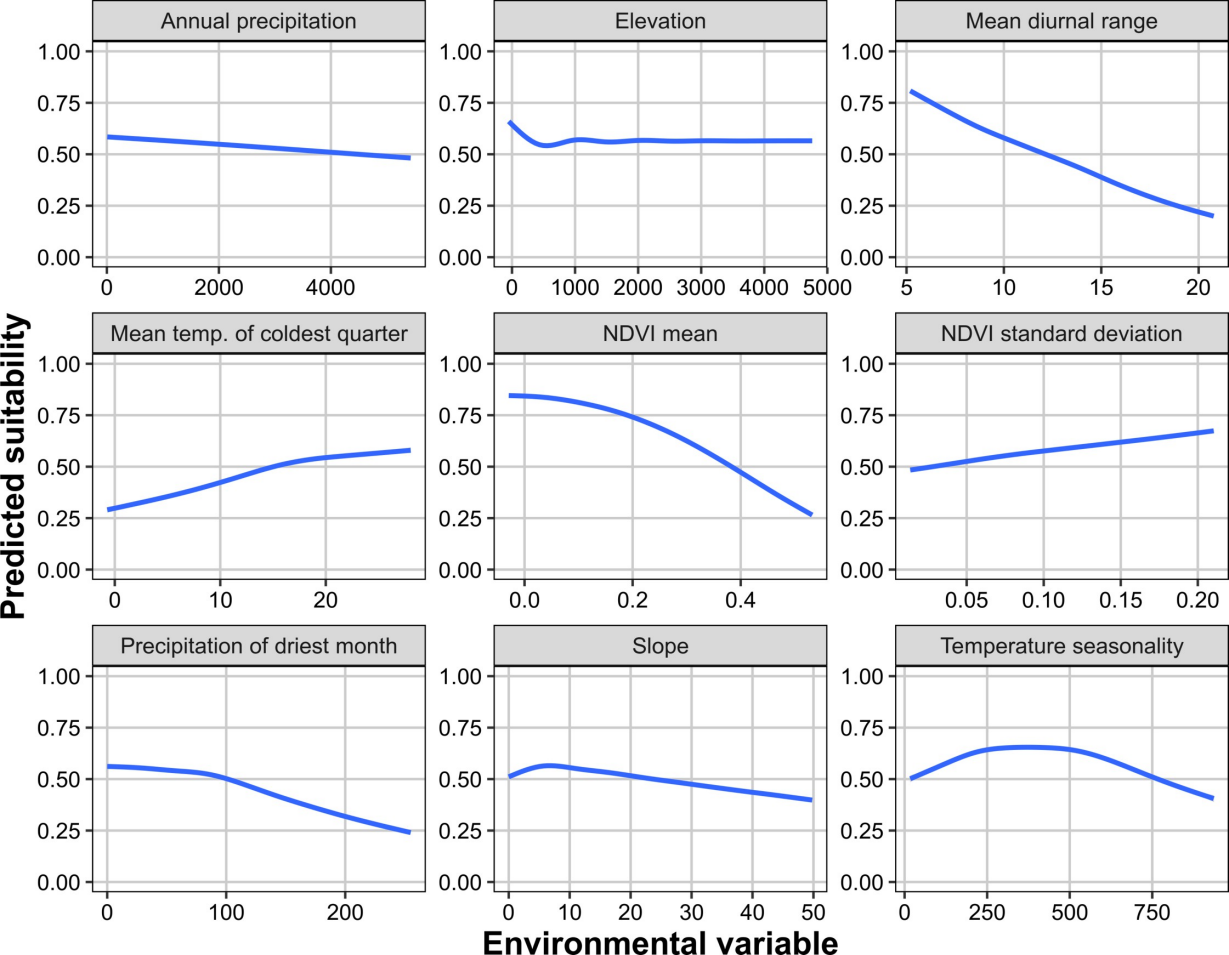
Tropical lineage *Rhipicephalus sanguineus* species distribution modeling response curves. Estimated smoothed trends (using GAM) between environmental predictor variables and predicted habitat suitability in MaxEnt models for the tropical lineage of *Rhipicephalus sanguineus*. Trends were derived from the ratio of probability density of each predictor at presence to background geolocations, considering data from 10 iterations used for model training and testing.

Based on response curves the most suitable locations for the tick appeared to be those where the temperature was relatively high and stable; mean temperature of the coldest quarter was positively correlated with suitability which was optimal (>50%) at temperatures of >15°C, whilst both mean diurnal range and temperature seasonality were negatively correlated with suitability (Fig 3). Indeed, habitat tended to occur where the mean temperature of the coldest quarter was at least 10°C and there was low variability in temperature, with temperature seasonality (temperature variation based on the standard deviation of monthly temperature averages) and mean diurnal range being relatively low (Fig 3). Lack of precipitation did not appear to be limiting within the model, and habitat remained at 50% or more suitability across the range of annual precipitation values. Precipitation during the driest month was negatively correlated with suitability, which dropped below 50% at locations that received more than 100 mm of precipitation during the driest month (Fig 3). However, when considering geographical areas where habitat was predicted, there was no clear association between areas of suitability and precipitation; predicted habitat on the west coast and in Arizona received as little as 500 mm or less of annual precipitation, but some habitat in Texas, Louisiana and Florida had 2000 mm or more. Similarly, precipitation of the driest month was relatively low at suitable areas on the west coast, but higher on the east coast (Fig 3). Response curves indicated that mean NDVI was also negatively correlated with suitability. As long as NDVI was <0.4, suitability remained above 50%. On the other hand, NDVI standard deviation (a proxy measure for land use change) was positively correlated with habitat suitability (Fig 3). Indeed, suitable areas were predicted where NDVI was typically 0.05–0.4, with the exception of a portion of Louisianan coastline, where suitable habitat was predicted in areas with a mean NDVI of ≤0.05, and the coast between Washington and north California where NDVI was comparatively high (≥0.4; Fig 3). Interestingly, habitat was predicted inland of Louisiana and Mississippi following the course of the Mississippi river (Fig 2). This coincided with a lower mean NDVI but higher NDVI standard deviation than the surrounding “unsuitable” habitat. There were no strong associations between suitability and elevation or slope, but habitat was rarely suitable where elevation was >500 m or slope was >5%.

### Future habitat prediction

**Fig 4:**
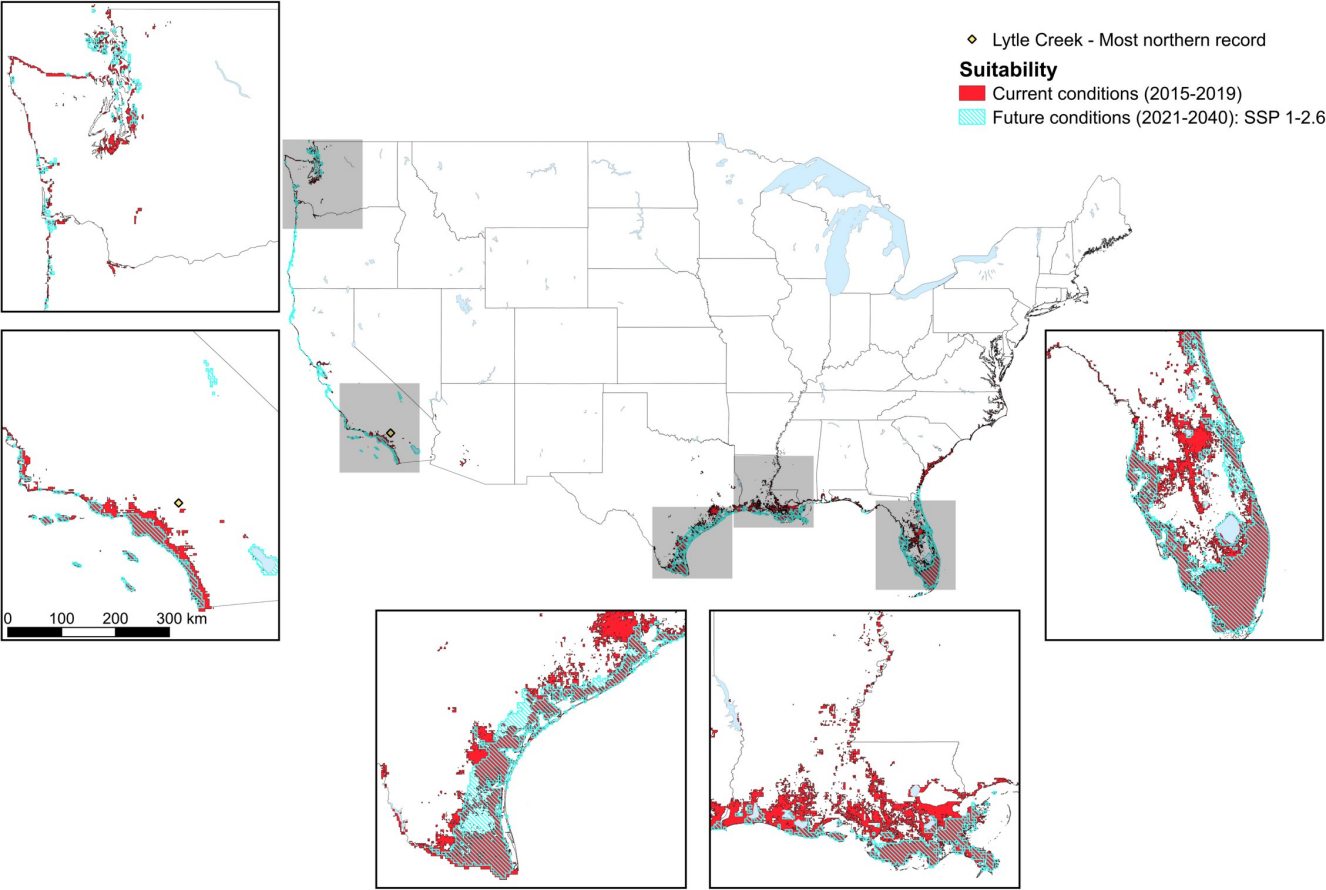
Overlap among tropical lineage *Rhipicephalus sanguineus* suitability maps under current and future conditions. The overlap in habitat in the U.S. predicted (using MaxEnt species distribution modeling) to be environmentally suitable for the tropical lineage of *Rhipicephalus sanguineus* under current (2015–2019) climatic conditions (red shading), and projected future (2021–2040) climatic conditions under the Shared Socioeconomic Pathway 1–2.6 (blue diagonal lines). Suitability is visualized as the mean maximum true positive rate at the maximum true negative rate across all ten model iterations run for each scenario. Larger areas of suitability are zoomed in to highlight the overlap and differences in suitability among predictions under current and future climate. Lytle Creek, California, the location of the most northern record of the tick, is indicated.

**Fig 5:**
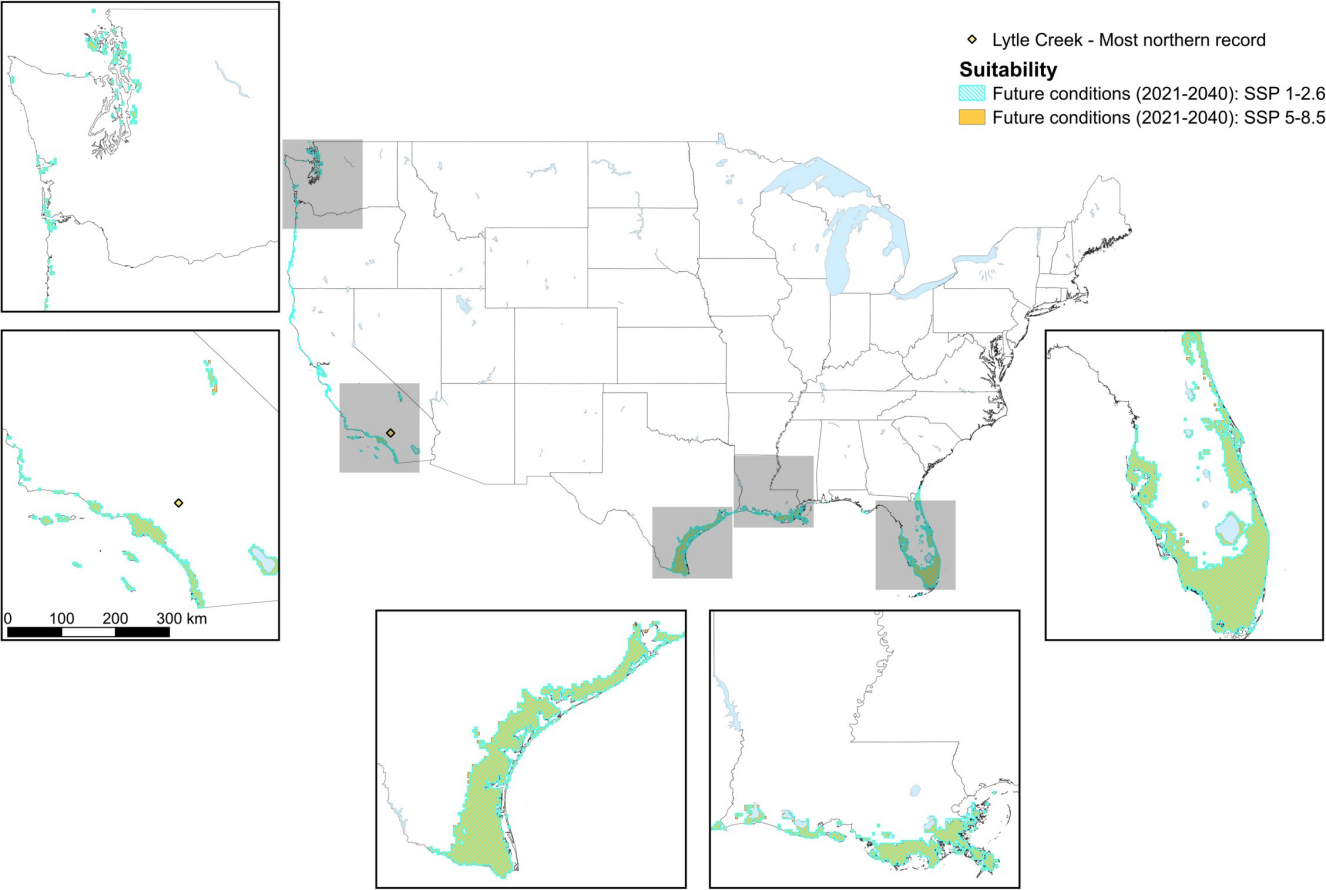
Overlap among tropical lineage *Rhipicephalus sanguineus* suitability maps under future conditions projected for two Shared Socioeconomic Pathways. The overlap in habitat in the U.S. predicted (using MaxEnt species distribution modeling) to be environmentally suitable for the tropical lineage of *Rhipicephalus sanguineus* under projected future (2021–2040) climatic conditions under the Shared Socioeconomic Pathway 1–2.6 (blue diagonal lines), and projected future (2021–2040) climatic conditions under the Shared Socioeconomic Pathway 5–8.5 (orange shading). Suitability is visualized as the mean maximum true positive rate at the maximum true negative rate across all ten model iterations run for each scenario. Larger areas of suitability are zoomed in to highlight the overlap and differences in suitability among Shared Socioeconomic Pathway scenarios. Lytle Creek, California, the location of the most northern record of the tick, is indicated.

For the SSP 1–2.6 scenario, the mean AUC for ten iterations of the model was 0.70 (0.66–0.76) and the mean maximum true positive rate + maximum true negative rate was 0.56 (0.49–0.62). Approximately 114,500 km^2^ were predicted suitable for the tick (~59,100 km^2^ less than current conditions; Fig 4). For SSP 5–8.5, the mean AUC for ten iterations of the model was 0.70 (0.66–0.76) and the mean maximum true positive rate + maximum true negative rate was 0.56 (0.49–0.62). An area of around 116,100 km^2^ was predicted to be suitable (57,500 km^2^ less than predicted for the current climate, but 1,600 km^2^ more than for the SSP 1–2.6 scenario). The geographic areas predicted to be suitable under the SSP 1–2.6 and SSP 5–8.5 scenario were very similar, but habitat under the SSP 5–8.5 scenario tended to extend further inland than for the SSP 1–2.6 scenario, particularly in the south (e.g., Texas and Florida; Fig 5).

Despite areas in the southwestern quarter of the state of Arizona being predicted as suitable for the tick under current climatic conditions, predictions under future climatic conditions were for lower levels of suitability for the tick in this area (Figs 2, 4 and 5). On the west coast, habitat south of the San Francisco Bay area extended further inland under current climatic conditions compared to future predictions. However, north of San Francisco, this pattern tended to be reversed (more inland habitat in the future), with the exception of the area around Seattle. Patchy habitat was available throughout the extent of the east coast for current climatic conditions, but for future conditions did not extend much further north than Florida. When compared with the current climate, habitat was more connected under future climatic conditions along the Texas and Florida coasts, which appeared to be associated with a decrease in precipitation and in temperature fluctuations between current and future climatic conditions. NDVI could only be used to make habitat predictions under current environmental conditions, as reliable future projections do not exist.

## Discussion

Here we use species distribution models to predict potential habitat in the U.S. for the tropical lineage of *Rh*. *sanguineus* s.l.. Although most geographical documentations of *Rh*. *sanguineus* do not distinguish between the different species within the complex, the available information indicates that the tropical lineage of *Rh*. *sanguineus* in the Americas inhabit areas mainly between the Northern and Southern Tropics, where mean annual temperatures generally exceed 20°C [9–11, 15]. In the U.S., the tropical lineage of *Rh*. *sanguineus* has previously been recorded in Florida, southern California, southwest Arizona, and southern Texas [11, 58]. Our models correctly predicted that habitat suitable for this tick exists in these four states; in addition, models show suitable habitat throughout coastal Texas, Louisiana and Florida; southern coastal California north to the San Francisco Bay; east into Arizona; and patchily along coasts of northern California, Oregon, and Washington, thereby considerably extending northward the potential distribution of this tick, which currently has been reported no further north than south San Bernardino county, California (Fig 1) [11].

Our results predict generally less available habitat for tropical *Rh*. *sanguineus* in the U.S. in the future, compared to predictions for the current climate. These differences could not be attributed to changes in a single climatic variable, but may have been associated with a future increase in the mean temperature of the coldest month in some coastal areas, but a decrease in this variable inland. On the other hand, increases in habitat in coastal Texas and Florida may occur due to a decrease in precipitation and in temperature fluctuations between current and future climatic conditions. However, due to differences in the spatial resolution between current and future climatic variables (current climate = 30 arc seconds and future climate = 2.5 arc minutes), the methods the different data-sets (used for model training and predictions at different timescales) apply to interpolate and calculate climatic data, and the inability to use future NDVI estimates in models predicting future habitat, we compare the extent of the predicted habitats with caution. When comparing future habitat predictions for the two SSP scenarios we did not see a large difference in the extent of suitable area; 1,600 km^2^ more land was predicted to be suitable under the “worst case” SSP 5–8.5 scenario. Interestingly, this included a small area within Death Valley, California, but mostly comprised areas slightly further inland from the Texas and Florida coast, although a notable area just inland from the Louisiana coast was actually more suitable for *Rh*. *sanguineus* under the “best case” SSP 1–2.6 scenario. There was no clear difference in a single variable between these two scenarios that was responsible for the difference in habitat extent, but in some locations (Texas, and some of Louisiana and Florida) a lower amount of annual precipitation under the “worst case” scenario allowed for more habitat inland.

One assumption of SDMs is that the distribution and abundance of the considered species are constrained by physiological limitations imposed by the environment. The tropical lineage of *Rh*. *sanguineus* primarily feeds on canids including domestic dogs, and is largely considered to be endophilic, exploiting the microclimate and proximity to food sources afforded by dog and human dwellings [59]—factors not directly incorporated into our models. Including environmental variables relevant to conditions in human and dog dwellings would be extremely challenging, given the vast diversity of factors that influence the indoor environment. For example, the type, size and material of the dwelling, level of insulation, presence and type of any indoor climate regulating device (e.g., central heating or AC), and personal preference of the inhabitants to maintain the indoor environment at a given temperature (e.g., see [60]). In many cases, particularly in lower socioeconomic situations, the indoor and outdoor environment may be highly similar. In addition, there is substantial evidence that the outdoor environment can be very important to *Rh*. *sanguineus* s.l. survival; considerable environmental infestations have been reported in Mexico and the southern U.S., where ticks have been observed living or walking on walls, both paved and dirt yards, and the peridomestic environment around homes [1, 61–63]. Despite the importance of the outdoor environment, trends that could also be relevant to the endophilic environment were observed, for example suitability was predicted to be the highest where the mean diurnal range (the mean of the monthly temperature ranges: monthly maximum minus monthly minimum) was the lowest, which can be interpreted as smaller temperature fluctuations being more favorable for the tick than larger fluctuations. Indeed, many residential houses are designed and built to minimize temperature fluctuations, and often successfully do so better than the external environment [64, 65], suggesting that at some locations homes may even provide a refugium for *Rh*. *sanguineus*. This concept can be extended to other temperature variables used in our models; ticks generally preferred warmer temperatures with minimal fluctuations, conditions that most households aim for in order to maintain comfort levels. As a dry-adapted tick the absence of precipitation within indoor environments is of no issue for tropical *Rh*. *sanguineus*, and in fact all relevant measures of precipitation included in our models were negatively correlated with habitat suitability. Although this could collectively point to an indoor environment being more suitable for the tick than the external environment we modeled (consequently potentially underestimating tropical *Rh*. *sanguineus* suitability), the absence in the models of variables related to the indoor environment should be considered when interpreting the suitability maps generated for this endophilic tick.

NDVI was used as an environmental predictor variable as a proxy of urbanization and disturbed landscapes, which often include the human-associated environments favored by this species. Smoothed trends between environmental predictor variables and predicted habitat suitability demonstrated that lower NDVI average values, and therefore less vegetation (and therefore possibly more urban environments), were associated with suitable habitat. In contrast, a higher NDVI standard deviation (a proxy for land-use change whereby higher values indicate a greater magnitude of change; [47]) tended to predict more suitable habitat. Indeed, while the suitable habitat was consistently along the coast and rarely inland, the primary exception to this rule was the presence of habitat along the course of the Mississippi River, where mean NDVI tended to be lower while NDVI standard deviation was higher than the surrounding “unsuitable” habitat. This may suggest that habitat occurs where vegetation cover has recently decreased, as often occurs during urbanization. However, suitability here may also be a consequence of the Mississippi River influencing the temperature and humidity of the local climate, such that it is more suitable for the tick than the surroundings (see [66]). The local climate associated with the river may in itself be also linked to NDVI, for example, the floodplain of the Mississippi River provides heat island features related to the intense agriculture occurring there, compared to adjacent mixed forest [67]. It was unfortunate that NDVI data could not be used in predicting future distributions of tropical lineage *Rh*. *sanguineus* s.l. As such, caution should be made in making direct comparisons between predicted habitat under current and future climatic conditions.

Our models captured the paradoxical ability of tropical lineage *Rh*. *sanguineus* ticks to tolerate drier climates due to water vapor absorption from relatively dry air (larvae), and water retention (adults) capabilities [68]. Response curves demonstrated that habitat suitability was greatest where annual precipitation and precipitation of the driest month were lowest, indicating that, provided there is adequate precipitation periodically throughout the year, the tick can survive. Contrary to other literature [8, 10, 69], we found that annual mean temperature is not an important predictor of habitat when other environmental variables are also taken into account. Rather, we show that warm temperatures during the coldest period of the year, combined with low annual and daily fluctuations in temperature, are key. Indeed, our model predicted that much of the coast in states in the south of the U.S. is suitable for the tropical lineage; coastal areas are often associated with milder and more stable temperature ranges than inland climates, and it is not unusual for this tick to be present in coastal areas of Central and South America. Laboratory studies document high mortality of this tick with prolonged exposure to 10°C, indicating that its distribution may be restricted by its poor ability to overwinter or for eggs to hatch in cooler climates [14]. We propose that the tropical lineage can survive at mean temperatures lower than 10°C provided there is a sufficient accumulation of degree-days above a given developmental threshold of temperature during each life stage, which is influenced by the amplitude of daily temperature oscillations.

During preliminary analyses, models were informed using worldwide but poorly verified geolocations for tropical *Rh*. *sanguineus* (see S1 File). However, when interpreting the suitability maps, we observed predicted habitat in unrealistic areas (e.g., the Sierra Nevada mountain range), whereas Arizona, Texas, and southern California, where known populations of tropical lineage *R*. *sanguineus* exist, were not considered suitable (see S1 File). We therefore restricted the analyses to a subset of the data which included only tropical ticks confirmed by molecular methods; all of which were from the Americas. While this greatly reduced the number of coordinates, the data-set was nevertheless sufficient to inform models (as demonstrated by mean AUC values of at least 0.70 for all models). It is possible that many reports of tropical *Rh*. *sanguineus* (particularly those outside of the Americas, for example observations in Africa and Asia) are actually of a different species, or that the tropical lineage present in the Americas has adapted to occupy a different ecological niche to that found elsewhere in the world. Either way, our study highlights the importance of confirming species and lineages using molecular methods rather than relying only on morphology, and making observations of ticks at novel locations publicly available so that researchers, vector control agencies and health professionals can use this knowledge to make well informed decisions regarding ticks and tick-borne pathogens in an area. As the common name “Gulf Coast tick” no longer seems suited to *Amblyomma maculatum* due to its establishment thousands of kilometers from the Gulf Coast, we posit that in the near future the terms “tropical” and “temperate” will be redundant when referring to the *Rh*. *sanguineus* lineages, as the tropical tick moves increasingly north of the tropics. With this in mind, attention should also be paid to any southward expansion of the topical lineage. Indeed, the need to increase tick surveillance efforts is not only important for monitoring the distribution of tropical *Rh*. *sanguineus*, but for other medically relevant tick species undergoing range expansion in the U.S., such as *Amblyomma americanum*, *A*. *maculatum*, *Dermacentor variabilis*, *Haemaphysalis longicornis*, and *Ixodes scapularis* [70, 71]. Due to the range of biotic and abiotic factors responsible for the increase in distribution of these tick species range expansion is likely to continue to cause disease risks to humans and animals.

## Conclusion

To conclude, our models indicate that tropical *Rh*. *sanguineus* has yet to occupy its entire potential range for current climatic conditions, although there is already evidence for the tick expanding in range [11]. Our study highlights the need for continuous vigilance and organized surveillance efforts which can be informed using our models to monitor a range of tick species of medical and veterinary concern. In addition, we demonstrate the importance of confirming tick identification stringently in order to describe current and future predicted risk of ticks and the pathogens they carry.

## Supporting information

S1 File Predicting the northward expansion of tropical lineage *Rhipicephalus sanguineus* sensu lato ticks in the United States and its implications for medical and veterinary health: Supporting information. (DOC)

S1 Graphical abstract (TIF)
