# Supplementary material for: Predicting the northward expansion of tropical lineage Rhipicephalus sanguineus sensu lato ticks in the United States and its implications for medical and veterinary health
Source: PLoS One. 2022 Aug 24;17(8):e0271683. doi: 10.1371/journal.pone.0271683 (PMC9401143; doi:10.1371/journal.pone.0271683)
Supplement: S1 File — (DOC) [file pone.0271683.s001.doc]

### Predicting the northward expansion of tropical lineage *Rhipicephalus sanguineus* sensu lato ticks in the United States and its implications for medical and veterinary health

### Supplementary Information

**Materials and methods**

**Geolocation selection for global *Rh. sanguineus*** **model**

Data included in the model satisfied the following quality control measures: i) were submitted by a research institute, university, or other scientific organization (e.g., iNaturalist observations were excluded due to potential unreliability), ii) were collected no less recently than 1975, iii) included two decimal places or more for at least one coordinate, and iv) had a coordinate inaccuracy of ≤1,000 m if specified (Table S1). Geolocations were cross-checked to ensure that they represented the site of observation described. Previous molecular identification and modeling of *Rh. sanguineus* has determined that the tropical lineage is present in areas where the annual mean temperature is >20°C, and they have been collected in American Samoa, Aruba, (central and northern) Brazil, Burkina Faso, Colombia, Costa Rica, Cuba, Djibouti, Ghana, Guam, Iraq, Japan, Kenya, the Marshall Islands, Mexico, Mozambique, Panama, Réunion, Saint Kitts, Taiwan, Thailand and Venezuela [1–3]. When there was no molecular confirmation of the *Rh. sanguineus* lineage for a geolocation we derived all areas worldwide where the annual mean temperature was >20°C between 1970–2000 from WorldClim data at a spatial resolution of 30 arc seconds, and merged them with the above-mentioned countries to form a single polygon in QGIS version 3.4 [4]. Tick geolocations that occurred within this “global tropical *Rh. sanguineus*” polygon were assumed to be of the tropical lineage, and all other geolocations were discarded. All other methods followed those in the materials and methods presented in the main manuscript.

**Table S1.** *Rhipicephalus sanguineus* geolocations used in a species distribution model to predict potential habitat for the tick in the U.S. Although 1,305 geolocations were used in the “global” model, only the 593 geolocations for which the tick was molecularly confirmed to be of the tropical lineage were used in the final model.

| **Latitude** | **Longitude** | **Data source** | **Taxonomic confirmation** | **Reference** |
| --- | --- | --- | --- | --- |
| 1.333 | 103.833 | BISON | Morphological or unknown | Albany Museum |
| 27.184385 | -82.36584 | BISON | Morphological or unknown | Biodiversity Information Serving Our Nation |
| -24.424167 | 27.353056 | BISON | Morphological or unknown | Brigham Young University Arthropod Museum |
| 25.03 | 121.48 | Literature | Morphological or unknown | [1] |
| 25.01 | 121.49 | Literature | Morphological or unknown | [1] |
| 25.02 | 121.5 | Literature | Morphological or unknown | [1] |
| 25.05 | 121.5 | Literature | Morphological or unknown | [1] |
| 25.13 | 121.5 | Literature | Morphological or unknown | [1] |
| 25.02 | 121.51 | Literature | Morphological or unknown | [1] |
| 25.05 | 121.51 | Literature | Morphological or unknown | [1] |
| 25.09 | 121.51 | Literature | Morphological or unknown | [1] |
| 25.14 | 121.51 | Literature | Morphological or unknown | [1] |
| 25.03 | 121.53 | Literature | Morphological or unknown | [1] |
| 25.09 | 121.53 | Literature | Morphological or unknown | [1] |
| 25.07 | 121.54 | Literature | Morphological or unknown | [1] |
| 25.11 | 121.55 | Literature | Morphological or unknown | [1] |
| 25.07 | 121.56 | Literature | Morphological or unknown | [1] |
| 25.08 | 121.56 | Literature | Morphological or unknown | [1] |
| 24.98 | 121.57 | Literature | Morphological or unknown | [1] |
| 25.02 | 121.57 | Literature | Morphological or unknown | [1] |
| 25.03 | 121.57 | Literature | Morphological or unknown | [1] |
| 25.09 | 121.58 | Literature | Morphological or unknown | [1] |
| 25.1 | 121.58 | Literature | Morphological or unknown | [1] |
| 25.08 | 121.6 | Literature | Morphological or unknown | [1] |
| 25.03 | 121.62 | Literature | Morphological or unknown | [1] |
| 18.939666 | -99.228202 | Literature | Morphological or unknown | [2] |
| 23.147926 | -82.144756 | Literature | Morphological or unknown | [3] |
| 22.963052 | -82.148209 | Literature | Morphological or unknown | [3] |
| 23.032213 | -82.264466 | Literature | Morphological or unknown | [3] |
| 23.007648 | -82.39999 | Literature | Morphological or unknown | [3] |
| 10.766667 | -61.233333 | BISON | Morphological or unknown | Denver Museum of Nature and Science |
| 23.47461 | -109.45762 | BISON | Morphological or unknown | Essig Museum of Entomology |
| 25.598889 | -100.158333 | BISON | Morphological or unknown | Facultad de Ciencias Biológicas de la Universidad Autónoma de Nuevo León |
| 25.785133 | -100.2921 | BISON | Morphological or unknown | Facultad de Ciencias Biológicas de la Universidad Autónoma de Nuevo León |
| 25.700667 | -100.297283 | BISON | Morphological or unknown | Facultad de Ciencias Biológicas de la Universidad Autónoma de Nuevo León |
| 25.729222 | -100.307306 | BISON | Morphological or unknown | Facultad de Ciencias Biológicas de la Universidad Autónoma de Nuevo León |
| 25.741389 | -100.450556 | BISON | Morphological or unknown | Facultad de Ciencias Biológicas de la Universidad Autónoma de Nuevo León |
| 20.576139 | -97.430639 | BISON | Morphological or unknown | Facultad de Ciencias Biológicas de la Universidad Autónoma de Nuevo León |
| 26.060556 | -98.243472 | BISON | Morphological or unknown | Facultad de Ciencias Biológicas de la Universidad Autónoma de Nuevo León |
| 23.736111 | -99.146111 | BISON | Morphological or unknown | Facultad de Ciencias Biológicas de la Universidad Autónoma de Nuevo León |
| 25.274167 | -99.6425 | BISON | Morphological or unknown | Facultad de Ciencias Biológicas de la Universidad Autónoma de Nuevo León |
| 25.78902 | -100.049529 | Literature | Morphological or unknown | [4] |
| 25.434132 | -100.159741 | Literature | Morphological or unknown | [4] |
| 25.700048 | -100.190654 | Literature | Morphological or unknown | [4] |
| 25.752755 | -100.204228 | Literature | Morphological or unknown | [4] |
| 25.677226 | -100.214421 | Literature | Morphological or unknown | [4] |
| 25.592085 | -100.252443 | Literature | Morphological or unknown | [4] |
| 25.759353 | -100.267853 | Literature | Morphological or unknown | [4] |
| 25.677833 | -100.314322 | Literature | Morphological or unknown | [4] |
| 25.805146 | -100.348672 | Literature | Morphological or unknown | [4] |
| 22.932778 | -82.386944 | Literature | Molecular | [5] |
| 34.729547 | 133.915817 | Literature | Morphological or unknown | [6] |
| 18.885833 | -100.128611 | BISON | Morphological or unknown | Instituto de Diagnóstico y Referencia Epidemiológicos |
| 25.539444 | -100.9475 | BISON | Morphological or unknown | Instituto de Diagnóstico y Referencia Epidemiológicos |
| 25.433333 | -101 | BISON | Morphological or unknown | Instituto de Diagnóstico y Referencia Epidemiológicos |
| 20.569167 | -101.1975 | BISON | Morphological or unknown | Instituto de Diagnóstico y Referencia Epidemiológicos |
| 26.900833 | -101.418055 | BISON | Morphological or unknown | Instituto de Diagnóstico y Referencia Epidemiológicos |
| 21.885556 | -102.384444 | BISON | Morphological or unknown | Instituto de Diagnóstico y Referencia Epidemiológicos |
| 25.854722 | -103.176389 | BISON | Morphological or unknown | Instituto de Diagnóstico y Referencia Epidemiológicos |
| 25.775 | -103.273055 | BISON | Morphological or unknown | Instituto de Diagnóstico y Referencia Epidemiológicos |
| 20.676389 | -103.346111 | BISON | Morphological or unknown | Instituto de Diagnóstico y Referencia Epidemiológicos |
| 20.720278 | -103.391944 | BISON | Morphological or unknown | Instituto de Diagnóstico y Referencia Epidemiológicos |
| 25.561111 | -103.498333 | BISON | Morphological or unknown | Instituto de Diagnóstico y Referencia Epidemiológicos |
| 20.238611 | -103.595 | BISON | Morphological or unknown | Instituto de Diagnóstico y Referencia Epidemiológicos |
| 25.888056 | -103.622222 | BISON | Morphological or unknown | Instituto de Diagnóstico y Referencia Epidemiológicos |
| 20.882778 | -103.836667 | BISON | Morphological or unknown | Instituto de Diagnóstico y Referencia Epidemiológicos |
| 25.080833 | -104.503611 | BISON | Morphological or unknown | Instituto de Diagnóstico y Referencia Epidemiológicos |
| 24.068333 | -104.643889 | BISON | Morphological or unknown | Instituto de Diagnóstico y Referencia Epidemiológicos |
| 21.591667 | -105.174167 | BISON | Morphological or unknown | Instituto de Diagnóstico y Referencia Epidemiológicos |
| 23.934722 | -105.266389 | BISON | Morphological or unknown | Instituto de Diagnóstico y Referencia Epidemiológicos |
| 21.679444 | -105.327222 | BISON | Morphological or unknown | Instituto de Diagnóstico y Referencia Epidemiológicos |
| 27.080833 | -109.445278 | BISON | Morphological or unknown | Instituto de Diagnóstico y Referencia Epidemiológicos |
| 27.203611 | -109.555 | BISON | Morphological or unknown | Instituto de Diagnóstico y Referencia Epidemiológicos |
| 23.241944 | -109.573611 | BISON | Morphological or unknown | Instituto de Diagnóstico y Referencia Epidemiológicos |
| 23.375 | -109.635278 | BISON | Morphological or unknown | Instituto de Diagnóstico y Referencia Epidemiológicos |
| 23.141944 | -109.646389 | BISON | Morphological or unknown | Instituto de Diagnóstico y Referencia Epidemiológicos |
| 23.061667 | -109.670833 | BISON | Morphological or unknown | Instituto de Diagnóstico y Referencia Epidemiológicos |
| 23.150278 | -109.683055 | BISON | Morphological or unknown | Instituto de Diagnóstico y Referencia Epidemiológicos |
| 23.135 | -109.689167 | BISON | Morphological or unknown | Instituto de Diagnóstico y Referencia Epidemiológicos |
| 23.18 | -109.701667 | BISON | Morphological or unknown | Instituto de Diagnóstico y Referencia Epidemiológicos |
| 23.150278 | -109.706111 | BISON | Morphological or unknown | Instituto de Diagnóstico y Referencia Epidemiológicos |
| 23.061389 | -109.708055 | BISON | Morphological or unknown | Instituto de Diagnóstico y Referencia Epidemiológicos |
| 23.131389 | -109.709444 | BISON | Morphological or unknown | Instituto de Diagnóstico y Referencia Epidemiológicos |
| 23.054722 | -109.709722 | BISON | Morphological or unknown | Instituto de Diagnóstico y Referencia Epidemiológicos |
| 23.1175 | -109.711111 | BISON | Morphological or unknown | Instituto de Diagnóstico y Referencia Epidemiológicos |
| 23.026667 | -109.717222 | BISON | Morphological or unknown | Instituto de Diagnóstico y Referencia Epidemiológicos |
| 23.069167 | -109.721111 | BISON | Morphological or unknown | Instituto de Diagnóstico y Referencia Epidemiológicos |
| 23.468889 | -109.741667 | BISON | Morphological or unknown | Instituto de Diagnóstico y Referencia Epidemiológicos |
| 23.184167 | -109.763611 | BISON | Morphological or unknown | Instituto de Diagnóstico y Referencia Epidemiológicos |
| 22.979722 | -109.771944 | BISON | Morphological or unknown | Instituto de Diagnóstico y Referencia Epidemiológicos |
| 23.444167 | -109.78 | BISON | Morphological or unknown | Instituto de Diagnóstico y Referencia Epidemiológicos |
| 23.471389 | -109.801944 | BISON | Morphological or unknown | Instituto de Diagnóstico y Referencia Epidemiológicos |
| 22.906389 | -109.887778 | BISON | Morphological or unknown | Instituto de Diagnóstico y Referencia Epidemiológicos |
| 22.889722 | -109.915555 | BISON | Morphological or unknown | Instituto de Diagnóstico y Referencia Epidemiológicos |
| 23.053056 | -109.915555 | BISON | Morphological or unknown | Instituto de Diagnóstico y Referencia Epidemiológicos |
| 22.923333 | -109.927778 | BISON | Morphological or unknown | Instituto de Diagnóstico y Referencia Epidemiológicos |
| 22.894167 | -109.945833 | BISON | Morphological or unknown | Instituto de Diagnóstico y Referencia Epidemiológicos |
| 23.9625 | -109.945833 | BISON | Morphological or unknown | Instituto de Diagnóstico y Referencia Epidemiológicos |
| 22.906944 | -109.948889 | BISON | Morphological or unknown | Instituto de Diagnóstico y Referencia Epidemiológicos |
| 22.936667 | -109.952222 | BISON | Morphological or unknown | Instituto de Diagnóstico y Referencia Epidemiológicos |
| 22.907778 | -109.956111 | BISON | Morphological or unknown | Instituto de Diagnóstico y Referencia Epidemiológicos |
| 23.203333 | -109.964444 | BISON | Morphological or unknown | Instituto de Diagnóstico y Referencia Epidemiológicos |
| 22.917778 | -109.980278 | BISON | Morphological or unknown | Instituto de Diagnóstico y Referencia Epidemiológicos |
| 23.077222 | -109.984722 | BISON | Morphological or unknown | Instituto de Diagnóstico y Referencia Epidemiológicos |
| 23.073889 | -110.024722 | BISON | Morphological or unknown | Instituto de Diagnóstico y Referencia Epidemiológicos |
| 23.762778 | -110.080833 | BISON | Morphological or unknown | Instituto de Diagnóstico y Referencia Epidemiológicos |
| 23.034722 | -110.081389 | BISON | Morphological or unknown | Instituto de Diagnóstico y Referencia Epidemiológicos |
| 23.693333 | -110.244167 | BISON | Morphological or unknown | Instituto de Diagnóstico y Referencia Epidemiológicos |
| 24.155 | -110.262222 | BISON | Morphological or unknown | Instituto de Diagnóstico y Referencia Epidemiológicos |
| 23.629444 | -110.275555 | BISON | Morphological or unknown | Instituto de Diagnóstico y Referencia Epidemiológicos |
| 24.020833 | -110.283333 | BISON | Morphological or unknown | Instituto de Diagnóstico y Referencia Epidemiológicos |
| 23.755278 | -110.286389 | BISON | Morphological or unknown | Instituto de Diagnóstico y Referencia Epidemiológicos |
| 24.070278 | -110.298889 | BISON | Morphological or unknown | Instituto de Diagnóstico y Referencia Epidemiológicos |
| 24.086944 | -110.313333 | BISON | Morphological or unknown | Instituto de Diagnóstico y Referencia Epidemiológicos |
| 24.061667 | -110.331389 | BISON | Morphological or unknown | Instituto de Diagnóstico y Referencia Epidemiológicos |
| 24.070833 | -110.348333 | BISON | Morphological or unknown | Instituto de Diagnóstico y Referencia Epidemiológicos |
| 24.583889 | -111.130833 | BISON | Morphological or unknown | Instituto de Diagnóstico y Referencia Epidemiológicos |
| 25.032222 | -111.670278 | BISON | Morphological or unknown | Instituto de Diagnóstico y Referencia Epidemiológicos |
| 25.165556 | -111.74 | BISON | Morphological or unknown | Instituto de Diagnóstico y Referencia Epidemiológicos |
| 32.534722 | -117.043611 | BISON | Morphological or unknown | Instituto de Diagnóstico y Referencia Epidemiológicos |
| 17.0125 | -95.0375 | BISON | Morphological or unknown | Instituto de Diagnóstico y Referencia Epidemiológicos |
| 16.818056 | -95.140278 | BISON | Morphological or unknown | Instituto de Diagnóstico y Referencia Epidemiológicos |
| 16.324444 | -95.238889 | BISON | Morphological or unknown | Instituto de Diagnóstico y Referencia Epidemiológicos |
| 17.067778 | -96.72 | BISON | Morphological or unknown | Instituto de Diagnóstico y Referencia Epidemiológicos |
| 21.170556 | -98.544722 | BISON | Morphological or unknown | Instituto de Diagnóstico y Referencia Epidemiológicos |
| 18.716667 | -98.775 | BISON | Morphological or unknown | Instituto de Diagnóstico y Referencia Epidemiológicos |
| 18.881111 | -98.851944 | BISON | Morphological or unknown | Instituto de Diagnóstico y Referencia Epidemiológicos |
| 21.148333 | -98.886111 | BISON | Morphological or unknown | Instituto de Diagnóstico y Referencia Epidemiológicos |
| 18.934722 | -98.897778 | BISON | Morphological or unknown | Instituto de Diagnóstico y Referencia Epidemiológicos |
| 18.787222 | -98.914444 | BISON | Morphological or unknown | Instituto de Diagnóstico y Referencia Epidemiológicos |
| 18.811389 | -98.921944 | BISON | Morphological or unknown | Instituto de Diagnóstico y Referencia Epidemiológicos |
| 18.791667 | -98.938611 | BISON | Morphological or unknown | Instituto de Diagnóstico y Referencia Epidemiológicos |
| 18.811667 | -98.954722 | BISON | Morphological or unknown | Instituto de Diagnóstico y Referencia Epidemiológicos |
| 18.878333 | -98.963611 | BISON | Morphological or unknown | Instituto de Diagnóstico y Referencia Epidemiológicos |
| 18.755833 | -98.975278 | BISON | Morphological or unknown | Instituto de Diagnóstico y Referencia Epidemiológicos |
| 18.6825 | -99.119444 | BISON | Morphological or unknown | Instituto de Diagnóstico y Referencia Epidemiológicos |
| 18.760833 | -99.121111 | BISON | Morphological or unknown | Instituto de Diagnóstico y Referencia Epidemiológicos |
| 19.275 | -99.138889 | BISON | Morphological or unknown | Instituto de Diagnóstico y Referencia Epidemiológicos |
| 18.918611 | -99.234167 | BISON | Morphological or unknown | Instituto de Diagnóstico y Referencia Epidemiológicos |
| -3.138353 | -60.024022 | BISON | Morphological or unknown | Instituto Nacional de Pesquisas da Amazônia |
| 25.75 | -100.02 | Personal comm. | Molecular | Nava and Labruna unpublished |
| 24.83 | -100.07 | Personal comm. | Molecular | Nava and Labruna unpublished |
| 25.42 | -100.15 | Personal comm. | Molecular | Nava and Labruna unpublished |
| 25.75 | -100.15 | Personal comm. | Molecular | Nava and Labruna unpublished |
| 25.68 | -100.25 | Personal comm. | Molecular | Nava and Labruna unpublished |
| 25.6 | -100.27 | Personal comm. | Molecular | Nava and Labruna unpublished |
| 25.68 | -100.3 | Personal comm. | Molecular | Nava and Labruna unpublished |
| 25.73 | -100.3 | Personal comm. | Molecular | Nava and Labruna unpublished |
| 17.85 | -100.37 | Personal comm. | Molecular | Nava and Labruna unpublished |
| 20.43 | -100.37 | Personal comm. | Molecular | Nava and Labruna unpublished |
| 26.38 | -100.87 | Personal comm. | Molecular | Nava and Labruna unpublished |
| 19.68 | -101.18 | Personal comm. | Molecular | Nava and Labruna unpublished |
| 20.68 | -101.35 | Personal comm. | Molecular | Nava and Labruna unpublished |
| 22.78 | -102.55 | Personal comm. | Molecular | Nava and Labruna unpublished |
| 19.23 | -103.73 | Personal comm. | Molecular | Nava and Labruna unpublished |
| 21.82 | -105.2 | Personal comm. | Molecular | Nava and Labruna unpublished |
| 24.17 | -106.42 | Personal comm. | Molecular | Nava and Labruna unpublished |
| -8 | -34.83 | Personal comm. | Molecular | Nava and Labruna unpublished |
| -7.93 | -34.87 | Personal comm. | Molecular | Nava and Labruna unpublished |
| -7.9 | -34.88 | Personal comm. | Molecular | Nava and Labruna unpublished |
| -8.05 | -34.88 | Personal comm. | Molecular | Nava and Labruna unpublished |
| -8.08 | -34.92 | Personal comm. | Molecular | Nava and Labruna unpublished |
| -8.18 | -34.92 | Personal comm. | Molecular | Nava and Labruna unpublished |
| -8 | -35 | Personal comm. | Molecular | Nava and Labruna unpublished |
| -5.83 | -35.18 | Personal comm. | Molecular | Nava and Labruna unpublished |
| -7.9 | -35.18 | Personal comm. | Molecular | Nava and Labruna unpublished |
| -5.85 | -35.23 | Personal comm. | Molecular | Nava and Labruna unpublished |
| -8 | -35.3 | Personal comm. | Molecular | Nava and Labruna unpublished |
| -7.65 | -35.33 | Personal comm. | Molecular | Nava and Labruna unpublished |
| -7.95 | -35.38 | Personal comm. | Molecular | Nava and Labruna unpublished |
| -7.87 | -35.45 | Personal comm. | Molecular | Nava and Labruna unpublished |
| -8.38 | -35.45 | Personal comm. | Molecular | Nava and Labruna unpublished |
| -7.58 | -35.48 | Personal comm. | Molecular | Nava and Labruna unpublished |
| -8.7 | -35.53 | Personal comm. | Molecular | Nava and Labruna unpublished |
| -8 | -35.58 | Personal comm. | Molecular | Nava and Labruna unpublished |
| -8.45 | -35.95 | Personal comm. | Molecular | Nava and Labruna unpublished |
| -8.28 | -35.98 | Personal comm. | Molecular | Nava and Labruna unpublished |
| -8.88 | -36.48 | Personal comm. | Molecular | Nava and Labruna unpublished |
| -10.22 | -36.85 | Personal comm. | Molecular | Nava and Labruna unpublished |
| -10.9 | -37.07 | Personal comm. | Molecular | Nava and Labruna unpublished |
| -8.07 | -37.27 | Personal comm. | Molecular | Nava and Labruna unpublished |
| -7.17 | -37.3 | Personal comm. | Molecular | Nava and Labruna unpublished |
| -7.02 | -37.32 | Personal comm. | Molecular | Nava and Labruna unpublished |
| -5.18 | -37.35 | Personal comm. | Molecular | Nava and Labruna unpublished |
| -8.27 | -38.03 | Personal comm. | Molecular | Nava and Labruna unpublished |
| -7.98 | -38.28 | Personal comm. | Molecular | Nava and Labruna unpublished |
| -12.97 | -38.5 | Personal comm. | Molecular | Nava and Labruna unpublished |
| -12.97 | -38.52 | Personal comm. | Molecular | Nava and Labruna unpublished |
| -8.8 | -38.57 | Personal comm. | Molecular | Nava and Labruna unpublished |
| -3.75 | -38.58 | Personal comm. | Molecular | Nava and Labruna unpublished |
| -3.77 | -38.58 | Personal comm. | Molecular | Nava and Labruna unpublished |
| -9.73 | -38.68 | Personal comm. | Molecular | Nava and Labruna unpublished |
| -4.23 | -38.73 | Personal comm. | Molecular | Nava and Labruna unpublished |
| -4.37 | -38.82 | Personal comm. | Molecular | Nava and Labruna unpublished |
| -4.25 | -38.88 | Personal comm. | Molecular | Nava and Labruna unpublished |
| -4.33 | -38.88 | Personal comm. | Molecular | Nava and Labruna unpublished |
| -9.48 | -38.88 | Personal comm. | Molecular | Nava and Labruna unpublished |
| -4.47 | -38.9 | Personal comm. | Molecular | Nava and Labruna unpublished |
| -4.23 | -38.92 | Personal comm. | Molecular | Nava and Labruna unpublished |
| -4.57 | -38.92 | Personal comm. | Molecular | Nava and Labruna unpublished |
| -4.27 | -38.93 | Personal comm. | Molecular | Nava and Labruna unpublished |
| -12.27 | -38.97 | Personal comm. | Molecular | Nava and Labruna unpublished |
| -12.53 | -38.97 | Personal comm. | Molecular | Nava and Labruna unpublished |
| -4.3 | -39 | Personal comm. | Molecular | Nava and Labruna unpublished |
| -14.95 | -39.02 | Personal comm. | Molecular | Nava and Labruna unpublished |
| -4.42 | -39.05 | Personal comm. | Molecular | Nava and Labruna unpublished |
| -14.87 | -39.07 | Personal comm. | Molecular | Nava and Labruna unpublished |
| -14.7 | -39.08 | Personal comm. | Molecular | Nava and Labruna unpublished |
| -9.6 | -39.1 | Personal comm. | Molecular | Nava and Labruna unpublished |
| -8.05 | -39.12 | Personal comm. | Molecular | Nava and Labruna unpublished |
| -14.65 | -39.18 | Personal comm. | Molecular | Nava and Labruna unpublished |
| -15.78 | -39.2 | Personal comm. | Molecular | Nava and Labruna unpublished |
| -14.68 | -39.27 | Personal comm. | Molecular | Nava and Labruna unpublished |
| -15.07 | -39.35 | Personal comm. | Molecular | Nava and Labruna unpublished |
| -14.45 | -39.4 | Personal comm. | Molecular | Nava and Labruna unpublished |
| -7.98 | -39.4 | Personal comm. | Molecular | Nava and Labruna unpublished |
| -14.5 | -39.47 | Personal comm. | Molecular | Nava and Labruna unpublished |
| -7.25 | -39.5 | Personal comm. | Molecular | Nava and Labruna unpublished |
| -7.77 | -39.93 | Personal comm. | Molecular | Nava and Labruna unpublished |
| -19.38 | -40.07 | Personal comm. | Molecular | Nava and Labruna unpublished |
| -18.38 | -40.12 | Personal comm. | Molecular | Nava and Labruna unpublished |
| -8.67 | -40.15 | Personal comm. | Molecular | Nava and Labruna unpublished |
| -20.13 | -40.3 | Personal comm. | Molecular | Nava and Labruna unpublished |
| -17.83 | -40.33 | Personal comm. | Molecular | Nava and Labruna unpublished |
| -9 | -40.33 | Personal comm. | Molecular | Nava and Labruna unpublished |
| -17.83 | -40.35 | Personal comm. | Molecular | Nava and Labruna unpublished |
| -18.7 | -40.38 | Personal comm. | Molecular | Nava and Labruna unpublished |
| -15.3 | -40.4 | Personal comm. | Molecular | Nava and Labruna unpublished |
| -18.75 | -40.4 | Personal comm. | Molecular | Nava and Labruna unpublished |
| -9.07 | -40.5 | Personal comm. | Molecular | Nava and Labruna unpublished |
| -9.4 | -40.5 | Personal comm. | Molecular | Nava and Labruna unpublished |
| -9.07 | -40.57 | Personal comm. | Molecular | Nava and Labruna unpublished |
| -19.93 | -40.6 | Personal comm. | Molecular | Nava and Labruna unpublished |
| -19.53 | -40.63 | Personal comm. | Molecular | Nava and Labruna unpublished |
| -6.4 | -40.73 | Personal comm. | Molecular | Nava and Labruna unpublished |
| -9.43 | -40.73 | Personal comm. | Molecular | Nava and Labruna unpublished |
| -21.4 | -41 | Personal comm. | Molecular | Nava and Labruna unpublished |
| -9.17 | -41.05 | Personal comm. | Molecular | Nava and Labruna unpublished |
| -20.85 | -41.12 | Personal comm. | Molecular | Nava and Labruna unpublished |
| -21.77 | -41.33 | Personal comm. | Molecular | Nava and Labruna unpublished |
| -12.78 | -41.42 | Personal comm. | Molecular | Nava and Labruna unpublished |
| -20.77 | -41.53 | Personal comm. | Molecular | Nava and Labruna unpublished |
| -20.83 | -41.53 | Personal comm. | Molecular | Nava and Labruna unpublished |
| -21.62 | -41.68 | Personal comm. | Molecular | Nava and Labruna unpublished |
| -9.6 | -42.12 | Personal comm. | Molecular | Nava and Labruna unpublished |
| -19.78 | -42.13 | Personal comm. | Molecular | Nava and Labruna unpublished |
| -22.25 | -42.52 | Personal comm. | Molecular | Nava and Labruna unpublished |
| -4.77 | -42.6 | Personal comm. | Molecular | Nava and Labruna unpublished |
| -5.1 | -42.78 | Personal comm. | Molecular | Nava and Labruna unpublished |
| -5.08 | -42.8 | Personal comm. | Molecular | Nava and Labruna unpublished |
| -20.23 | -42.82 | Personal comm. | Molecular | Nava and Labruna unpublished |
| -2.75 | -42.83 | Personal comm. | Molecular | Nava and Labruna unpublished |
| -5.3 | -42.85 | Personal comm. | Molecular | Nava and Labruna unpublished |
| -19.33 | -42.87 | Personal comm. | Molecular | Nava and Labruna unpublished |
| -22.93 | -43 | Personal comm. | Molecular | Nava and Labruna unpublished |
| -21.47 | -43.12 | Personal comm. | Molecular | Nava and Labruna unpublished |
| -11.08 | -43.15 | Personal comm. | Molecular | Nava and Labruna unpublished |
| -22.67 | -43.17 | Personal comm. | Molecular | Nava and Labruna unpublished |
| -22.9 | -43.18 | Personal comm. | Molecular | Nava and Labruna unpublished |
| -22.95 | -43.18 | Personal comm. | Molecular | Nava and Labruna unpublished |
| -22.97 | -43.18 | Personal comm. | Molecular | Nava and Labruna unpublished |
| -22.52 | -43.2 | Personal comm. | Molecular | Nava and Labruna unpublished |
| -22.92 | -43.2 | Personal comm. | Molecular | Nava and Labruna unpublished |
| -19.62 | -43.22 | Personal comm. | Molecular | Nava and Labruna unpublished |
| -3.55 | -43.25 | Personal comm. | Molecular | Nava and Labruna unpublished |
| -22.97 | -43.28 | Personal comm. | Molecular | Nava and Labruna unpublished |
| -22.55 | -43.3 | Personal comm. | Molecular | Nava and Labruna unpublished |
| -22.83 | -43.32 | Personal comm. | Molecular | Nava and Labruna unpublished |
| -22.92 | -43.33 | Personal comm. | Molecular | Nava and Labruna unpublished |
| -17.9 | -43.35 | Personal comm. | Molecular | Nava and Labruna unpublished |
| -21.73 | -43.35 | Personal comm. | Molecular | Nava and Labruna unpublished |
| -21.75 | -43.35 | Personal comm. | Molecular | Nava and Labruna unpublished |
| -3.73 | -43.35 | Personal comm. | Molecular | Nava and Labruna unpublished |
| -4.87 | -43.35 | Personal comm. | Molecular | Nava and Labruna unpublished |
| -19.5 | -43.5 | Personal comm. | Molecular | Nava and Labruna unpublished |
| -22.73 | -43.7 | Personal comm. | Molecular | Nava and Labruna unpublished |
| -22.9 | -43.7 | Personal comm. | Molecular | Nava and Labruna unpublished |
| -22.88 | -43.78 | Personal comm. | Molecular | Nava and Labruna unpublished |
| -22.47 | -43.82 | Personal comm. | Molecular | Nava and Labruna unpublished |
| -22.62 | -43.82 | Personal comm. | Molecular | Nava and Labruna unpublished |
| -22.37 | -43.88 | Personal comm. | Molecular | Nava and Labruna unpublished |
| -23.07 | -43.92 | Personal comm. | Molecular | Nava and Labruna unpublished |
| -19.9 | -43.93 | Personal comm. | Molecular | Nava and Labruna unpublished |
| -22.52 | -44.08 | Personal comm. | Molecular | Nava and Labruna unpublished |
| -22.55 | -44.1 | Personal comm. | Molecular | Nava and Labruna unpublished |
| -22.75 | -44.1 | Personal comm. | Molecular | Nava and Labruna unpublished |
| -22.55 | -44.17 | Personal comm. | Molecular | Nava and Labruna unpublished |
| -2.57 | -44.2 | Personal comm. | Molecular | Nava and Labruna unpublished |
| -23.15 | -44.23 | Personal comm. | Molecular | Nava and Labruna unpublished |
| -2.53 | -44.3 | Personal comm. | Molecular | Nava and Labruna unpublished |
| -2.65 | -44.3 | Personal comm. | Molecular | Nava and Labruna unpublished |
| -19.88 | -44.33 | Personal comm. | Molecular | Nava and Labruna unpublished |
| -3.38 | -44.35 | Personal comm. | Molecular | Nava and Labruna unpublished |
| -5.58 | -44.38 | Personal comm. | Molecular | Nava and Labruna unpublished |
| -2.4 | -44.42 | Personal comm. | Molecular | Nava and Labruna unpublished |
| -22.47 | -44.45 | Personal comm. | Molecular | Nava and Labruna unpublished |
| -22.5 | -44.72 | Personal comm. | Molecular | Nava and Labruna unpublished |
| -2.7 | -44.82 | Personal comm. | Molecular | Nava and Labruna unpublished |
| -21.23 | -45 | Personal comm. | Molecular | Nava and Labruna unpublished |
| -21.25 | -45 | Personal comm. | Molecular | Nava and Labruna unpublished |
| -2.52 | -45.08 | Personal comm. | Molecular | Nava and Labruna unpublished |
| -5.5 | -45.25 | Personal comm. | Molecular | Nava and Labruna unpublished |
| -18.33 | -45.28 | Personal comm. | Molecular | Nava and Labruna unpublished |
| -23.25 | -45.3 | Personal comm. | Molecular | Nava and Labruna unpublished |
| -1.67 | -45.37 | Personal comm. | Molecular | Nava and Labruna unpublished |
| -21.08 | -45.57 | Personal comm. | Molecular | Nava and Labruna unpublished |
| -15.32 | -45.6 | Personal comm. | Molecular | Nava and Labruna unpublished |
| -15.35 | -45.8 | Personal comm. | Molecular | Nava and Labruna unpublished |
| -4.08 | -45.95 | Personal comm. | Molecular | Nava and Labruna unpublished |
| -20 | -45.97 | Personal comm. | Molecular | Nava and Labruna unpublished |
| -5.82 | -45.98 | Personal comm. | Molecular | Nava and Labruna unpublished |
| -7.53 | -46.03 | Personal comm. | Molecular | Nava and Labruna unpublished |
| -23.85 | -46.13 | Personal comm. | Molecular | Nava and Labruna unpublished |
| -23.65 | -46.18 | Personal comm. | Molecular | Nava and Labruna unpublished |
| -20.3 | -46.3* | Personal comm. | Molecular | Nava and Labruna unpublished |
| -20.3 | -46.43 | Personal comm. | Molecular | Nava and Labruna unpublished |
| -23.87 | -46.43 | Personal comm. | Molecular | Nava and Labruna unpublished |
| -23.63 | -46.52 | Personal comm. | Molecular | Nava and Labruna unpublished |
| -23.53 | -46.63 | Personal comm. | Molecular | Nava and Labruna unpublished |
| -23.55 | -46.63 | Personal comm. | Molecular | Nava and Labruna unpublished |
| -22.65 | -46.68 | Personal comm. | Molecular | Nava and Labruna unpublished |
| -22.32 | -46.7 | Personal comm. | Molecular | Nava and Labruna unpublished |
| -22.18 | -46.75 | Personal comm. | Molecular | Nava and Labruna unpublished |
| -23.18 | -46.87 | Personal comm. | Molecular | Nava and Labruna unpublished |
| -23.18 | -46.88 | Personal comm. | Molecular | Nava and Labruna unpublished |
| -22.73 | -46.9 | Personal comm. | Molecular | Nava and Labruna unpublished |
| -22.73 | -46.95 | Personal comm. | Molecular | Nava and Labruna unpublished |
| -24.32 | -47 | Personal comm. | Molecular | Nava and Labruna unpublished |
| -24.38 | -47.07 | Personal comm. | Molecular | Nava and Labruna unpublished |
| -22.92 | -47.08 | Personal comm. | Molecular | Nava and Labruna unpublished |
| -23.3 | -47.13 | Personal comm. | Molecular | Nava and Labruna unpublished |
| -7.65 | -47.3 | Personal comm. | Molecular | Nava and Labruna unpublished |
| -5.75 | -47.37 | Personal comm. | Molecular | Nava and Labruna unpublished |
| -22.32 | -47.38 | Personal comm. | Molecular | Nava and Labruna unpublished |
| -20.53 | -47.4 | Personal comm. | Molecular | Nava and Labruna unpublished |
| -5.55 | -47.43 | Personal comm. | Molecular | Nava and Labruna unpublished |
| -23.5 | -47.47 | Personal comm. | Molecular | Nava and Labruna unpublished |
| -16.58 | -47.48 | Personal comm. | Molecular | Nava and Labruna unpublished |
| -5.52 | -47.48 | Personal comm. | Molecular | Nava and Labruna unpublished |
| -4.95 | -47.5 | Personal comm. | Molecular | Nava and Labruna unpublished |
| -22.4 | -47.55 | Personal comm. | Molecular | Nava and Labruna unpublished |
| -22.7 | -47.63 | Personal comm. | Molecular | Nava and Labruna unpublished |
| -3.63 | -47.68 | Personal comm. | Molecular | Nava and Labruna unpublished |
| -21.2 | -47.82 | Personal comm. | Molecular | Nava and Labruna unpublished |
| -1.3 | -47.92 | Personal comm. | Molecular | Nava and Labruna unpublished |
| -18.95 | -48.2 | Personal comm. | Molecular | Nava and Labruna unpublished |
| -7.18 | -48.2 | Personal comm. | Molecular | Nava and Labruna unpublished |
| -18.9 | -48.27 | Personal comm. | Molecular | Nava and Labruna unpublished |
| -18.92 | -48.28 | Personal comm. | Molecular | Nava and Labruna unpublished |
| -21.25 | -48.28 | Personal comm. | Molecular | Nava and Labruna unpublished |
| -18.98 | -48.3 | Personal comm. | Molecular | Nava and Labruna unpublished |
| -21.92 | -48.32 | Personal comm. | Molecular | Nava and Labruna unpublished |
| -19 | -48.33 | Personal comm. | Molecular | Nava and Labruna unpublished |
| -19.17 | -48.38 | Personal comm. | Molecular | Nava and Labruna unpublished |
| -10.7 | -48.42 | Personal comm. | Molecular | Nava and Labruna unpublished |
| -22.88 | -48.43 | Personal comm. | Molecular | Nava and Labruna unpublished |
| -20.93 | -48.48 | Personal comm. | Molecular | Nava and Labruna unpublished |
| -8.53 | -48.5 | Personal comm. | Molecular | Nava and Labruna unpublished |
| -1.02 | -48.97 | Personal comm. | Molecular | Nava and Labruna unpublished |
| -22.33 | -49.08 | Personal comm. | Molecular | Nava and Labruna unpublished |
| -16.68 | -49.25 | Personal comm. | Molecular | Nava and Labruna unpublished |
| -16.63 | -49.27 | Personal comm. | Molecular | Nava and Labruna unpublished |
| -9.8 | -49.65 | Personal comm. | Molecular | Nava and Labruna unpublished |
| -1.03 | -49.93 | Personal comm. | Molecular | Nava and Labruna unpublished |
| -22.78 | -50.2 | Personal comm. | Molecular | Nava and Labruna unpublished |
| -15.07 | -50.42 | Personal comm. | Molecular | Nava and Labruna unpublished |
| -21.2 | -50.43 | Personal comm. | Molecular | Nava and Labruna unpublished |
| -18.45 | -50.45 | Personal comm. | Molecular | Nava and Labruna unpublished |
| -10.55 | -50.55 | Personal comm. | Molecular | Nava and Labruna unpublished |
| 0.08 | -51.07 | Personal comm. | Molecular | Nava and Labruna unpublished |
| -23.3 | -51.15 | Personal comm. | Molecular | Nava and Labruna unpublished |
| -22.12 | -51.38 | Personal comm. | Molecular | Nava and Labruna unpublished |
| -10.73 | -51.55 | Personal comm. | Molecular | Nava and Labruna unpublished |
| -21.1 | -51.77 | Personal comm. | Molecular | Nava and Labruna unpublished |
| 3.9 | -51.8 | Personal comm. | Molecular | Nava and Labruna unpublished |
| 4.9 | -52.25 | Personal comm. | Molecular | Nava and Labruna unpublished |
| 4.93 | -52.32 | Personal comm. | Molecular | Nava and Labruna unpublished |
| 4.52 | -52.47 | Personal comm. | Molecular | Nava and Labruna unpublished |
| 5 | -52.47 | Personal comm. | Molecular | Nava and Labruna unpublished |
| 4.82 | -52.52 | Personal comm. | Molecular | Nava and Labruna unpublished |
| 5.02 | -53.03 | Personal comm. | Molecular | Nava and Labruna unpublished |
| 4.82 | -53.27 | Personal comm. | Molecular | Nava and Labruna unpublished |
| -22.88 | -54.45 | Personal comm. | Molecular | Nava and Labruna unpublished |
| -20.45 | -54.62 | Personal comm. | Molecular | Nava and Labruna unpublished |
| -20.45 | -54.65 | Personal comm. | Molecular | Nava and Labruna unpublished |
| -2.43 | -54.72 | Personal comm. | Molecular | Nava and Labruna unpublished |
| -20.43 | -54.73 | Personal comm. | Molecular | Nava and Labruna unpublished |
| -9.78 | -54.9 | Personal comm. | Molecular | Nava and Labruna unpublished |
| 5.03 | -55.02 | Personal comm. | Molecular | Nava and Labruna unpublished |
| -11.85 | -55.5 | Personal comm. | Molecular | Nava and Labruna unpublished |
| -15.58 | -56.08 | Personal comm. | Molecular | Nava and Labruna unpublished |
| -16.25 | -56.62 | Personal comm. | Molecular | Nava and Labruna unpublished |
| -25.62 | -57.15 | Personal comm. | Molecular | Nava and Labruna unpublished |
| -25.33 | -57.5 | Personal comm. | Molecular | Nava and Labruna unpublished |
| -25.28 | -57.62 | Personal comm. | Molecular | Nava and Labruna unpublished |
| -19 | -57.65 | Personal comm. | Molecular | Nava and Labruna unpublished |
| -25.27 | -57.67 | Personal comm. | Molecular | Nava and Labruna unpublished |
| 6.8 | -58.17 | Personal comm. | Molecular | Nava and Labruna unpublished |
| 13.17 | -59.53 | Personal comm. | Molecular | Nava and Labruna unpublished |
| -3.17 | -60 | Personal comm. | Molecular | Nava and Labruna unpublished |
| -2.03 | -60.02 | Personal comm. | Molecular | Nava and Labruna unpublished |
| -3.05 | -60.02 | Personal comm. | Molecular | Nava and Labruna unpublished |
| -3.1 | -60.02 | Personal comm. | Molecular | Nava and Labruna unpublished |
| -13.98 | -60.42 | Personal comm. | Molecular | Nava and Labruna unpublished |
| -2.82 | -60.67 | Personal comm. | Molecular | Nava and Labruna unpublished |
| 14.53 | -60.83 | Personal comm. | Molecular | Nava and Labruna unpublished |
| 14.47 | -60.88 | Personal comm. | Molecular | Nava and Labruna unpublished |
| 14.48 | -60.9 | Personal comm. | Molecular | Nava and Labruna unpublished |
| 14.58 | -60.9 | Personal comm. | Molecular | Nava and Labruna unpublished |
| 14.47 | -60.92 | Personal comm. | Molecular | Nava and Labruna unpublished |
| 14.67 | -60.95 | Personal comm. | Molecular | Nava and Labruna unpublished |
| 13.92 | -60.98 | Personal comm. | Molecular | Nava and Labruna unpublished |
| 14.52 | -61.03 | Personal comm. | Molecular | Nava and Labruna unpublished |
| 14.67 | -61.03 | Personal comm. | Molecular | Nava and Labruna unpublished |
| 14.6 | -61.07 | Personal comm. | Molecular | Nava and Labruna unpublished |
| 14.83 | -61.07 | Personal comm. | Molecular | Nava and Labruna unpublished |
| 14.62 | -61.1 | Personal comm. | Molecular | Nava and Labruna unpublished |
| 14.82 | -61.12 | Personal comm. | Molecular | Nava and Labruna unpublished |
| 10.68 | -61.17 | Personal comm. | Molecular | Nava and Labruna unpublished |
| 14.73 | -61.17 | Personal comm. | Molecular | Nava and Labruna unpublished |
| 13.25 | -61.18 | Personal comm. | Molecular | Nava and Labruna unpublished |
| 15.93 | -61.27 | Personal comm. | Molecular | Nava and Labruna unpublished |
| 16.25 | -61.27 | Personal comm. | Molecular | Nava and Labruna unpublished |
| 15.5 | -61.33 | Personal comm. | Molecular | Nava and Labruna unpublished |
| 16.33 | -61.33 | Personal comm. | Molecular | Nava and Labruna unpublished |
| 10.63 | -61.38 | Personal comm. | Molecular | Nava and Labruna unpublished |
| 16.23 | -61.38 | Personal comm. | Molecular | Nava and Labruna unpublished |
| 10.28 | -61.45 | Personal comm. | Molecular | Nava and Labruna unpublished |
| 10.65 | -61.5 | Personal comm. | Molecular | Nava and Labruna unpublished |
| 16.23 | -61.52 | Personal comm. | Molecular | Nava and Labruna unpublished |
| 16.42 | -61.52 | Personal comm. | Molecular | Nava and Labruna unpublished |
| 16.13 | -61.57 | Personal comm. | Molecular | Nava and Labruna unpublished |
| 16.27 | -61.62 | Personal comm. | Molecular | Nava and Labruna unpublished |
| 16.18 | -61.65 | Personal comm. | Molecular | Nava and Labruna unpublished |
| 16.02 | -61.73 | Personal comm. | Molecular | Nava and Labruna unpublished |
| 16.05 | -61.75 | Personal comm. | Molecular | Nava and Labruna unpublished |
| 17.08 | -61.8 | Personal comm. | Molecular | Nava and Labruna unpublished |
| -23.9 | -61.85 | Personal comm. | Molecular | Nava and Labruna unpublished |
| 17.1 | -61.85 | Personal comm. | Molecular | Nava and Labruna unpublished |
| 17.32 | -62.73 | Personal comm. | Molecular | Nava and Labruna unpublished |
| -10.28 | -62.87 | Personal comm. | Molecular | Nava and Labruna unpublished |
| 8.13 | -63.15 | Personal comm. | Molecular | Nava and Labruna unpublished |
| -17.78 | -63.2 | Personal comm. | Molecular | Nava and Labruna unpublished |
| -10.25 | -63.23 | Personal comm. | Molecular | Nava and Labruna unpublished |
| -10.3 | -63.23 | Personal comm. | Molecular | Nava and Labruna unpublished |
| 10.62 | -63.23 | Personal comm. | Molecular | Nava and Labruna unpublished |
| -10.12 | -63.3 | Personal comm. | Molecular | Nava and Labruna unpublished |
| -10.17 | -63.32 | Personal comm. | Molecular | Nava and Labruna unpublished |
| -10.25 | -63.32 | Personal comm. | Molecular | Nava and Labruna unpublished |
| -10.28 | -63.32 | Personal comm. | Molecular | Nava and Labruna unpublished |
| -10.43 | -63.35 | Personal comm. | Molecular | Nava and Labruna unpublished |
| -10.37 | -63.42 | Personal comm. | Molecular | Nava and Labruna unpublished |
| -10.63 | -63.48 | Personal comm. | Molecular | Nava and Labruna unpublished |
| -10.08 | -63.52 | Personal comm. | Molecular | Nava and Labruna unpublished |
| 8.12 | -63.55 | Personal comm. | Molecular | Nava and Labruna unpublished |
| -10.15 | -63.67 | Personal comm. | Molecular | Nava and Labruna unpublished |
| -8.77 | -63.9 | Personal comm. | Molecular | Nava and Labruna unpublished |
| -12.43 | -64.22 | Personal comm. | Molecular | Nava and Labruna unpublished |
| -12.62 | -64.22 | Personal comm. | Molecular | Nava and Labruna unpublished |
| 9.18 | -64.23 | Personal comm. | Molecular | Nava and Labruna unpublished |
| 9.45 | -64.27 | Personal comm. | Molecular | Nava and Labruna unpublished |
| 9.3 | -64.35 | Personal comm. | Molecular | Nava and Labruna unpublished |
| -12.42 | -64.42 | Personal comm. | Molecular | Nava and Labruna unpublished |
| 8.82 | -64.72 | Personal comm. | Molecular | Nava and Labruna unpublished |
| 4.2 | -64.93 | Personal comm. | Molecular | Nava and Labruna unpublished |
| 9.33 | -65.32 | Personal comm. | Molecular | Nava and Labruna unpublished |
| 18.45 | -66.07 | Personal comm. | Molecular | Nava and Labruna unpublished |
| 10.48 | -66.92 | Personal comm. | Molecular | Nava and Labruna unpublished |
| 18.2 | -67.13 | Personal comm. | Molecular | Nava and Labruna unpublished |
| 10.23 | -67.58 | Personal comm. | Molecular | Nava and Labruna unpublished |
| 12.15 | -68.27 | Personal comm. | Molecular | Nava and Labruna unpublished |
| -9.65 | -68.58 | Personal comm. | Molecular | Nava and Labruna unpublished |
| 12.18 | -69 | Personal comm. | Molecular | Nava and Labruna unpublished |
| 10.07 | -69.2 | Personal comm. | Molecular | Nava and Labruna unpublished |
| 9.52 | -69.27 | Personal comm. | Molecular | Nava and Labruna unpublished |
| 10.07 | -69.32 | Personal comm. | Molecular | Nava and Labruna unpublished |
| 10.12 | -69.52 | Personal comm. | Molecular | Nava and Labruna unpublished |
| 9.12 | -69.72 | Personal comm. | Molecular | Nava and Labruna unpublished |
| 18.47 | -69.88 | Personal comm. | Molecular | Nava and Labruna unpublished |
| 12.5 | -69.97 | Personal comm. | Molecular | Nava and Labruna unpublished |
| 6.13 | -70.67 | Personal comm. | Molecular | Nava and Labruna unpublished |
| 7.08 | -70.77 | Personal comm. | Molecular | Nava and Labruna unpublished |
| 8.63 | -71.65 | Personal comm. | Molecular | Nava and Labruna unpublished |
| 6.97 | -71.88 | Personal comm. | Molecular | Nava and Labruna unpublished |
| -13.48 | -71.98 | Personal comm. | Molecular | Nava and Labruna unpublished |
| 7.63 | -72.3 | Personal comm. | Molecular | Nava and Labruna unpublished |
| -3.12 | -73.03 | Personal comm. | Molecular | Nava and Labruna unpublished |
| 4.25 | -73.57 | Personal comm. | Molecular | Nava and Labruna unpublished |
| 5.15 | -73.88 | Personal comm. | Molecular | Nava and Labruna unpublished |
| 11.2 | -73.92 | Personal comm. | Molecular | Nava and Labruna unpublished |
| 4.62 | -74.08 | Personal comm. | Molecular | Nava and Labruna unpublished |
| 4.73 | -74.12 | Personal comm. | Molecular | Nava and Labruna unpublished |
| 6.47 | -74.42 | Personal comm. | Molecular | Nava and Labruna unpublished |
| 6.4 | -74.43 | Personal comm. | Molecular | Nava and Labruna unpublished |
| 5.1 | -74.45 | Personal comm. | Molecular | Nava and Labruna unpublished |
| 5.12 | -74.45 | Personal comm. | Molecular | Nava and Labruna unpublished |
| 5.02 | -74.47 | Personal comm. | Molecular | Nava and Labruna unpublished |
| 5.47 | -74.65 | Personal comm. | Molecular | Nava and Labruna unpublished |
| 5.47 | -74.7 | Personal comm. | Molecular | Nava and Labruna unpublished |
| 9.22 | -74.73 | Personal comm. | Molecular | Nava and Labruna unpublished |
| 6.55 | -74.78 | Personal comm. | Molecular | Nava and Labruna unpublished |
| 3.75 | -74.83 | Personal comm. | Molecular | Nava and Labruna unpublished |
| 4.32 | -74.83 | Personal comm. | Molecular | Nava and Labruna unpublished |
| 5.58 | -74.88 | Personal comm. | Molecular | Nava and Labruna unpublished |
| 5.32 | -74.92 | Personal comm. | Molecular | Nava and Labruna unpublished |
| 3.93 | -75.02 | Personal comm. | Molecular | Nava and Labruna unpublished |
| 6.47 | -75.02 | Personal comm. | Molecular | Nava and Labruna unpublished |
| 4.4 | -75.13 | Personal comm. | Molecular | Nava and Labruna unpublished |
| 8.67 | -75.13 | Personal comm. | Molecular | Nava and Labruna unpublished |
| -9.53 | -75.23 | Personal comm. | Molecular | Nava and Labruna unpublished |
| 9.32 | -75.28 | Personal comm. | Molecular | Nava and Labruna unpublished |
| 9.18 | -75.38 | Personal comm. | Molecular | Nava and Labruna unpublished |
| 9.3 | -75.38 | Personal comm. | Molecular | Nava and Labruna unpublished |
| 8.93 | -75.42 | Personal comm. | Molecular | Nava and Labruna unpublished |
| 9.18 | -75.42 | Personal comm. | Molecular | Nava and Labruna unpublished |
| 9.45 | -75.43 | Personal comm. | Molecular | Nava and Labruna unpublished |
| 5.07 | -75.48 | Personal comm. | Molecular | Nava and Labruna unpublished |
| 5.17 | -75.52 | Personal comm. | Molecular | Nava and Labruna unpublished |
| 6.23 | -75.57 | Personal comm. | Molecular | Nava and Labruna unpublished |
| 6.25 | -75.57 | Personal comm. | Molecular | Nava and Labruna unpublished |
| 8.4 | -75.57 | Personal comm. | Molecular | Nava and Labruna unpublished |
| 8.87 | -75.6 | Personal comm. | Molecular | Nava and Labruna unpublished |
| 8.8 | -75.7 | Personal comm. | Molecular | Nava and Labruna unpublished |
| 8.82 | -75.77 | Personal comm. | Molecular | Nava and Labruna unpublished |
| 8.82 | -75.83 | Personal comm. | Molecular | Nava and Labruna unpublished |
| 8.95 | -75.83 | Personal comm. | Molecular | Nava and Labruna unpublished |
| 8.75 | -75.88 | Personal comm. | Molecular | Nava and Labruna unpublished |
| 4.32 | -76.07 | Personal comm. | Molecular | Nava and Labruna unpublished |
| 4.42 | -76.1 | Personal comm. | Molecular | Nava and Labruna unpublished |
| -9.93 | -76.23 | Personal comm. | Molecular | Nava and Labruna unpublished |
| 3.53 | -76.3 | Personal comm. | Molecular | Nava and Labruna unpublished |
| 8.9 | -76.35 | Personal comm. | Molecular | Nava and Labruna unpublished |
| 3.43 | -76.43 | Personal comm. | Molecular | Nava and Labruna unpublished |
| 3.85 | -76.43 | Personal comm. | Molecular | Nava and Labruna unpublished |
| -11.85 | -76.45 | Personal comm. | Molecular | Nava and Labruna unpublished |
| 3.43 | -76.52 | Personal comm. | Molecular | Nava and Labruna unpublished |
| 8.13 | -76.55 | Personal comm. | Molecular | Nava and Labruna unpublished |
| 17.98 | -76.78 | Personal comm. | Molecular | Nava and Labruna unpublished |
| -12.05 | -77.12 | Personal comm. | Molecular | Nava and Labruna unpublished |
| 17.75 | -77.22 | Personal comm. | Molecular | Nava and Labruna unpublished |
| 17.88 | -77.3 | Personal comm. | Molecular | Nava and Labruna unpublished |
| 25.05 | -77.35 | Personal comm. | Molecular | Nava and Labruna unpublished |
| -8.67 | -77.37 | Personal comm. | Molecular | Nava and Labruna unpublished |
| -8.67 | -77.42 | Personal comm. | Molecular | Nava and Labruna unpublished |
| -8.67 | -77.45 | Personal comm. | Molecular | Nava and Labruna unpublished |
| 18.27 | -77.93 | Personal comm. | Molecular | Nava and Labruna unpublished |
| 8.5 | -77.98 | Personal comm. | Molecular | Nava and Labruna unpublished |
| 21.25 | -78.13 | Personal comm. | Molecular | Nava and Labruna unpublished |
| 8.65 | -78.15 | Personal comm. | Molecular | Nava and Labruna unpublished |
| -5.78 | -78.23 | Personal comm. | Molecular | Nava and Labruna unpublished |
| -5.95 | -78.23 | Personal comm. | Molecular | Nava and Labruna unpublished |
| 8.67 | -78.35 | Personal comm. | Molecular | Nava and Labruna unpublished |
| -9.08 | -78.48 | Personal comm. | Molecular | Nava and Labruna unpublished |
| 21.68 | -78.63 | Personal comm. | Molecular | Nava and Labruna unpublished |
| -5.08 | -79 | Personal comm. | Molecular | Nava and Labruna unpublished |
| 22.3 | -79.1 | Personal comm. | Molecular | Nava and Labruna unpublished |
| 9.17 | -79.1 | Personal comm. | Molecular | Nava and Labruna unpublished |
| 9.28 | -79.17 | Personal comm. | Molecular | Nava and Labruna unpublished |
| 8.98 | -79.52 | Personal comm. | Molecular | Nava and Labruna unpublished |
| 8.95 | -79.53 | Personal comm. | Molecular | Nava and Labruna unpublished |
| 8.95 | -79.65 | Personal comm. | Molecular | Nava and Labruna unpublished |
| 9.07 | -79.65 | Personal comm. | Molecular | Nava and Labruna unpublished |
| 9.18 | -79.65 | Personal comm. | Molecular | Nava and Labruna unpublished |
| 9.12 | -79.68 | Personal comm. | Molecular | Nava and Labruna unpublished |
| -5.52 | -79.72 | Personal comm. | Molecular | Nava and Labruna unpublished |
| 8.88 | -79.78 | Personal comm. | Molecular | Nava and Labruna unpublished |
| -6.77 | -79.83 | Personal comm. | Molecular | Nava and Labruna unpublished |
| 8.92 | -79.88 | Personal comm. | Molecular | Nava and Labruna unpublished |
| 9.1 | -79.88 | Personal comm. | Molecular | Nava and Labruna unpublished |
| 9.35 | -79.88 | Personal comm. | Molecular | Nava and Labruna unpublished |
| 9.37 | -79.9 | Personal comm. | Molecular | Nava and Labruna unpublished |
| 22.4 | -79.95 | Personal comm. | Molecular | Nava and Labruna unpublished |
| 7.53 | -80.03 | Personal comm. | Molecular | Nava and Labruna unpublished |
| -5.1 | -80.07 | Personal comm. | Molecular | Nava and Labruna unpublished |
| 8.43 | -80.07 | Personal comm. | Molecular | Nava and Labruna unpublished |
| 8.6 | -80.12 | Personal comm. | Molecular | Nava and Labruna unpublished |
| 7.63 | -80.17 | Personal comm. | Molecular | Nava and Labruna unpublished |
| 8.4 | -80.25 | Personal comm. | Molecular | Nava and Labruna unpublished |
| 8.5 | -80.35 | Personal comm. | Molecular | Nava and Labruna unpublished |
| 7.97 | -80.43 | Personal comm. | Molecular | Nava and Labruna unpublished |
| 7.98 | -80.43 | Personal comm. | Molecular | Nava and Labruna unpublished |
| -4.68 | -80.47 | Personal comm. | Molecular | Nava and Labruna unpublished |
| 8.32 | -80.5 | Personal comm. | Molecular | Nava and Labruna unpublished |
| 8.2 | -80.58 | Personal comm. | Molecular | Nava and Labruna unpublished |
| 7.9 | -80.62 | Personal comm. | Molecular | Nava and Labruna unpublished |
| 8.07 | -80.62 | Personal comm. | Molecular | Nava and Labruna unpublished |
| 7.95 | -80.78 | Personal comm. | Molecular | Nava and Labruna unpublished |
| 7.75 | -80.87 | Personal comm. | Molecular | Nava and Labruna unpublished |
| 8 | -81.05 | Personal comm. | Molecular | Nava and Labruna unpublished |
| 8.52 | -81.08 | Personal comm. | Molecular | Nava and Labruna unpublished |
| 8.15 | -81.18 | Personal comm. | Molecular | Nava and Labruna unpublished |
| 8.32 | -81.22 | Personal comm. | Molecular | Nava and Labruna unpublished |
| 8.22 | -81.27 | Personal comm. | Molecular | Nava and Labruna unpublished |
| 8.13 | -81.45 | Personal comm. | Molecular | Nava and Labruna unpublished |
| 22.35 | -81.5 | Personal comm. | Molecular | Nava and Labruna unpublished |
| 22.97 | -82.15 | Personal comm. | Molecular | Nava and Labruna unpublished |
| 23.03 | -82.27 | Personal comm. | Molecular | Nava and Labruna unpublished |
| 22.93 | -82.38 | Personal comm. | Molecular | Nava and Labruna unpublished |
| 22.97 | -82.38 | Personal comm. | Molecular | Nava and Labruna unpublished |
| 9.28 | -82.38 | Personal comm. | Molecular | Nava and Labruna unpublished |
| 23.13 | -82.4 | Personal comm. | Molecular | Nava and Labruna unpublished |
| 23 | -82.42 | Personal comm. | Molecular | Nava and Labruna unpublished |
| 8.43 | -82.43 | Personal comm. | Molecular | Nava and Labruna unpublished |
| 8.73 | -82.43 | Personal comm. | Molecular | Nava and Labruna unpublished |
| 8.62 | -82.45 | Personal comm. | Molecular | Nava and Labruna unpublished |
| 8.37 | -82.48 | Personal comm. | Molecular | Nava and Labruna unpublished |
| 8.68 | -82.48 | Personal comm. | Molecular | Nava and Labruna unpublished |
| 9.43 | -82.52 | Personal comm. | Molecular | Nava and Labruna unpublished |
| 8.4 | -82.55 | Personal comm. | Molecular | Nava and Labruna unpublished |
| 8.5 | -82.57 | Personal comm. | Molecular | Nava and Labruna unpublished |
| 8.52 | -82.62 | Personal comm. | Molecular | Nava and Labruna unpublished |
| 9.6 | -82.78 | Personal comm. | Molecular | Nava and Labruna unpublished |
| 8.28 | -82.87 | Personal comm. | Molecular | Nava and Labruna unpublished |
| 22.73 | -82.95 | Personal comm. | Molecular | Nava and Labruna unpublished |
| 8.65 | -82.95 | Personal comm. | Molecular | Nava and Labruna unpublished |
| 9.9 | -83.68 | Personal comm. | Molecular | Nava and Labruna unpublished |
| 10.2 | -83.73 | Personal comm. | Molecular | Nava and Labruna unpublished |
| 22.25 | -83.83 | Personal comm. | Molecular | Nava and Labruna unpublished |
| 9.83 | -83.92 | Personal comm. | Molecular | Nava and Labruna unpublished |
| 9.92 | -83.98 | Personal comm. | Molecular | Nava and Labruna unpublished |
| 10.1 | -84 | Personal comm. | Molecular | Nava and Labruna unpublished |
| 9.37 | -84 | Personal comm. | Molecular | Nava and Labruna unpublished |
| 10.48 | -84.02 | Personal comm. | Molecular | Nava and Labruna unpublished |
| 9.85 | -84.03 | Personal comm. | Molecular | Nava and Labruna unpublished |
| 9.92 | -84.03 | Personal comm. | Molecular | Nava and Labruna unpublished |
| 9.87 | -84.07 | Personal comm. | Molecular | Nava and Labruna unpublished |
| 9.88 | -84.07 | Personal comm. | Molecular | Nava and Labruna unpublished |
| 9.93 | -84.08 | Personal comm. | Molecular | Nava and Labruna unpublished |
| 9.97 | -84.08 | Personal comm. | Molecular | Nava and Labruna unpublished |
| 10.38 | -84.13 | Personal comm. | Molecular | Nava and Labruna unpublished |
| 9.92 | -84.13 | Personal comm. | Molecular | Nava and Labruna unpublished |
| 9.93 | -84.17 | Personal comm. | Molecular | Nava and Labruna unpublished |
| 10.02 | -84.2 | Personal comm. | Molecular | Nava and Labruna unpublished |
| 22.12 | -84.27 | Personal comm. | Molecular | Nava and Labruna unpublished |
| 9.75 | -84.42 | Personal comm. | Molecular | Nava and Labruna unpublished |
| 9.98 | -84.75 | Personal comm. | Molecular | Nava and Labruna unpublished |
| 10.5 | -85.25 | Personal comm. | Molecular | Nava and Labruna unpublished |
| 12.07 | -85.37 | Personal comm. | Molecular | Nava and Labruna unpublished |
| 9.88 | -85.4 | Personal comm. | Molecular | Nava and Labruna unpublished |
| 10.05 | -85.42 | Personal comm. | Molecular | Nava and Labruna unpublished |
| 10.63 | -85.43 | Personal comm. | Molecular | Nava and Labruna unpublished |
| 11.43 | -85.82 | Personal comm. | Molecular | Nava and Labruna unpublished |
| 11.23 | -85.85 | Personal comm. | Molecular | Nava and Labruna unpublished |
| 13.08 | -86 | Personal comm. | Molecular | Nava and Labruna unpublished |
| 11.97 | -86.1 | Personal comm. | Molecular | Nava and Labruna unpublished |
| 12.72 | -86.12 | Personal comm. | Molecular | Nava and Labruna unpublished |
| 12.13 | -86.25 | Personal comm. | Molecular | Nava and Labruna unpublished |
| 21.17 | -86.85 | Personal comm. | Molecular | Nava and Labruna unpublished |
| 12.42 | -86.88 | Personal comm. | Molecular | Nava and Labruna unpublished |
| 20.5 | -86.95 | Personal comm. | Molecular | Nava and Labruna unpublished |
| 12.63 | -87.13 | Personal comm. | Molecular | Nava and Labruna unpublished |
| 12.65 | -87.17 | Personal comm. | Molecular | Nava and Labruna unpublished |
| 12.53 | -87.23 | Personal comm. | Molecular | Nava and Labruna unpublished |
| 20.55 | -87.93 | Personal comm. | Molecular | Nava and Labruna unpublished |
| 21.2 | -87.93 | Personal comm. | Molecular | Nava and Labruna unpublished |
| 19.68 | -88 | Personal comm. | Molecular | Nava and Labruna unpublished |
| 21.15 | -88.15 | Personal comm. | Molecular | Nava and Labruna unpublished |
| 21.02 | -88.18 | Personal comm. | Molecular | Nava and Labruna unpublished |
| 20.68 | -88.2 | Personal comm. | Molecular | Nava and Labruna unpublished |
| 21.3 | -88.27 | Personal comm. | Molecular | Nava and Labruna unpublished |
| 18.22 | -88.32 | Personal comm. | Molecular | Nava and Labruna unpublished |
| 21.37 | -88.42 | Personal comm. | Molecular | Nava and Labruna unpublished |
| 15.72 | -88.58 | Personal comm. | Molecular | Nava and Labruna unpublished |
| 21.2 | -88.63 | Personal comm. | Molecular | Nava and Labruna unpublished |
| 17.77 | -88.8 | Personal comm. | Molecular | Nava and Labruna unpublished |
| 15.47 | -88.83 | Personal comm. | Molecular | Nava and Labruna unpublished |
| 17 | -88.9 | Personal comm. | Molecular | Nava and Labruna unpublished |
| 20.13 | -88.92 | Personal comm. | Molecular | Nava and Labruna unpublished |
| 20.93 | -89 | Personal comm. | Molecular | Nava and Labruna unpublished |
| 20.07 | -89.05 | Personal comm. | Molecular | Nava and Labruna unpublished |
| 20.23 | -89.1 | Personal comm. | Molecular | Nava and Labruna unpublished |
| 20.47 | -89.2 | Personal comm. | Molecular | Nava and Labruna unpublished |
| 21.1 | -89.28 | Personal comm. | Molecular | Nava and Labruna unpublished |
| 20.4 | -89.53 | Personal comm. | Molecular | Nava and Labruna unpublished |
| 15.07 | -89.57 | Personal comm. | Molecular | Nava and Labruna unpublished |
| 20.97 | -89.62 | Personal comm. | Molecular | Nava and Labruna unpublished |
| 20.85 | -89.63 | Personal comm. | Molecular | Nava and Labruna unpublished |
| 21.27 | -89.65 | Personal comm. | Molecular | Nava and Labruna unpublished |
| 21.1 | -89.67 | Personal comm. | Molecular | Nava and Labruna unpublished |
| 16.77 | -89.7 | Personal comm. | Molecular | Nava and Labruna unpublished |
| 20.48 | -89.72 | Personal comm. | Molecular | Nava and Labruna unpublished |
| 20.88 | -89.75 | Personal comm. | Molecular | Nava and Labruna unpublished |
| 20.73 | -89.82 | Personal comm. | Molecular | Nava and Labruna unpublished |
| 20.55 | -89.85 | Personal comm. | Molecular | Nava and Labruna unpublished |
| 21.02 | -89.87 | Personal comm. | Molecular | Nava and Labruna unpublished |
| 20.57 | -90 | Personal comm. | Molecular | Nava and Labruna unpublished |
| 14.13 | -90.02 | Personal comm. | Molecular | Nava and Labruna unpublished |
| -0.73 | -90.28 | Personal comm. | Molecular | Nava and Labruna unpublished |
| -0.73 | -90.32 | Personal comm. | Molecular | Nava and Labruna unpublished |
| 15.47 | -90.38 | Personal comm. | Molecular | Nava and Labruna unpublished |
| 19.8 | -90.43 | Personal comm. | Molecular | Nava and Labruna unpublished |
| 14.28 | -90.78 | Personal comm. | Molecular | Nava and Labruna unpublished |
| 14.2 | -90.85 | Personal comm. | Molecular | Nava and Labruna unpublished |
| 14.08 | -91.13 | Personal comm. | Molecular | Nava and Labruna unpublished |
| 15.65 | -91.43 | Personal comm. | Molecular | Nava and Labruna unpublished |
| 14.48 | -91.52 | Personal comm. | Molecular | Nava and Labruna unpublished |
| 14.9 | -92.27 | Personal comm. | Molecular | Nava and Labruna unpublished |
| 14.87 | -92.45 | Personal comm. | Molecular | Nava and Labruna unpublished |
| 15.13 | -92.47 | Personal comm. | Molecular | Nava and Labruna unpublished |
| 15.28 | -92.68 | Personal comm. | Molecular | Nava and Labruna unpublished |
| 18.02 | -92.87 | Personal comm. | Molecular | Nava and Labruna unpublished |
| 15.42 | -92.9 | Personal comm. | Molecular | Nava and Labruna unpublished |
| 16.08 | -93.73 | Personal comm. | Molecular | Nava and Labruna unpublished |
| 16.57 | -95.1 | Personal comm. | Molecular | Nava and Labruna unpublished |
| 16.17 | -95.18 | Personal comm. | Molecular | Nava and Labruna unpublished |
| 16.32 | -95.23 | Personal comm. | Molecular | Nava and Labruna unpublished |
| 18.37 | -95.75 | Personal comm. | Molecular | Nava and Labruna unpublished |
| 18.35 | -95.8 | Personal comm. | Molecular | Nava and Labruna unpublished |
| 19.18 | -96.13 | Personal comm. | Molecular | Nava and Labruna unpublished |
| 16.1 | -97.15 | Personal comm. | Molecular | Nava and Labruna unpublished |
| 22.22 | -97.85 | Personal comm. | Molecular | Nava and Labruna unpublished |
| 21.25 | -98.78 | Personal comm. | Molecular | Nava and Labruna unpublished |
| 20.57 | -98.87 | Personal comm. | Molecular | Nava and Labruna unpublished |
| 26.18 | -98.92 | Personal comm. | Molecular | Nava and Labruna unpublished |
| 21.37 | -98.98 | Personal comm. | Molecular | Nava and Labruna unpublished |
| 21.98 | -99 | Personal comm. | Molecular | Nava and Labruna unpublished |
| 19.4 | -99.12 | Personal comm. | Molecular | Nava and Labruna unpublished |
| 23.73 | -99.13 | Personal comm. | Molecular | Nava and Labruna unpublished |
| 18.9 | -99.15 | Personal comm. | Molecular | Nava and Labruna unpublished |
| 18.92 | -99.22 | Personal comm. | Molecular | Nava and Labruna unpublished |
| 18.82 | -99.23 | Personal comm. | Molecular | Nava and Labruna unpublished |
| 18.78 | -99.28 | Personal comm. | Molecular | Nava and Labruna unpublished |
| 17.63 | -99.53 | Personal comm. | Molecular | Nava and Labruna unpublished |
| 24.85 | -99.55 | Personal comm. | Molecular | Nava and Labruna unpublished |
| 18.23 | -99.63 | Personal comm. | Molecular | Nava and Labruna unpublished |
| 25.18 | -99.83 | Personal comm. | Molecular | Nava and Labruna unpublished |
| 25.17 | -99.87 | Personal comm. | Molecular | Nava and Labruna unpublished |
| 22.740567 | -82.959828 | Literature | Morphological or unknown | [7] |
| 21.15 | -88.066667 | Literature | Morphological or unknown | [8] |
| 26.824934 | -101.917715 | Literature | Morphological or unknown | [9] |
| 25.672919 | -102.842982 | Literature | Morphological or unknown | [9] |
| 25.425481 | -102.899175 | Literature | Morphological or unknown | [9] |
| 25.489964 | -102.943727 | Literature | Morphological or unknown | [9] |
| 25.698845 | -102.950456 | Literature | Morphological or unknown | [9] |
| 25.761716 | -102.989931 | Literature | Morphological or unknown | [9] |
| 25.4834 | -103.06458 | Literature | Morphological or unknown | [9] |
| 25.780656 | -103.115702 | Literature | Morphological or unknown | [9] |
| 25.828044 | -103.130185 | Literature | Morphological or unknown | [9] |
| 25.489941 | -103.139593 | Literature | Morphological or unknown | [9] |
| 25.788946 | -103.193632 | Literature | Morphological or unknown | [9] |
| 25.928244 | -103.201224 | Literature | Morphological or unknown | [9] |
| 25.611035 | -103.21355 | Literature | Morphological or unknown | [9] |
| 25.627147 | -103.223596 | Literature | Morphological or unknown | [9] |
| 25.765418 | -103.240132 | Literature | Morphological or unknown | [9] |
| 25.747549 | -103.266058 | Literature | Morphological or unknown | [9] |
| 25.640659 | -103.267676 | Literature | Morphological or unknown | [9] |
| 25.847056 | -103.278677 | Literature | Morphological or unknown | [9] |
| 25.589898 | -103.301185 | Literature | Morphological or unknown | [9] |
| 25.410218 | -103.304672 | Literature | Morphological or unknown | [9] |
| 25.640693 | -103.327909 | Literature | Morphological or unknown | [9] |
| 25.5028 | -103.328995 | Literature | Morphological or unknown | [9] |
| 25.625995 | -103.39467 | Literature | Morphological or unknown | [9] |
| 25.566903 | -103.430672 | Literature | Morphological or unknown | [9] |
| 25.287589 | -103.500073 | Literature | Morphological or unknown | [9] |
| 27.787336 | -99.875066 | Literature | Morphological or unknown | [9] |
| 20.302778 | -89.418333 | Literature | Morphological or unknown | [10] |
| 20.468056 | -89.213889 | Literature | Morphological or unknown | [11] |
| -17.163611 | 145.547222 | BISON | Morphological or unknown | Queensland Museum |
| -23.8605 | 148.1577 | BISON | Morphological or unknown | Queensland Museum |
| 26.355471 | 127.766419 | Literature | Morphological or unknown | [12] |
| 26.458664 | 127.918469 | Literature | Morphological or unknown | [12] |
| 7.415354 | 100.171987 | Literature | Morphological or unknown | [13] |
| 14.03333 | -0.03333 | VectorMap | Morphological or unknown | [14–16] |
| 7.76666 | -0.05 | VectorMap | Morphological or unknown | [14–16] |
| 11.8 | -0.56666 | VectorMap | Morphological or unknown | [14–16] |
| 11.16666 | -1.15 | VectorMap | Morphological or unknown | [14–16] |
| 12.36666 | -1.51666 | VectorMap | Morphological or unknown | [14–16] |
| 6.2 | -1.66666 | VectorMap | Morphological or unknown | [14–16] |
| 9.21666 | -1.85 | VectorMap | Morphological or unknown | [14–16] |
| 9.58333 | -11.55 | VectorMap | Morphological or unknown | [14–16] |
| 8.3 | -11.83333 | VectorMap | Morphological or unknown | [14–16] |
| 12.38333 | -12.18333 | VectorMap | Morphological or unknown | [14–16] |
| 9.5 | -12.23333 | VectorMap | Morphological or unknown | [14–16] |
| 14.9 | -12.45 | VectorMap | Morphological or unknown | [14–16] |
| 17.63333 | -12.66666 | VectorMap | Morphological or unknown | [14–16] |
| 13.06666 | -12.71666 | VectorMap | Morphological or unknown | [14–16] |
| 22.68333 | -12.71666 | VectorMap | Morphological or unknown | [14–16] |
| 8.36666 | -12.93333 | VectorMap | Morphological or unknown | [14–16] |
| 9.95 | -12.93333 | VectorMap | Morphological or unknown | [14–16] |
| 10.9 | -13.03333 | VectorMap | Morphological or unknown | [14–16] |
| 20.51666 | -13.05 | VectorMap | Morphological or unknown | [14–16] |
| 16.15 | -13.5 | VectorMap | Morphological or unknown | [14–16] |
| 13.78333 | -13.66666 | VectorMap | Morphological or unknown | [14–16] |
| 16.41666 | -14.03333 | VectorMap | Morphological or unknown | [14–16] |
| 16.7 | -14.03333 | VectorMap | Morphological or unknown | [14–16] |
| 16.58333 | -14.26666 | VectorMap | Morphological or unknown | [14–16] |
| 15.35 | -15.48333 | VectorMap | Morphological or unknown | [14–16] |
| 16.51666 | -15.5 | VectorMap | Morphological or unknown | [14–16] |
| 12.65 | -15.68333 | VectorMap | Morphological or unknown | [14–16] |
| 15.61666 | -16.21666 | VectorMap | Morphological or unknown | [14–16] |
| 12.81666 | -16.23333 | VectorMap | Morphological or unknown | [14–16] |
| 12.58333 | -16.26666 | VectorMap | Morphological or unknown | [14–16] |
| 13.4 | -16.45555 | VectorMap | Morphological or unknown | [14–16] |
| 14.5 | -16.51666 | VectorMap | Morphological or unknown | [14–16] |
| 14.16666 | -16.83333 | VectorMap | Morphological or unknown | [14–16] |
| 14.8 | -16.93333 | VectorMap | Morphological or unknown | [14–16] |
| 14.75 | -17.13333 | VectorMap | Morphological or unknown | [14–16] |
| 15.21666 | -2.36666 | VectorMap | Morphological or unknown | [14–16] |
| 9.16666 | -2.75 | VectorMap | Morphological or unknown | [14–16] |
| 9.93333 | -2.81666 | VectorMap | Morphological or unknown | [14–16] |
| 10.33333 | -2.91666 | VectorMap | Morphological or unknown | [14–16] |
| 8.23333 | -2.96666 | VectorMap | Morphological or unknown | [14–16] |
| 9.26666 | -3 | VectorMap | Morphological or unknown | [14–16] |
| 16.76666 | -3.01666 | VectorMap | Morphological or unknown | [14–16] |
| 8.35 | -3.16666 | VectorMap | Morphological or unknown | [14–16] |
| 10.33333 | -3.18333 | VectorMap | Morphological or unknown | [14–16] |
| 8.68333 | -3.2 | VectorMap | Morphological or unknown | [14–16] |
| 12.9 | -3.4 | VectorMap | Morphological or unknown | [14–16] |
| 13.16666 | -3.41666 | VectorMap | Morphological or unknown | [14–16] |
| 13.4 | -3.46666 | VectorMap | Morphological or unknown | [14–16] |
| 6.73333 | -3.48333 | VectorMap | Morphological or unknown | [14–16] |
| 8.5 | -3.55 | VectorMap | Morphological or unknown | [14–16] |
| 15.06666 | -3.56666 | VectorMap | Morphological or unknown | [14–16] |
| 11.68333 | -3.63333 | VectorMap | Morphological or unknown | [14–16] |
| 11.21666 | -3.76666 | VectorMap | Morphological or unknown | [14–16] |
| 6.03333 | -3.81666 | VectorMap | Morphological or unknown | [14–16] |
| 6.48333 | -3.85 | VectorMap | Morphological or unknown | [14–16] |
| 12.73333 | -3.86666 | VectorMap | Morphological or unknown | [14–16] |
| 15.93333 | -4 | VectorMap | Morphological or unknown | [14–16] |
| 5.41666 | -4.05 | VectorMap | Morphological or unknown | [14–16] |
| 9.56666 | -4.05 | VectorMap | Morphological or unknown | [14–16] |
| 6.06666 | -4.08333 | VectorMap | Morphological or unknown | [14–16] |
| 5.8 | -4.13333 | VectorMap | Morphological or unknown | [14–16] |
| 11.15 | -4.15 | VectorMap | Morphological or unknown | [14–16] |
| 5.31666 | -4.25 | VectorMap | Morphological or unknown | [14–16] |
| 11.31666 | -4.33333 | VectorMap | Morphological or unknown | [14–16] |
| 5.7 | -4.43333 | VectorMap | Morphological or unknown | [14–16] |
| 11.46666 | -4.46666 | VectorMap | Morphological or unknown | [14–16] |
| 5.41666 | -4.51666 | VectorMap | Morphological or unknown | [14–16] |
| 10.63333 | -4.55 | VectorMap | Morphological or unknown | [14–16] |
| 13.9 | -4.55 | VectorMap | Morphological or unknown | [14–16] |
| 10.83333 | -4.61666 | VectorMap | Morphological or unknown | [14–16] |
| 9.15 | -4.61666 | VectorMap | Morphological or unknown | [14–16] |
| 10.66666 | -4.81666 | VectorMap | Morphological or unknown | [14–16] |
| 14.46666 | -4.91666 | VectorMap | Morphological or unknown | [14–16] |
| 7.75 | -5 | VectorMap | Morphological or unknown | [14–16] |
| 7.33333 | -5.1 | VectorMap | Morphological or unknown | [14–16] |
| 8.13333 | -5.1 | VectorMap | Morphological or unknown | [14–16] |
| 7.13333 | -5.18333 | VectorMap | Morphological or unknown | [14–16] |
| 6.81666 | -5.28333 | VectorMap | Morphological or unknown | [14–16] |
| 7.45 | -5.3 | VectorMap | Morphological or unknown | [14–16] |
| 7.66666 | -5.3 | VectorMap | Morphological or unknown | [14–16] |
| 5.83333 | -5.36666 | VectorMap | Morphological or unknown | [14–16] |
| 7.21666 | -5.36666 | VectorMap | Morphological or unknown | [14–16] |
| 6.38333 | -5.41666 | VectorMap | Morphological or unknown | [14–16] |
| 8.1 | -5.43333 | VectorMap | Morphological or unknown | [14–16] |
| 5.88333 | -5.61666 | VectorMap | Morphological or unknown | [14–16] |
| 7.88333 | -5.63333 | VectorMap | Morphological or unknown | [14–16] |
| 11.31666 | -5.66666 | VectorMap | Morphological or unknown | [14–16] |
| 9.4 | -5.71666 | VectorMap | Morphological or unknown | [14–16] |
| 6.61666 | -5.91666 | VectorMap | Morphological or unknown | [14–16] |
| 6.13333 | -5.93333 | VectorMap | Morphological or unknown | [14–16] |
| 14.25 | -6 | VectorMap | Morphological or unknown | [14–16] |
| 5.35 | -6.05 | VectorMap | Morphological or unknown | [14–16] |
| 13.45 | -6.26666 | VectorMap | Morphological or unknown | [14–16] |
| 5.65 | -6.63333 | VectorMap | Morphological or unknown | [14–16] |
| 7.5 | -7.26666 | VectorMap | Morphological or unknown | [14–16] |
| 5.26666 | -7.31666 | VectorMap | Morphological or unknown | [14–16] |
| 5.63333 | -7.35 | VectorMap | Morphological or unknown | [14–16] |
| 6.75 | -7.35 | VectorMap | Morphological or unknown | [14–16] |
| 5.86666 | -7.45 | VectorMap | Morphological or unknown | [14–16] |
| 7.41666 | -7.48333 | VectorMap | Morphological or unknown | [14–16] |
| 6.53333 | -7.58333 | VectorMap | Morphological or unknown | [14–16] |
| 7.33333 | -7.66666 | VectorMap | Morphological or unknown | [14–16] |
| 6.55 | -7.88333 | VectorMap | Morphological or unknown | [14–16] |
| 12.6 | -8.03333 | VectorMap | Morphological or unknown | [14–16] |
| 7.28333 | -8.05 | VectorMap | Morphological or unknown | [14–16] |
| 11.18333 | -8.15 | VectorMap | Morphological or unknown | [14–16] |
| 27.7 | -8.15 | VectorMap | Morphological or unknown | [14–16] |
| 6.86666 | -8.26666 | VectorMap | Morphological or unknown | [14–16] |
| 6.58333 | -8.41666 | VectorMap | Morphological or unknown | [14–16] |
| 8.05 | -8.56666 | VectorMap | Morphological or unknown | [14–16] |
| 30.48333 | -8.86666 | VectorMap | Morphological or unknown | [14–16] |
| 10.38333 | -9.3 | VectorMap | Morphological or unknown | [14–16] |
| 28.61666 | -9.41666 | VectorMap | Morphological or unknown | [14–16] |
| 15.23333 | -9.58333 | VectorMap | Morphological or unknown | [14–16] |
| 16.66666 | -9.61666 | VectorMap | Morphological or unknown | [14–16] |
| 15.4 | -9.71666 | VectorMap | Morphological or unknown | [14–16] |
| 28.48333 | -9.86666 | VectorMap | Morphological or unknown | [14–16] |
| 15.11666 | -9.9 | VectorMap | Morphological or unknown | [14–16] |
| 12.06666 | 0.35 | VectorMap | Morphological or unknown | [14–16] |
| 10.75 | 0.4 | VectorMap | Morphological or unknown | [14–16] |
| 14.73333 | 0.91666 | VectorMap | Morphological or unknown | [14–16] |
| 10.21666 | 1.35 | VectorMap | Morphological or unknown | [14–16] |
| 10.5 | 1.38333 | VectorMap | Morphological or unknown | [14–16] |
| 14.21666 | 1.45 | VectorMap | Morphological or unknown | [14–16] |
| 6.23333 | 1.48333 | VectorMap | Morphological or unknown | [14–16] |
| 9.66666 | 1.63333 | VectorMap | Morphological or unknown | [14–16] |
| 13.66666 | 1.78333 | VectorMap | Morphological or unknown | [14–16] |
| 7.73333 | 1.78333 | VectorMap | Morphological or unknown | [14–16] |
| 7.93333 | 1.96666 | VectorMap | Morphological or unknown | [14–16] |
| 5.45 | 10.06666 | VectorMap | Morphological or unknown | [14–16] |
| 3.8 | 10.13333 | VectorMap | Morphological or unknown | [14–16] |
| 5.71666 | 10.91666 | VectorMap | Morphological or unknown | [14–16] |
| 4.08333 | 11.2 | VectorMap | Morphological or unknown | [14–16] |
| 4.75 | 11.23333 | VectorMap | Morphological or unknown | [14–16] |
| 32.01666 | 11.36666 | VectorMap | Morphological or unknown | [14–16] |
| 4.35 | 11.41666 | VectorMap | Morphological or unknown | [14–16] |
| 3.51666 | 11.5 | VectorMap | Morphological or unknown | [14–16] |
| -5.85 | 12.28333 | VectorMap | Morphological or unknown | [14–16] |
| 32.88333 | 13.35 | VectorMap | Morphological or unknown | [14–16] |
| -15.31666 | 13.53333 | VectorMap | Morphological or unknown | [14–16] |
| -5.81666 | 13.83333 | VectorMap | Morphological or unknown | [14–16] |
| 31.76666 | 13.98333 | VectorMap | Morphological or unknown | [14–16] |
| -10.18333 | 14.13333 | VectorMap | Morphological or unknown | [14–16] |
| 11.05 | 14.15 | VectorMap | Morphological or unknown | [14–16] |
| 10.6 | 14.33333 | VectorMap | Morphological or unknown | [14–16] |
| -14.81666 | 14.55 | VectorMap | Morphological or unknown | [14–16] |
| 12.83333 | 14.58333 | VectorMap | Morphological or unknown | [14–16] |
| -9.53333 | 14.66666 | VectorMap | Morphological or unknown | [14–16] |
| 5.83333 | 14.83333 | VectorMap | Morphological or unknown | [14–16] |
| 12.11666 | 15.05 | VectorMap | Morphological or unknown | [14–16] |
| 32.38333 | 15.1 | VectorMap | Morphological or unknown | [14–16] |
| 32.01666 | 15.11666 | VectorMap | Morphological or unknown | [14–16] |
| -4.41666 | 15.23333 | VectorMap | Morphological or unknown | [14–16] |
| 5.96666 | 15.63333 | VectorMap | Morphological or unknown | [14–16] |
| -7.31666 | 16 | VectorMap | Morphological or unknown | [14–16] |
| -20.11666 | 16.15 | VectorMap | Morphological or unknown | [14–16] |
| -18.83333 | 16.33333 | VectorMap | Morphological or unknown | [14–16] |
| 31.21666 | 16.58333 | VectorMap | Morphological or unknown | [14–16] |
| -3.31666 | 17.36666 | VectorMap | Morphological or unknown | [14–16] |
| 4.18333 | 18.06666 | VectorMap | Morphological or unknown | [14–16] |
| -19.56666 | 18.11666 | VectorMap | Morphological or unknown | [14–16] |
| 13.21666 | 18.33333 | VectorMap | Morphological or unknown | [14–16] |
| 13.08333 | 18.6 | VectorMap | Morphological or unknown | [14–16] |
| -0.11666 | 18.66666 | VectorMap | Morphological or unknown | [14–16] |
| 2.35 | 19.05 | VectorMap | Morphological or unknown | [14–16] |
| 5.71666 | 19.06666 | VectorMap | Morphological or unknown | [14–16] |
| 6.98333 | 19.16666 | VectorMap | Morphological or unknown | [14–16] |
| 7.35 | 2.05 | VectorMap | Morphological or unknown | [14–16] |
| 7.56666 | 2.05 | VectorMap | Morphological or unknown | [14–16] |
| 7.75 | 2.18333 | VectorMap | Morphological or unknown | [14–16] |
| 9.2 | 2.26666 | VectorMap | Morphological or unknown | [14–16] |
| 8.8 | 2.31666 | VectorMap | Morphological or unknown | [14–16] |
| 11.16666 | 2.41666 | VectorMap | Morphological or unknown | [14–16] |
| 6.35 | 2.43333 | VectorMap | Morphological or unknown | [14–16] |
| 9.75 | 2.43333 | VectorMap | Morphological or unknown | [14–16] |
| 7.66666 | 2.48333 | VectorMap | Morphological or unknown | [14–16] |
| 8.03333 | 2.48333 | VectorMap | Morphological or unknown | [14–16] |
| 8.68333 | 2.6 | VectorMap | Morphological or unknown | [14–16] |
| 9.35 | 2.61666 | VectorMap | Morphological or unknown | [14–16] |
| 6.43333 | 2.65 | VectorMap | Morphological or unknown | [14–16] |
| 6.96666 | 2.68333 | VectorMap | Morphological or unknown | [14–16] |
| 10.4 | 2.73333 | VectorMap | Morphological or unknown | [14–16] |
| 9.2 | 2.78333 | VectorMap | Morphological or unknown | [14–16] |
| 10.83333 | 2.83333 | VectorMap | Morphological or unknown | [14–16] |
| 9.81666 | 2.98333 | VectorMap | Morphological or unknown | [14–16] |
| 31.66666 | 20.01666 | VectorMap | Morphological or unknown | [14–16] |
| 5.01666 | 20.03333 | VectorMap | Morphological or unknown | [14–16] |
| 32.11666 | 20.06666 | VectorMap | Morphological or unknown | [14–16] |
| 30.76666 | 20.23333 | VectorMap | Morphological or unknown | [14–16] |
| 31.66666 | 20.25 | VectorMap | Morphological or unknown | [14–16] |
| 32.08333 | 20.26666 | VectorMap | Morphological or unknown | [14–16] |
| 15.01666 | 20.66666 | VectorMap | Morphological or unknown | [14–16] |
| 5.76666 | 20.66666 | VectorMap | Morphological or unknown | [14–16] |
| -8.23333 | 20.75 | VectorMap | Morphological or unknown | [14–16] |
| -7.35 | 20.81666 | VectorMap | Morphological or unknown | [14–16] |
| 13.81666 | 20.81666 | VectorMap | Morphological or unknown | [14–16] |
| 6.25 | 21.2 | VectorMap | Morphological or unknown | [14–16] |
| 29.13333 | 21.28333 | VectorMap | Morphological or unknown | [14–16] |
| -24.03333 | 21.9 | VectorMap | Morphological or unknown | [14–16] |
| 17.25 | 22 | VectorMap | Morphological or unknown | [14–16] |
| 15.11666 | 22.25 | VectorMap | Morphological or unknown | [14–16] |
| 24.16666 | 23.25 | VectorMap | Morphological or unknown | [14–16] |
| 12.63333 | 23.31666 | VectorMap | Morphological or unknown | [14–16] |
| -14.4823 | 23.3176 | VectorMap | Morphological or unknown | [14–16] |
| 12.9 | 23.48333 | VectorMap | Morphological or unknown | [14–16] |
| 12.58333 | 23.61666 | VectorMap | Morphological or unknown | [14–16] |
| 13.68333 | 24.5 | VectorMap | Morphological or unknown | [14–16] |
| 29.75 | 24.51666 | VectorMap | Morphological or unknown | [14–16] |
| 2.85 | 24.56666 | VectorMap | Morphological or unknown | [14–16] |
| 9.85 | 24.83333 | VectorMap | Morphological or unknown | [14–16] |
| -14.892 | 24.8659 | VectorMap | Morphological or unknown | [14–16] |
| 12.05 | 24.88333 | VectorMap | Morphological or unknown | [14–16] |
| -5.91666 | 24.9 | VectorMap | Morphological or unknown | [14–16] |
| 0.36666 | 25.25 | VectorMap | Morphological or unknown | [14–16] |
| 12 | 25.63333 | VectorMap | Morphological or unknown | [14–16] |
| -17.89264 | 25.76934 | VectorMap | Morphological or unknown | [14–16] |
| -15.0939 | 26.1824 | VectorMap | Morphological or unknown | [14–16] |
| -15.75 | 26.45 | VectorMap | Morphological or unknown | [14–16] |
| -15.46666 | 26.91666 | VectorMap | Morphological or unknown | [14–16] |
| -14.98333 | 27.06666 | VectorMap | Morphological or unknown | [14–16] |
| 3.41666 | 27.08333 | VectorMap | Morphological or unknown | [14–16] |
| -15.91666 | 27.25 | VectorMap | Morphological or unknown | [14–16] |
| -11.66666 | 27.46666 | VectorMap | Morphological or unknown | [14–16] |
| 5.6 | 27.46666 | VectorMap | Morphological or unknown | [14–16] |
| -16.1471 | 27.5785 | VectorMap | Morphological or unknown | [14–16] |
| -15.8385 | 27.6183 | VectorMap | Morphological or unknown | [14–16] |
| -3.8 | 27.71666 | VectorMap | Morphological or unknown | [14–16] |
| -17.54696 | 27.81314 | VectorMap | Morphological or unknown | [14–16] |
| -15.7985 | 27.8299 | VectorMap | Morphological or unknown | [14–16] |
| -17.41307 | 28.03247 | VectorMap | Morphological or unknown | [14–16] |
| -15.8368 | 28.0517 | VectorMap | Morphological or unknown | [14–16] |
| -14.1662 | 28.0651 | VectorMap | Morphological or unknown | [14–16] |
| -1.73333 | 28.11666 | VectorMap | Morphological or unknown | [14–16] |
| -18.50075 | 28.13449 | VectorMap | Morphological or unknown | [14–16] |
| -11.51666 | 28.2 | VectorMap | Morphological or unknown | [14–16] |
| -21.51247 | 28.23138 | VectorMap | Morphological or unknown | [14–16] |
| -15.574 | 28.2763 | VectorMap | Morphological or unknown | [14–16] |
| -18.24323 | 28.33608 | VectorMap | Morphological or unknown | [14–16] |
| -16.82608 | 28.39636 | VectorMap | Morphological or unknown | [14–16] |
| -14.8948 | 28.412 | VectorMap | Morphological or unknown | [14–16] |
| -16.3 | 28.41666 | VectorMap | Morphological or unknown | [14–16] |
| -15.4108 | 28.4278 | VectorMap | Morphological or unknown | [14–16] |
| -3 | 28.45 | VectorMap | Morphological or unknown | [14–16] |
| -18.51068 | 28.50764 | VectorMap | Morphological or unknown | [14–16] |
| -17.69938 | 28.54282 | VectorMap | Morphological or unknown | [14–16] |
| -18.32262 | 28.56706 | VectorMap | Morphological or unknown | [14–16] |
| -21.27538 | 28.60489 | VectorMap | Morphological or unknown | [14–16] |
| 1.46666 | 28.61666 | VectorMap | Morphological or unknown | [14–16] |
| -12.96666 | 28.63333 | VectorMap | Morphological or unknown | [14–16] |
| 7.85 | 28.65 | VectorMap | Morphological or unknown | [14–16] |
| -17.90411 | 28.66176 | VectorMap | Morphological or unknown | [14–16] |
| -16.1432 | 28.7702 | VectorMap | Morphological or unknown | [14–16] |
| -15.2424 | 28.8203 | VectorMap | Morphological or unknown | [14–16] |
| -16.45293 | 28.86307 | VectorMap | Morphological or unknown | [14–16] |
| -11.18333 | 28.86666 | VectorMap | Morphological or unknown | [14–16] |
| -17.75789 | 28.86893 | VectorMap | Morphological or unknown | [14–16] |
| -18.78826 | 29.03852 | VectorMap | Morphological or unknown | [14–16] |
| -9.78333 | 29.08333 | VectorMap | Morphological or unknown | [14–16] |
| -3.4 | 29.11666 | VectorMap | Morphological or unknown | [14–16] |
| -9.614 | 29.2022 | VectorMap | Morphological or unknown | [14–16] |
| -17.91828 | 29.24452 | VectorMap | Morphological or unknown | [14–16] |
| -0.53333 | 29.3 | VectorMap | Morphological or unknown | [14–16] |
| -18.13999 | 29.35171 | VectorMap | Morphological or unknown | [14–16] |
| -18.61294 | 29.3755 | VectorMap | Morphological or unknown | [14–16] |
| -13.61666 | 29.4 | VectorMap | Morphological or unknown | [14–16] |
| -16.09285 | 29.40812 | VectorMap | Morphological or unknown | [14–16] |
| -17.79462 | 29.43614 | VectorMap | Morphological or unknown | [14–16] |
| 3.03333 | 29.56666 | VectorMap | Morphological or unknown | [14–16] |
| -18.43971 | 29.57022 | VectorMap | Morphological or unknown | [14–16] |
| -11.2766 | 29.6198 | VectorMap | Morphological or unknown | [14–16] |
| -15.08333 | 29.63333 | VectorMap | Morphological or unknown | [14–16] |
| -6.1218 | 29.6973 | VectorMap | Morphological or unknown | [14–16] |
| -16.51996 | 29.75688 | VectorMap | Morphological or unknown | [14–16] |
| -18.00764 | 29.83708 | VectorMap | Morphological or unknown | [14–16] |
| -15.0028 | 29.839 | VectorMap | Morphological or unknown | [14–16] |
| -17.60863 | 29.88931 | VectorMap | Morphological or unknown | [14–16] |
| -18.29254 | 29.90803 | VectorMap | Morphological or unknown | [14–16] |
| 6.05 | 29.93333 | VectorMap | Morphological or unknown | [14–16] |
| 10.21666 | 3.16666 | VectorMap | Morphological or unknown | [14–16] |
| 9.93333 | 3.2 | VectorMap | Morphological or unknown | [14–16] |
| 10.3 | 3.38333 | VectorMap | Morphological or unknown | [14–16] |
| 11.86666 | 3.38333 | VectorMap | Morphological or unknown | [14–16] |
| 10.68333 | 3.8 | VectorMap | Morphological or unknown | [14–16] |
| -22.21666 | 30 | VectorMap | Morphological or unknown | [14–16] |
| 10.56666 | 30.13333 | VectorMap | Morphological or unknown | [14–16] |
| 8.3 | 30.13333 | VectorMap | Morphological or unknown | [14–16] |
| 1.58333 | 30.21666 | VectorMap | Morphological or unknown | [14–16] |
| 13.18333 | 30.21666 | VectorMap | Morphological or unknown | [14–16] |
| -6.7546 | 30.4058 | VectorMap | Morphological or unknown | [14–16] |
| 11.86666 | 30.46666 | VectorMap | Morphological or unknown | [14–16] |
| -2.7076 | 30.49 | VectorMap | Morphological or unknown | [14–16] |
| 6.55 | 30.5 | VectorMap | Morphological or unknown | [14–16] |
| 11.21666 | 30.51666 | VectorMap | Morphological or unknown | [14–16] |
| 10.75 | 30.56666 | VectorMap | Morphological or unknown | [14–16] |
| -7.4078 | 30.5915 | VectorMap | Morphological or unknown | [14–16] |
| 4.08333 | 30.66666 | VectorMap | Morphological or unknown | [14–16] |
| -21.35538 | 30.72297 | VectorMap | Morphological or unknown | [14–16] |
| 9.06666 | 30.88333 | VectorMap | Morphological or unknown | [14–16] |
| -7.9456 | 30.8905 | VectorMap | Morphological or unknown | [14–16] |
| -2.5114 | 30.9727 | VectorMap | Morphological or unknown | [14–16] |
| -16.37253 | 31.03762 | VectorMap | Morphological or unknown | [14–16] |
| -7.4301 | 31.0607 | VectorMap | Morphological or unknown | [14–16] |
| 30.05 | 31.25 | VectorMap | Morphological or unknown | [14–16] |
| -22.43333 | 31.33333 | VectorMap | Morphological or unknown | [14–16] |
| -10.0648 | 31.3452 | VectorMap | Morphological or unknown | [14–16] |
| 21.8 | 31.35 | VectorMap | Morphological or unknown | [14–16] |
| -14.2575 | 31.3751 | VectorMap | Morphological or unknown | [14–16] |
| -22.07478 | 31.38595 | VectorMap | Morphological or unknown | [14–16] |
| -5.7632 | 31.4586 | VectorMap | Morphological or unknown | [14–16] |
| 6.2 | 31.55 | VectorMap | Morphological or unknown | [14–16] |
| -16.28607 | 31.56997 | VectorMap | Morphological or unknown | [14–16] |
| 4.85 | 31.6 | VectorMap | Morphological or unknown | [14–16] |
| 9.51666 | 31.65 | VectorMap | Morphological or unknown | [14–16] |
| 18.25 | 31.66666 | VectorMap | Morphological or unknown | [14–16] |
| 3.88333 | 31.66666 | VectorMap | Morphological or unknown | [14–16] |
| -20.98484 | 31.68277 | VectorMap | Morphological or unknown | [14–16] |
| -14.2784 | 31.7116 | VectorMap | Morphological or unknown | [14–16] |
| -7.7661 | 31.7481 | VectorMap | Morphological or unknown | [14–16] |
| 5.2 | 31.76666 | VectorMap | Morphological or unknown | [14–16] |
| -16.46601 | 31.8306 | VectorMap | Morphological or unknown | [14–16] |
| -3.382 | 31.9155 | VectorMap | Morphological or unknown | [14–16] |
| 4.7 | 31.91666 | VectorMap | Morphological or unknown | [14–16] |
| -2.9092 | 31.9403 | VectorMap | Morphological or unknown | [14–16] |
| -9.0822 | 31.9473 | VectorMap | Morphological or unknown | [14–16] |
| -14.0298 | 31.979 | VectorMap | Morphological or unknown | [14–16] |
| 3.6 | 32.05 | VectorMap | Morphological or unknown | [14–16] |
| 3.93333 | 32.05 | VectorMap | Morphological or unknown | [14–16] |
| -10.55 | 32.06666 | VectorMap | Morphological or unknown | [14–16] |
| -2.7572 | 32.1566 | VectorMap | Morphological or unknown | [14–16] |
| -10.03333 | 32.16666 | VectorMap | Morphological or unknown | [14–16] |
| -11.91666 | 32.16666 | VectorMap | Morphological or unknown | [14–16] |
| -20.04841 | 32.18916 | VectorMap | Morphological or unknown | [14–16] |
| 10.43333 | 32.2 | VectorMap | Morphological or unknown | [14–16] |
| -27.63333 | 32.25 | VectorMap | Morphological or unknown | [14–16] |
| -2.4497 | 32.2687 | VectorMap | Morphological or unknown | [14–16] |
| 4.13333 | 32.28333 | VectorMap | Morphological or unknown | [14–16] |
| 4.31666 | 32.36666 | VectorMap | Morphological or unknown | [14–16] |
| -14.1983 | 32.3839 | VectorMap | Morphological or unknown | [14–16] |
| -9.02 | 32.4466 | VectorMap | Morphological or unknown | [14–16] |
| 0.06666 | 32.46666 | VectorMap | Morphological or unknown | [14–16] |
| -3.9267 | 32.4769 | VectorMap | Morphological or unknown | [14–16] |
| 3.85 | 32.5 | VectorMap | Morphological or unknown | [14–16] |
| -3.4321 | 32.5043 | VectorMap | Morphological or unknown | [14–16] |
| 15.6 | 32.53333 | VectorMap | Morphological or unknown | [14–16] |
| -8.849 | 32.5336 | VectorMap | Morphological or unknown | [14–16] |
| -14.0297 | 32.5817 | VectorMap | Morphological or unknown | [14–16] |
| -24.05 | 32.61666 | VectorMap | Morphological or unknown | [14–16] |
| -10.13333 | 32.63333 | VectorMap | Morphological or unknown | [14–16] |
| 4.63333 | 32.63333 | VectorMap | Morphological or unknown | [14–16] |
| -3.8114 | 32.6422 | VectorMap | Morphological or unknown | [14–16] |
| -13.5174 | 32.6428 | VectorMap | Morphological or unknown | [14–16] |
| 4.31666 | 32.73333 | VectorMap | Morphological or unknown | [14–16] |
| -14.0497 | 32.7805 | VectorMap | Morphological or unknown | [14–16] |
| 4.03333 | 32.78333 | VectorMap | Morphological or unknown | [14–16] |
| -9.2509 | 32.785 | VectorMap | Morphological or unknown | [14–16] |
| -4.9338 | 32.8067 | VectorMap | Morphological or unknown | [14–16] |
| -8.2966 | 32.8153 | VectorMap | Morphological or unknown | [14–16] |
| 4.5 | 32.83333 | VectorMap | Morphological or unknown | [14–16] |
| -13.2262 | 32.8822 | VectorMap | Morphological or unknown | [14–16] |
| -9.0617 | 32.9096 | VectorMap | Morphological or unknown | [14–16] |
| -2.4463 | 32.9208 | VectorMap | Morphological or unknown | [14–16] |
| 7.78333 | 33.01666 | VectorMap | Morphological or unknown | [14–16] |
| -2.6485 | 33.0368 | VectorMap | Morphological or unknown | [14–16] |
| -3.0044 | 33.0648 | VectorMap | Morphological or unknown | [14–16] |
| -8.3254 | 33.0813 | VectorMap | Morphological or unknown | [14–16] |
| 4.08333 | 33.1 | VectorMap | Morphological or unknown | [14–16] |
| -2.8339 | 33.1384 | VectorMap | Morphological or unknown | [14–16] |
| -4.1578 | 33.1843 | VectorMap | Morphological or unknown | [14–16] |
| -3.3384 | 33.284 | VectorMap | Morphological or unknown | [14–16] |
| -12.2708 | 33.2842 | VectorMap | Morphological or unknown | [14–16] |
| 19.53333 | 33.31666 | VectorMap | Morphological or unknown | [14–16] |
| -3.1571 | 33.3408 | VectorMap | Morphological or unknown | [14–16] |
| -8.576 | 33.3446 | VectorMap | Morphological or unknown | [14–16] |
| -6.7641 | 33.3865 | VectorMap | Morphological or unknown | [14–16] |
| -3.5744 | 33.4349 | VectorMap | Morphological or unknown | [14–16] |
| 14.4 | 33.53333 | VectorMap | Morphological or unknown | [14–16] |
| -3.3717 | 33.5511 | VectorMap | Morphological or unknown | [14–16] |
| -2.8798 | 33.5531 | VectorMap | Morphological or unknown | [14–16] |
| 4.83333 | 33.56666 | VectorMap | Morphological or unknown | [14–16] |
| -2.3679 | 33.6024 | VectorMap | Morphological or unknown | [14–16] |
| -8.4714 | 33.7324 | VectorMap | Morphological or unknown | [14–16] |
| -3.0907 | 33.7429 | VectorMap | Morphological or unknown | [14–16] |
| -1.4298 | 33.8141 | VectorMap | Morphological or unknown | [14–16] |
| -3.3549 | 33.8162 | VectorMap | Morphological or unknown | [14–16] |
| -8.689 | 33.858 | VectorMap | Morphological or unknown | [14–16] |
| -21.43333 | 33.86666 | VectorMap | Morphological or unknown | [14–16] |
| -9.71666 | 33.93333 | VectorMap | Morphological or unknown | [14–16] |
| 13.15 | 33.93333 | VectorMap | Morphological or unknown | [14–16] |
| 8.26666 | 33.95 | VectorMap | Morphological or unknown | [14–16] |
| -4.7065 | 34.024 | VectorMap | Morphological or unknown | [14–16] |
| -8.2173 | 34.0631 | VectorMap | Morphological or unknown | [14–16] |
| 17.56666 | 34.06666 | VectorMap | Morphological or unknown | [14–16] |
| -4.377 | 34.1882 | VectorMap | Morphological or unknown | [14–16] |
| -13.66666 | 34.26666 | VectorMap | Morphological or unknown | [14–16] |
| -5.7374 | 34.3198 | VectorMap | Morphological or unknown | [14–16] |
| -19.7 | 34.33333 | VectorMap | Morphological or unknown | [14–16] |
| -8.5571 | 34.3391 | VectorMap | Morphological or unknown | [14–16] |
| -1.2817 | 34.3618 | VectorMap | Morphological or unknown | [14–16] |
| -8.784 | 34.3716 | VectorMap | Morphological or unknown | [14–16] |
| -3.9678 | 34.4037 | VectorMap | Morphological or unknown | [14–16] |
| 6.16666 | 34.46666 | VectorMap | Morphological or unknown | [14–16] |
| -5.6173 | 34.5431 | VectorMap | Morphological or unknown | [14–16] |
| -8.2024 | 34.5519 | VectorMap | Morphological or unknown | [14–16] |
| 0.03333 | 34.6 | VectorMap | Morphological or unknown | [14–16] |
| 3.763 | 34.6015 | VectorMap | Morphological or unknown | [14–16] |
| -4.3847 | 34.6224 | VectorMap | Morphological or unknown | [14–16] |
| 4.75 | 34.66666 | VectorMap | Morphological or unknown | [14–16] |
| -0.91666 | 34.68333 | VectorMap | Morphological or unknown | [14–16] |
| -1.2194 | 34.7874 | VectorMap | Morphological or unknown | [14–16] |
| -5.75 | 34.85 | VectorMap | Morphological or unknown | [14–16] |
| -23.83333 | 34.95 | VectorMap | Morphological or unknown | [14–16] |
| -6.3492 | 34.9884 | VectorMap | Morphological or unknown | [14–16] |
| -20.96666 | 35 | VectorMap | Morphological or unknown | [14–16] |
| 0.66666 | 35.15 | VectorMap | Morphological or unknown | [14–16] |
| -13.31666 | 35.23333 | VectorMap | Morphological or unknown | [14–16] |
| -3.83333 | 35.25 | VectorMap | Morphological or unknown | [14–16] |
| -6.9338 | 35.2689 | VectorMap | Morphological or unknown | [14–16] |
| -6.3554 | 35.3283 | VectorMap | Morphological or unknown | [14–16] |
| -15.38333 | 35.33333 | VectorMap | Morphological or unknown | [14–16] |
| -5.1611 | 35.463 | VectorMap | Morphological or unknown | [14–16] |
| 0.7173 | 35.5955 | VectorMap | Morphological or unknown | [14–16] |
| 8.38333 | 35.6 | VectorMap | Morphological or unknown | [14–16] |
| -7.1334 | 35.6064 | VectorMap | Morphological or unknown | [14–16] |
| 0.0385 | 35.7124 | VectorMap | Morphological or unknown | [14–16] |
| -6.1305 | 35.781 | VectorMap | Morphological or unknown | [14–16] |
| -7.5248 | 35.7962 | VectorMap | Morphological or unknown | [14–16] |
| -1.1183 | 35.8473 | VectorMap | Morphological or unknown | [14–16] |
| -7.0947 | 35.8486 | VectorMap | Morphological or unknown | [14–16] |
| -3.36666 | 35.86666 | VectorMap | Morphological or unknown | [14–16] |
| -3.6763 | 35.9287 | VectorMap | Morphological or unknown | [14–16] |
| 15.06666 | 35.95 | VectorMap | Morphological or unknown | [14–16] |
| -4.4787 | 35.9937 | VectorMap | Morphological or unknown | [14–16] |
| -2.1018 | 36.0731 | VectorMap | Morphological or unknown | [14–16] |
| 0.38333 | 36.08333 | VectorMap | Morphological or unknown | [14–16] |
| 0.63333 | 36.08333 | VectorMap | Morphological or unknown | [14–16] |
| 0.03333 | 36.15 | VectorMap | Morphological or unknown | [14–16] |
| -4.7403 | 36.2025 | VectorMap | Morphological or unknown | [14–16] |
| -1.9 | 36.28333 | VectorMap | Morphological or unknown | [14–16] |
| 6.93333 | 36.33333 | VectorMap | Morphological or unknown | [14–16] |
| -1.0144 | 36.3678 | VectorMap | Morphological or unknown | [14–16] |
| 15.46666 | 36.4 | VectorMap | Morphological or unknown | [14–16] |
| -5.6295 | 36.4036 | VectorMap | Morphological or unknown | [14–16] |
| -10.5995 | 36.4213 | VectorMap | Morphological or unknown | [14–16] |
| -1.4526 | 36.4977 | VectorMap | Morphological or unknown | [14–16] |
| -2.1 | 36.5015 | VectorMap | Morphological or unknown | [14–16] |
| -6.3302 | 36.5201 | VectorMap | Morphological or unknown | [14–16] |
| -0.93333 | 36.56666 | VectorMap | Morphological or unknown | [14–16] |
| -8.46666 | 36.63333 | VectorMap | Morphological or unknown | [14–16] |
| 0.15 | 36.63333 | VectorMap | Morphological or unknown | [14–16] |
| -1.18333 | 36.65 | VectorMap | Morphological or unknown | [14–16] |
| -1.85 | 36.78333 | VectorMap | Morphological or unknown | [14–16] |
| -8.0861 | 36.7846 | VectorMap | Morphological or unknown | [14–16] |
| -2.4923 | 36.8052 | VectorMap | Morphological or unknown | [14–16] |
| 18.83333 | 36.83333 | VectorMap | Morphological or unknown | [14–16] |
| -1.2837 | 36.8667 | VectorMap | Morphological or unknown | [14–16] |
| -9.0511 | 36.8716 | VectorMap | Morphological or unknown | [14–16] |
| -3.0995 | 36.8736 | VectorMap | Morphological or unknown | [14–16] |
| -0.41666 | 36.95 | VectorMap | Morphological or unknown | [14–16] |
| -7.8316 | 36.9834 | VectorMap | Morphological or unknown | [14–16] |
| -1.6536 | 36.9964 | VectorMap | Morphological or unknown | [14–16] |
| -14.88333 | 37 | VectorMap | Morphological or unknown | [14–16] |
| -6.8231 | 37.0274 | VectorMap | Morphological or unknown | [14–16] |
| 0.53333 | 37.03333 | VectorMap | Morphological or unknown | [14–16] |
| -5.97 | 37.0365 | VectorMap | Morphological or unknown | [14–16] |
| -10.9772 | 37.0625 | VectorMap | Morphological or unknown | [14–16] |
| 0.01666 | 37.06666 | VectorMap | Morphological or unknown | [14–16] |
| -1.8205 | 37.1757 | VectorMap | Morphological or unknown | [14–16] |
| -6.1913 | 37.1996 | VectorMap | Morphological or unknown | [14–16] |
| -1.1157 | 37.2189 | VectorMap | Morphological or unknown | [14–16] |
| 19.61666 | 37.23333 | VectorMap | Morphological or unknown | [14–16] |
| -0.7 | 37.35 | VectorMap | Morphological or unknown | [14–16] |
| -2.0428 | 37.3787 | VectorMap | Morphological or unknown | [14–16] |
| -6.7141 | 37.385 | VectorMap | Morphological or unknown | [14–16] |
| -6.3172 | 37.477 | VectorMap | Morphological or unknown | [14–16] |
| 0.3943 | 37.477 | VectorMap | Morphological or unknown | [14–16] |
| -1.11666 | 37.48333 | VectorMap | Morphological or unknown | [14–16] |
| -0.7854 | 37.557 | VectorMap | Morphological or unknown | [14–16] |
| -0.4033 | 37.5913 | VectorMap | Morphological or unknown | [14–16] |
| 0.05 | 37.65 | VectorMap | Morphological or unknown | [14–16] |
| 0.2897 | 37.661 | VectorMap | Morphological or unknown | [14–16] |
| -6.3025 | 37.6925 | VectorMap | Morphological or unknown | [14–16] |
| -6.7958 | 37.7074 | VectorMap | Morphological or unknown | [14–16] |
| -2.18333 | 37.71666 | VectorMap | Morphological or unknown | [14–16] |
| -2.7717 | 37.7188 | VectorMap | Morphological or unknown | [14–16] |
| 18.43333 | 37.73333 | VectorMap | Morphological or unknown | [14–16] |
| 1.3648 | 37.7406 | VectorMap | Morphological or unknown | [14–16] |
| -7.2586 | 37.7698 | VectorMap | Morphological or unknown | [14–16] |
| -1.63333 | 37.88333 | VectorMap | Morphological or unknown | [14–16] |
| 0.021 | 37.9467 | VectorMap | Morphological or unknown | [14–16] |
| -9.8 | 37.96666 | VectorMap | Morphological or unknown | [14–16] |
| -1.8061 | 37.9676 | VectorMap | Morphological or unknown | [14–16] |
| 2.33333 | 37.98333 | VectorMap | Morphological or unknown | [14–16] |
| -5.3895 | 38.1024 | VectorMap | Morphological or unknown | [14–16] |
| -3.4 | 38.13333 | VectorMap | Morphological or unknown | [14–16] |
| -3.6398 | 38.1958 | VectorMap | Morphological or unknown | [14–16] |
| -10.7156 | 38.4191 | VectorMap | Morphological or unknown | [14–16] |
| 0.53333 | 38.51666 | VectorMap | Morphological or unknown | [14–16] |
| -5.7806 | 38.5245 | VectorMap | Morphological or unknown | [14–16] |
| -5.1075 | 38.5421 | VectorMap | Morphological or unknown | [14–16] |
| -3.38333 | 38.56666 | VectorMap | Morphological or unknown | [14–16] |
| -10.4088 | 38.7307 | VectorMap | Morphological or unknown | [14–16] |
| -6.4328 | 38.7767 | VectorMap | Morphological or unknown | [14–16] |
| -10.7875 | 38.7791 | VectorMap | Morphological or unknown | [14–16] |
| -6.679 | 38.8139 | VectorMap | Morphological or unknown | [14–16] |
| 3.4865 | 39.0709 | VectorMap | Morphological or unknown | [14–16] |
| -8.0279 | 39.1064 | VectorMap | Morphological or unknown | [14–16] |
| -9.76 | 39.1244 | VectorMap | Morphological or unknown | [14–16] |
| -4.6227 | 39.2233 | VectorMap | Morphological or unknown | [14–16] |
| -6.8489 | 39.2616 | VectorMap | Morphological or unknown | [14–16] |
| -4.1425 | 39.2829 | VectorMap | Morphological or unknown | [14–16] |
| -10.3665 | 39.3661 | VectorMap | Morphological or unknown | [14–16] |
| -8.129 | 39.3828 | VectorMap | Morphological or unknown | [14–16] |
| -8.8297 | 39.4412 | VectorMap | Morphological or unknown | [14–16] |
| -4.454 | 39.4537 | VectorMap | Morphological or unknown | [14–16] |
| -4.2885 | 39.5466 | VectorMap | Morphological or unknown | [14–16] |
| -3.9449 | 39.6016 | VectorMap | Morphological or unknown | [14–16] |
| -10.1676 | 39.6287 | VectorMap | Morphological or unknown | [14–16] |
| -0.46666 | 39.63333 | VectorMap | Morphological or unknown | [14–16] |
| -3.63333 | 39.85 | VectorMap | Morphological or unknown | [14–16] |
| -3.3166 | 40.0234 | VectorMap | Morphological or unknown | [14–16] |
| 1.75 | 40.06666 | VectorMap | Morphological or unknown | [14–16] |
| -10.3661 | 40.1706 | VectorMap | Morphological or unknown | [14–16] |
| -2.63333 | 40.2 | VectorMap | Morphological or unknown | [14–16] |
| -2.23333 | 40.81666 | VectorMap | Morphological or unknown | [14–16] |
| 11.1 | 42.38333 | VectorMap | Morphological or unknown | [14–16] |
| 11.16666 | 42.7 | VectorMap | Morphological or unknown | [14–16] |
| 2.15 | 45.11666 | VectorMap | Morphological or unknown | [14–16] |
| 2.06666 | 45.36666 | VectorMap | Morphological or unknown | [14–16] |
| 14.61666 | 6.15 | VectorMap | Morphological or unknown | [14–16] |
| 16.96666 | 7.98333 | VectorMap | Morphological or unknown | [14–16] |
| 13.8 | 8.98333 | VectorMap | Morphological or unknown | [14–16] |
| 13.06666 | 9.33333 | VectorMap | Morphological or unknown | [14–16] |
| 30.13333 | 9.5 | VectorMap | Morphological or unknown | [14–16] |
| 4.05 | 9.7 | VectorMap | Morphological or unknown | [14–16] |
| 2.95 | 9.91666 | VectorMap | Morphological or unknown | [14–16] |
| 9.104568 | 99.376796 | Literature | Morphological or unknown | [17] |
| 10.2 | -83.683333 | Literature | Morphological or unknown | [18] |
| 10.216667 | -83.783333 | Literature | Morphological or unknown | [18] |
| 32.6937 | -114.6277 | Literature | Molecular | [19] |
| 32.6245 | -115.4523 | Literature | Molecular | [19] |
| 33.0114 | -115.4734 | Literature | Molecular | [19] |
| 32.6789 | -115.4989 | Literature | Molecular | [19] |
| 17.0594 | -96.7216 | Literature | Molecular | [19] |
| 23.966667 | 120.966667 | Literature | Morphological or unknown | [20] |

*Included in the model informed by coordinates of only molecular confirmed *Rh. sanguineus* observations, but excluded from the model informed by all *Rh. sanguineus* locations after it was noticed that the coordinate did not include two decimal places or more for either the latitude or longitude.

1. Chao L-L, Hsieh C-K, Ho T-Y, Shih C-M. First zootiological survey of hard ticks (Acari: Ixodidae) infesting dogs in northern Taiwan. Exp Appl Acarol. 2019;77: 105–115. doi:10.1007/s10493-018-0328-x

2. Cruz-Vazquez C, Garcia-Vazquez Z. Seasonal distribution of *Rhipicephalus sanguineus* ticks (Acari: Ixodidae) on dogs in an urban area of Morelos, Mexico. Exp Appl Acarol. 1999;23: 277–280.

3. Da Silva CB, Santos HA, Navarrete MG, Ribeiro CCDU, Gonzalez BC, Zaldivar MF, et al. Molecular detection and characterization of *Anaplasma platys* in dogs and ticks in Cuba. Ticks Tick Borne Dis. 2016;7: 938–944. doi:10.1016/j.ttbdis.2016.04.012

4. Galaviz-Silva L, Pérez-Treviño KC, Molina-Garza ZJ. Distribution of ixodid ticks on dogs in Nuevo León, Mexico, and their association with *Borrelia burgdorferi* sensu lato. Exp Appl Acarol. 2013;61: 491–501. doi:10.1007/s10493-013-9707-5

5. Guzmán PEE, Soto YB, Rodríguez-Mallon A. Genetic and biological characterization of a Cuban tick strain from *Rhipicephalus sanguineus* complex and its sensitivity to different chemical acaricides. Int J Acarology. 2016;42: 18–25. doi:10.1080/01647954.2015.1113309

6. Inokuma H, Tamura K, Onishi T. Seasonal occurrence of *Rhipicephalus sanguineus* in Okayama Prefecture, Japan and effect of temperature on development of the tick. J Vet Med Sci. 1996;58: 225–228. doi:10.1292/jvms.58.225

7. Noda AA, Rodríguez I, Miranda J, Contreras V, Mattar S. First molecular evidence of *Coxiella burnetii* infecting ticks in Cuba. Ticks and Tick-borne Diseases. 2016;7: 68–70. doi:10.1016/j.ttbdis.2015.08.008

8. Ojeda-Chi MM, Rodriguez-Vivas RI, Esteve-Gasent MD, Pérez de León AA, Modarelli JJ, Villegas-Perez SL. Ticks infesting dogs in rural communities of Yucatan, Mexico and molecular diagnosis of rickettsial infection. Transbound Emerg Dis. 2019;66: 102–110. doi:10.1111/tbed.12990

9. Ortega-Morales AI, Nava-Reyna E, Ávila-Rodríguez V, González-Álvarez VH, Castillo-Martínez A, Siller-Rodríguez QK, et al. Detection of *Rickettsia* spp. in *Rhipicephalus sanguineus* (sensu lato) collected from free-roaming dogs in Coahuila state, northern Mexico. Parasites & Vectors. 2019;12: 130. doi:10.1186/s13071-019-3377-z

10. Peniche-Lara G, Jimenez-Delgadillo B, Dzul-Rosado K. *Rickettsia rickettsii* and *Rickettsia felis* infection in *Rhipicephalus sanguineus* ticks and *Ctenocephalides felis* fleas co-existing in a small city in Yucatan, Mexico. J Vector Ecol. 2015;40: 422–424. doi:10.1111/jvec.12185

11. Peniche-Lara G, Jimenez-Delgadillo B, Munoz-Zanzi C, Cárdenas-Marrufo M, Pérez-Osorio C, Arias-León J. Presence of *Rickettsia* species in a marginalized area of Yucatan, Mexico. J Trop Med. 2018;2018. doi:10.1155/2018/7675828

12. Reeves WK, Durden LA, Iwakami M, Vince KJ, Paul RR. Rickettsial diseases and ectoparasites from military bases in Japan. J Parasitol. 2015;101: 150–155. doi:10.1645/14-662.1

13. Sanprick A, Yooyen T, Rodkvamtook W. Survey of *Rickettsia* spp. and *Orientia tsutsugamushi* pathogens found in animal vectors (ticks, fleas, chiggers) in Bangkaew district, Phatthalung province, Thailand. Korean J Parasitol. 2019;57: 167–173. doi:10.3347/kjp.2019.57.2.167

14. Stromdahl EY, Jiang J, Vince M, Richards AL. Infrequency of *Rickettsia rickettsii* in *Dermacentor variabilis* removed from humans, with comments on the role of other human-biting ticks associated with spotted fever group Rickettsiae in the United States. Vector Borne Zoonotic Dis. 2011;11: 969–977. doi:10.1089/vbz.2010.0099

15. Stromdahl EY, Evans SR, O’Brien JJ, Gutierrez AG. Prevalence of infection in ticks submitted to the human tick test kit program of the U.S. Army Center for Health Promotion and Preventive Medicine. J Med Entomol. 2001;38: 67–74.

16. Stromdahl EY, Williamson PC, Kollars TM, Evans SR, Barry RK, Vince MA, et al. Evidence of *Borrelia lonestari* DNA in *Amblyomma americanum* (Acari: Ixodidae) removed from humans. J Clin Microbiol. 2003;41: 5557–5562.

17. Trinachartvanit W, Rakthong P, Baimai V, Ahantarig A. *Candidatus* Midichloria sp in a *Rhipicephalus sanguineus* s.l. nymphal tick collected from a cat in Thailand. Southeast Asian J Trop Med; Bangkok. 2018;49: 251–255.

18. Troyo A, Moreira-Soto RD, Calderon-Arguedas Ó, Mata-Somarribas C, Ortiz-Tello J, Barbieri ARM, et al. Detection of rickettsiae in fleas and ticks from areas of Costa Rica with history of spotted fever group rickettsioses. Ticks Tick Borne Dis. 2016;7: 1128–1134. doi:10.1016/j.ttbdis.2016.08.009

19. Villarreal Z, Stephenson N, Foley J. Possible northward introgression of a tropical lineage of *Rhipicephalus sanguineus* ticks at a site of emerging Rocky Mountain spotted fever. J Parasitol. 2018;104: 240–245. doi:10.1645/18-10

20. Yuasa Y, Tsai Y-L, Chang C-C, Hsu T-H, Chou C-C. The prevalence of *Anaplasma platys* and a potential novel *Anaplasma* species exceed that of *Ehrlichia canis* in asymptomatic dogs and *Rhipicephalus sanguineus* in Taiwan. J Vet Med Sci. 2017;79: 1494–1502. doi:10.1292/jvms.17-0224

**Results**

**Global *Rh. sanguineus*** **model**

From an initial dataset of 8,015 quality-controlled global *Rh. sanguineus* geolocations, 1,305 were retained for use in the model following the removal of locations duplicated within a 1 km² environmental predictor raster (Table S1). The optimal model included ten variables: four related to temperature, two to precipitation, elevation, slope, average NDVI and NDVI standard deviation. Annual temperature range impacted habitat suitability most (38.46%), followed by annual precipitation (18.69%) and average NDVI (14.42%; Table S2). All other variables influenced suitability by less than 10%. Following ten iterations, the optimal global model had a mean AUC of 0.73 (range = 0.71–0.78) and a mean maximum specificity + sensitivity of 0.58 (0.521–0.63).

**Table S2:** Mean model outputs from 10 iterations of MaxEnt models to predict habitat suitability in the U.S. for ticks assumed to be of the the tropical lineage of *Rhipicephalus sanguineus* using global geolocations*.*

| **Environmental Predictor Variable** | **Percent Contribution** | **Permutation Importance** |
| --- | --- | --- |
| BIO3: Isothermality | 3.39 | 0.27 |
| BIO7: Annual temperature range | 36.64 | 38.46 |
| BIO10: Mean temperature of warmest quarter | 4.04 | 7.27 |
| BIO 11: Mean temperature of the coldest quarter | 0.02 | 0.93 |
| BIO12: Annual precipitation | 4.81 | 18.69 |
| BIO14: Precipitation of driest month | 0.07 | 0.31 |
| Elevation | 4.44 | 4.78 |
| Slope | 2.35 | 5.22 |
| NDVI average | 6.58 | 14.42 |
| NDVI standard deviation | 37.68 | 9.66 |

The model predicted that habitat currently exists for *Rh. sanguineus* across approximately 80,700 km^2^ of the U.S., comprising the entire southern coastal region of California, extending as far north as the San Francisco Bay area, with patchy habitat also present on the northern California coast. In addition, patchy habitat exists along the coast of Oregon, Washington, Texas, Louisiana and Florida, and in Colorado (Figure S1). When considering just California, the Tehachapi extending east into the southern Sierra mountain range had relatively high suitability, although when considering the binary threshold of suitability only some areas of the Sierra mountains were suitable.


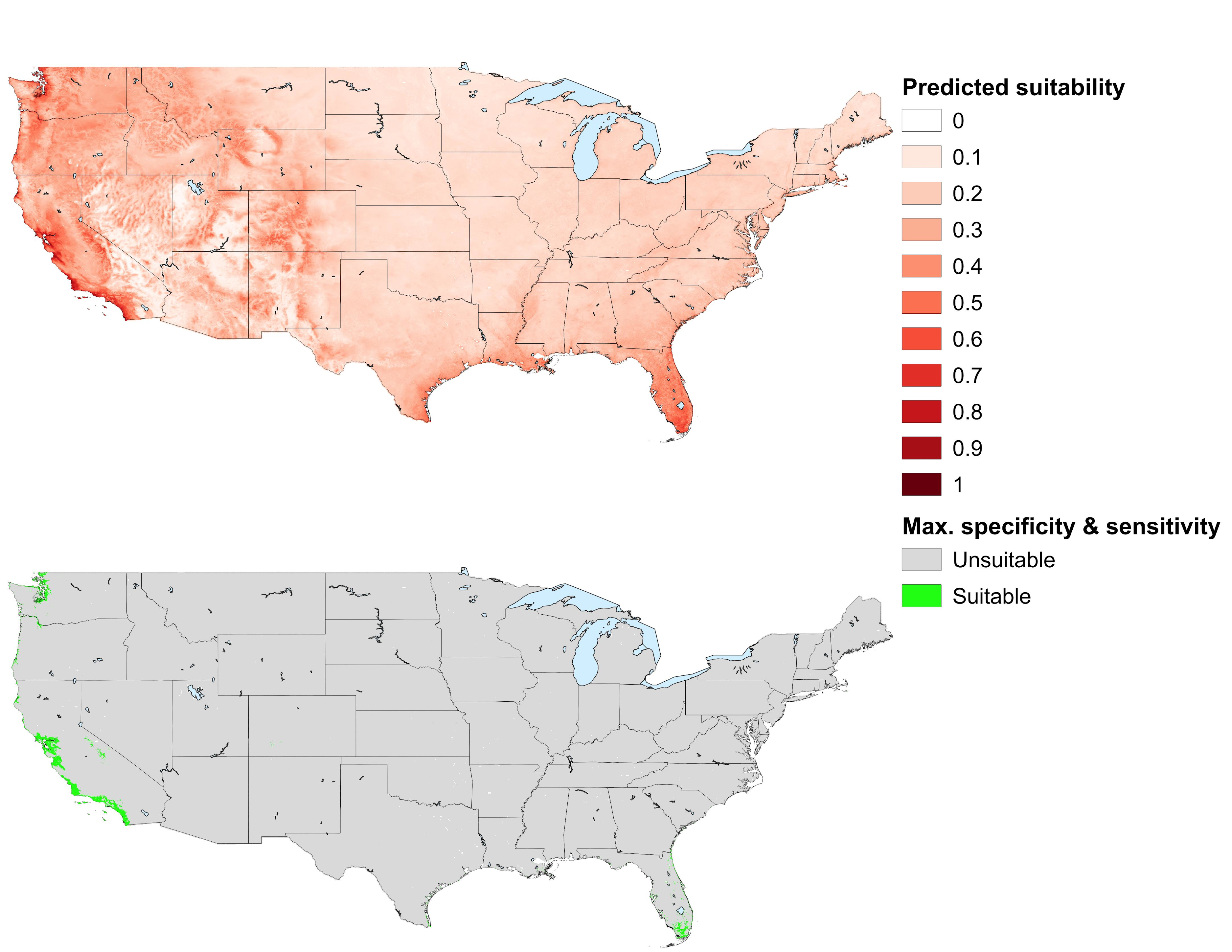


**Figure S1.** **Tropical lineage *Rhipicephalus sanguineus* suitability maps from global geolocation data.** Habitat in the U.S. predicted (using MaxEnt species distribution modeling) to be environmentally suitable for ticks assumed to be of the tropical lineage of *Rhipicephalus sanguineus* under current (2015–2019) climatic conditions. Suitability is visualized as a heatmap and a binary probability threshold that represented the mean maximum true positive rate at the maximum true negative rate across all ten models.

Both precipitation during the driest month and annual precipitation were negatively associated with habitat suitability, such that locations receiving more than 2,500 mm of precipitation annually were less than 50% suitable (Figure S2). Habitat was most suitable in areas where temperatures were relatively high and stable; habitat was positively correlated with mean temperature during the coldest quarter, and negatively associated with annual temperature range and mean temperature of the warmest quarter. However even in areas where the mean temperature of the warmest quarter was close to 40°C suitability remained above 50%, and suitability declined sharply where the annual temperature range was more than 25°C. Predicted suitability was negatively correlated with mean NDVI values but positively associated with NDVI standard deviation (a proxy for land use change).


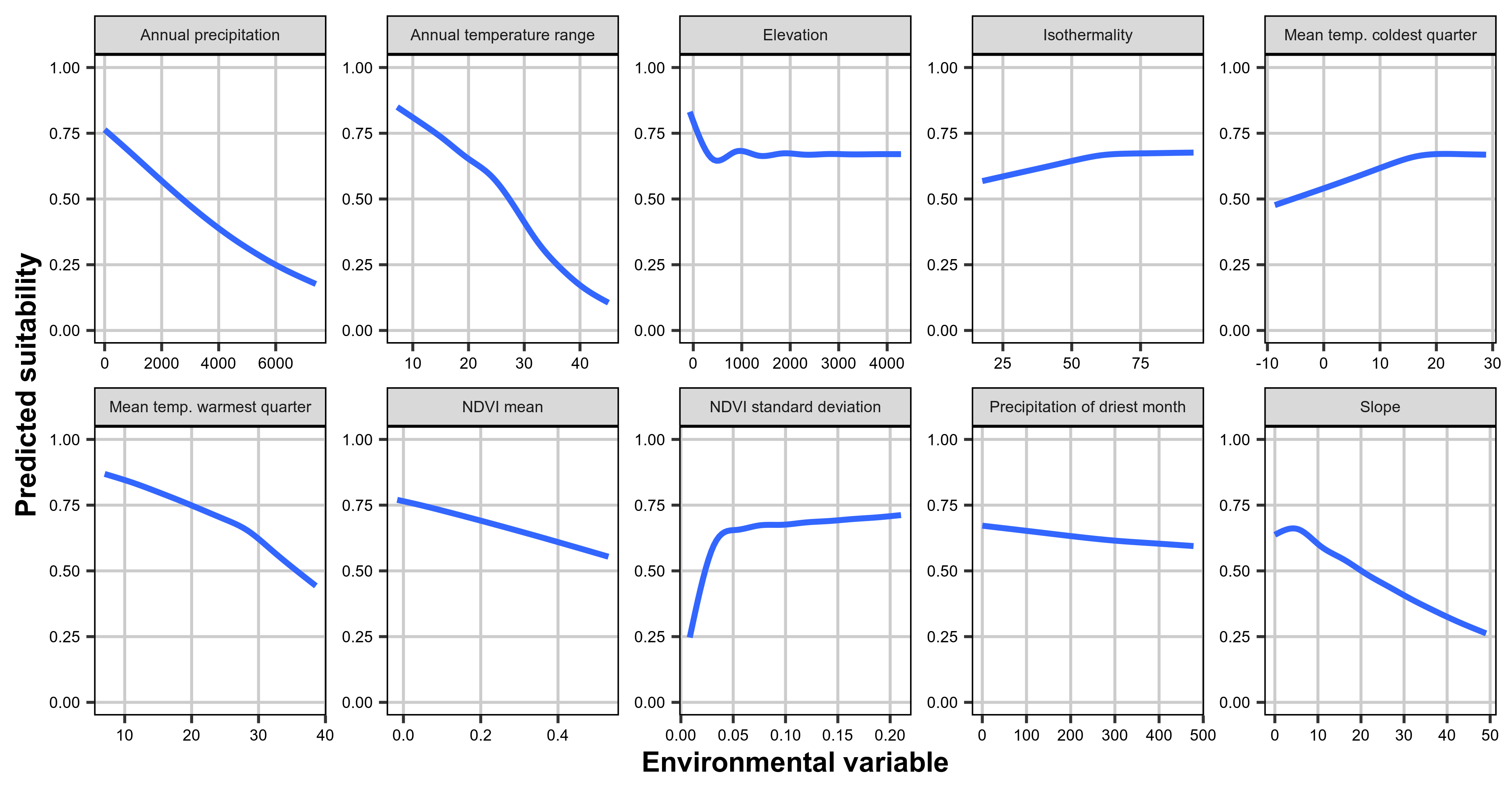


**Figure S2: Tropical lineage *Rhipicephalus sanguineus* species distribution modeling response curves from global geolocation data.** Estimated smoothed trends (using GAM) between environmental predictor variables and predicted habitat suitability in MaxEnt models for ticks assumed to be of the tropical lineage of *Rhipicephalus sanguineus* using global geolocations*.* Trends were derived from the ratio of probability density of each predictor at presence to background geolocations, considering data from 10 iterations used for model training and testing.

**Ensemble tropical *Rh. sanguineus*** **model: Current habitat prediction**

Preliminary analyses used an ensemble model to predict current habitat of tropical *Rh. sanguineus* using presence points confirmed by molecular methods. The ensemble model combining the outputs of the tropical *Rh. sanguineus* model implementing the MaxEnt algorithm optimised for the current habitat (2015–2019) predictions, with random forest, boosted tree regression and generalized linear models was run using the “sdm” package in R [5]. Each model was iterated ten times, using a different combination of one test subset and nine training subsets for each iteration. To reduce variance between the ten model iterations, the habitat suitability predictions and AUC values were averaged. The subsequent predictions were visualised as an average prediction of the five models weighted by the AUC of each model (Figure S3). We opted against an ensemble model approach because preliminary analyses found that an ensemble model based on the MaxEnt algorithm, along with random forest, boosted tree regression and generalized linear models made predictions that were deemed biologically much less feasible than those based on the MaxEnt algorithm itself. For example, the ensemble model was less successful at predicting suitable habitats at locations where the tick has already established in southern California. Conversely, the Death Valley National Park, which has extremely hot and arid climate with considerable temperature fluctuations, and therefore biologically unsuitable for this tick species, was predicted as one of the most suitable areas (Figure S3).


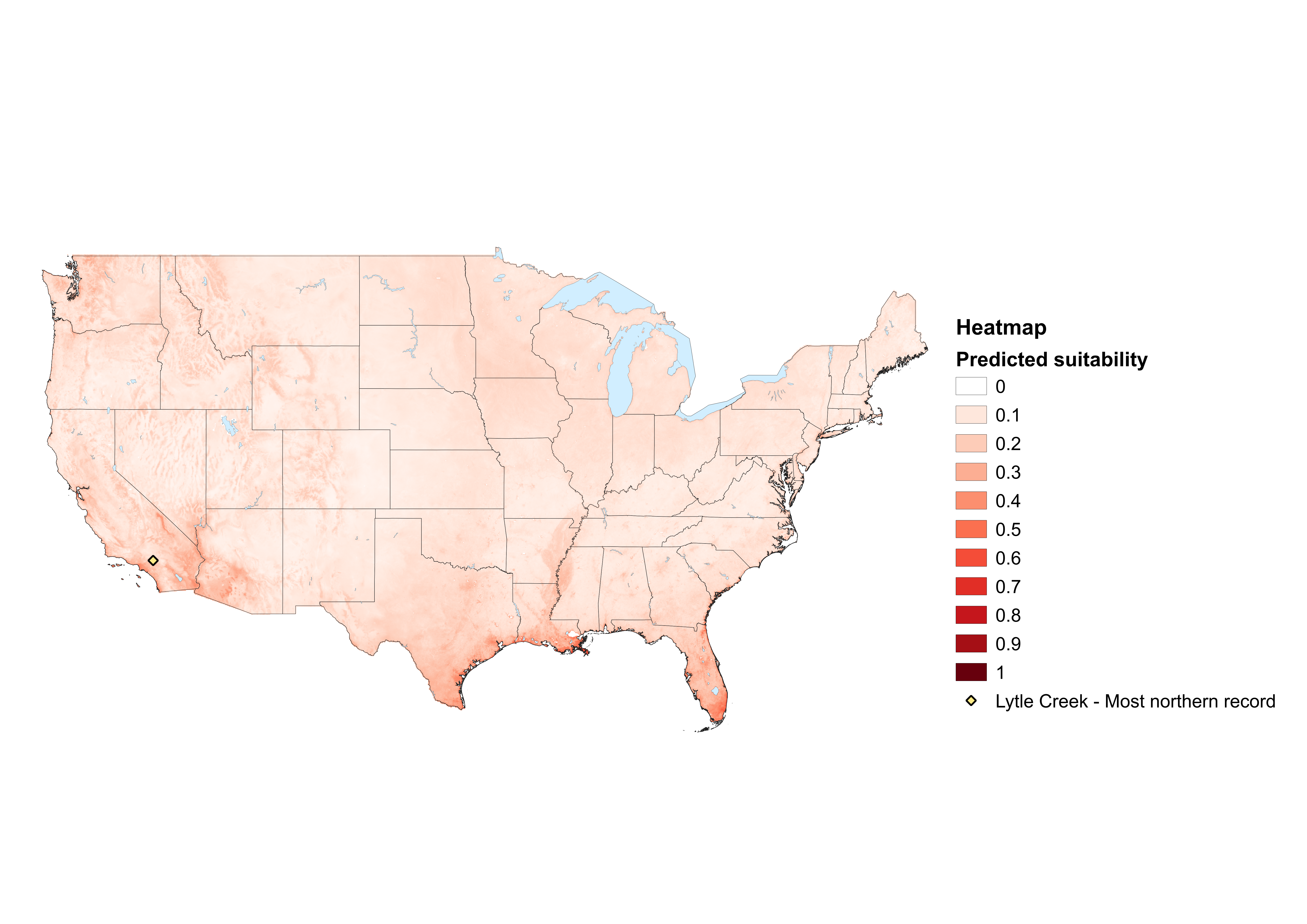


**Figure S3. Tropical lineage *Rhipicephalus sanguineus* suitability maps resulting from ensemble modeling.** Habitat in the U.S. predicted (by an ensemble species distribution model comprising MaxEnt, random forest, boosted tree regression and generalized linear models) to be environmentally suitable for ticks molecularly confirmed to be the tropical lineage of *Rhipicephalus sanguineus* under current (2015–2019) climatic conditions. Suitability is visualized as a heatmap of the average prediction of the five models weighted by the AUC of each model.

**References**

1. Moraes-Filho J, Marcili A, Nieri-Bastos FA, Richtzenhain LJ, Labruna MB. Genetic analysis of ticks belonging to the *Rhipicephalus sanguineus* group in Latin America. Acta Trop. 2011;117: 51–55. doi:10.1016/j.actatropica.2010.09.006

2. Dantas-Torres F, Latrofa MS, Annoscia G, Giannelli A, Parisi A, Otranto D. Morphological and genetic diversity of *Rhipicephalus sanguineus* sensu lato from the New and Old Worlds. Parasites & Vectors. 2013;6: 213. doi:10.1186/1756-3305-6-213

3. Zemtsova GE, Apanaskevich DA, Reeves WK, Hahn M, Snellgrove A, Levin ML. Phylogeography of *Rhipicephalus sanguineus* sensu lato and its relationships with climatic factors. Exp Appl Acarol. 2016;69: 191–203. doi:10.1007/s10493-016-0035-4

4. QGIS Development Team. QGIS Geographic Information System. Open Source Geospatial Foundation Project; 2019. Available: <https://qgis.org/en/site/>

5. Naimi B, Araújo MB. sdm: a reproducible and extensible R platform for species distribution modelling. Ecography. 2016;39: 368–375. doi:10.1111/ecog.01881
