# Supplementary figures and images for: Predicting the northward expansion of tropical lineage Rhipicephalus sanguineus sensu lato ticks in the United States and its implications for medical and veterinary health
Source: PLoS One. 2022 Aug 24;17(8):e0271683. doi: 10.1371/journal.pone.0271683 (PMC9401143; doi:10.1371/journal.pone.0271683)

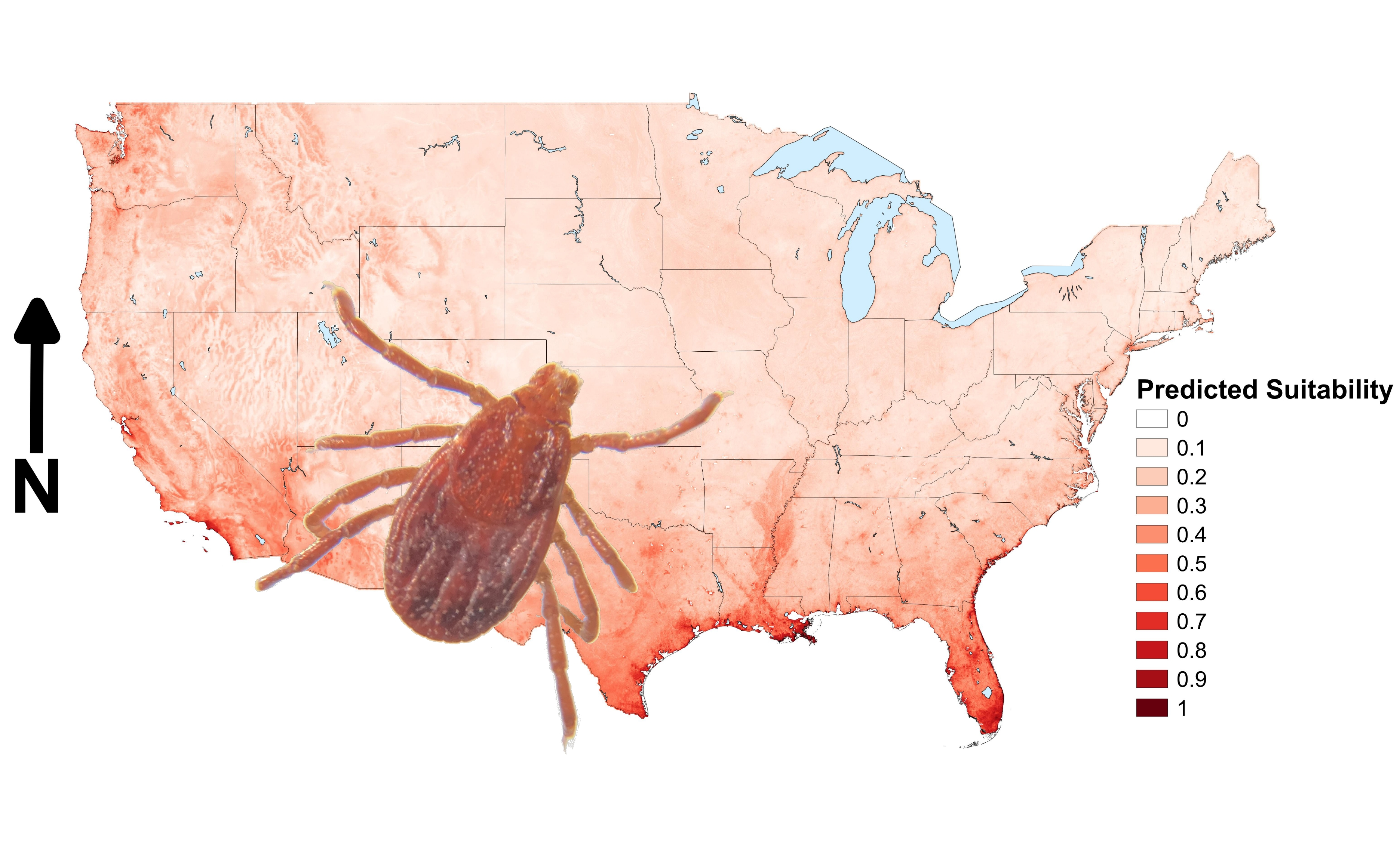

Supplement: S1 Graphical abstract — (TIF) [file pone.0271683.s002.tif]
